# Ecological flexibility and adaptation to past climate change in the Middle Nile Valley: A multiproxy investigation of dietary shifts between the Neolithic and Kerma periods at Kadruka 1 and Kadruka 21

**DOI:** 10.1371/journal.pone.0280347

**Published:** 2023-02-02

**Authors:** Charles Le Moyne, Patrick Roberts, Quan Hua, Madeleine Bleasdale, Jocelyne Desideri, Nicole Boivin, Alison Crowther

**Affiliations:** 1 School of Social Science, The University of Queensland, Saint Lucia, QLD, Australia; 2 Department of Archaeology, Max Planck Institute of Geoanthropology, Jena, Germany; 3 isoTROPIC Research Group, Max Planck Institute of Geoanthropology, Jena, Germany; 4 Australian Nuclear Science and Technology Organisation (ANSTO), Kirrawee DC, NSW, Australia; 5 Department of Archaeology, University of York, York, United Kingdom; 6 Laboratory of African Archaeology and Anthropology, Section of Biology, University of Geneva, Geneva, Switzerland; New York State Museum, UNITED STATES

## Abstract

Human responses to climate change have long been at the heart of discussions of past economic, social, and political change in the Nile Valley of northeastern Africa. Following the arrival of Neolithic groups in the 6^th^ millennium BCE, the Northern Dongola Reach of Upper Nubia witnessed a cultural florescence manifested through elaborate funerary traditions. However, despite the wealth of archaeological data available from funerary contexts, including evidence for domesticated animals and plants as grave goods, the paucity of stratified habitation contexts hinders interpretation of local subsistence trajectories. While it is recognised archaeologically that, against the backdrop of increasing environmental deterioration, the importance of agriculture based on Southwest Asian winter cereals increased throughout the Kerma period (2500–1450 BCE), the contribution of domesticated cereals to earlier Neolithic herding economies remains unclear. This paper presents direct dietary data from a total of 55 Middle Neolithic and Kerma period individuals from Kadruka 21 and Kadruka 1. Microbotanical data obtained from human dental calculus and grave sediments are integrated with human and faunal stable isotopes to explore changes in dietary breadth over time. The combined results demonstrate the consumption of wild plant species, including C_4_ wetland adapted grasses, by Middle Neolithic individuals at Kadruka 1. Despite existing evidence for domesticated barley in associated graves, the results obtained in this study provide no clear evidence for the routine consumption of domesticated cereals by Middle Neolithic individuals. Rather, direct microparticle evidence for the consumption of Triticeae cereals is only associated with a single Kerma period individual and corresponds with an isotopic shift indicating a greater contribution of C_3_-derived resources to diet. These results provide evidence for Neolithic dietary flexibility in Upper Nubia through the persistence of foraging activities and support existing evidence linking increased agricultural reliance to the development of the Kerma culture.

## Introduction

The development and subsequent spread of food production marks a fundamental evolutionary transition in human history. In northeastern Africa, this transition initially concerned the introduction of domesticated animals from Southwest Asia during the late 7^th^ millennium BCE and is often linked to the 8.2k BP arid event (ca. 6300 BCE) [1–5]. It is increasingly clear, however, that local transitions were protracted and highly selective with limited evidence for domesticated animals across the region prior to the 5^th^ millennium BCE [2, 6–10]. Rather, widespread evidence of domesticated animals postdates the end of the early-mid Holocene climatic optimum characterised locally by the southward displacement of the African monsoon belt from ~5300 BCE [11–13]. Following the convergence of populations in ecological refugia, domesticated animals feature prominently in increasingly elaborate funerary traditions associated with the establishment of early herders in the Nile Valley during the 5^th^ millennium BCE [2, 7, 12, 14, 15]. Excepting rare finds of domesticated Southwest Asian crops in the Nile Valley associated primarily with funerary contexts, 5^th^ millennium BCE evidence for farming in northeastern Africa is restricted to the Fayum and Nile Delta [4, 5, 16–21]. As such, it is generally accepted that agriculture represented a later addition to Nile Valley economies, with evidence indicating gradual emergence over the course of 4^th^ millennium BCE in association with the development of the first complex Nile Valley polities (Naqada, A-Group and pre-Kerma cultures) [4, 5, 22–25].

**Fig 1:**
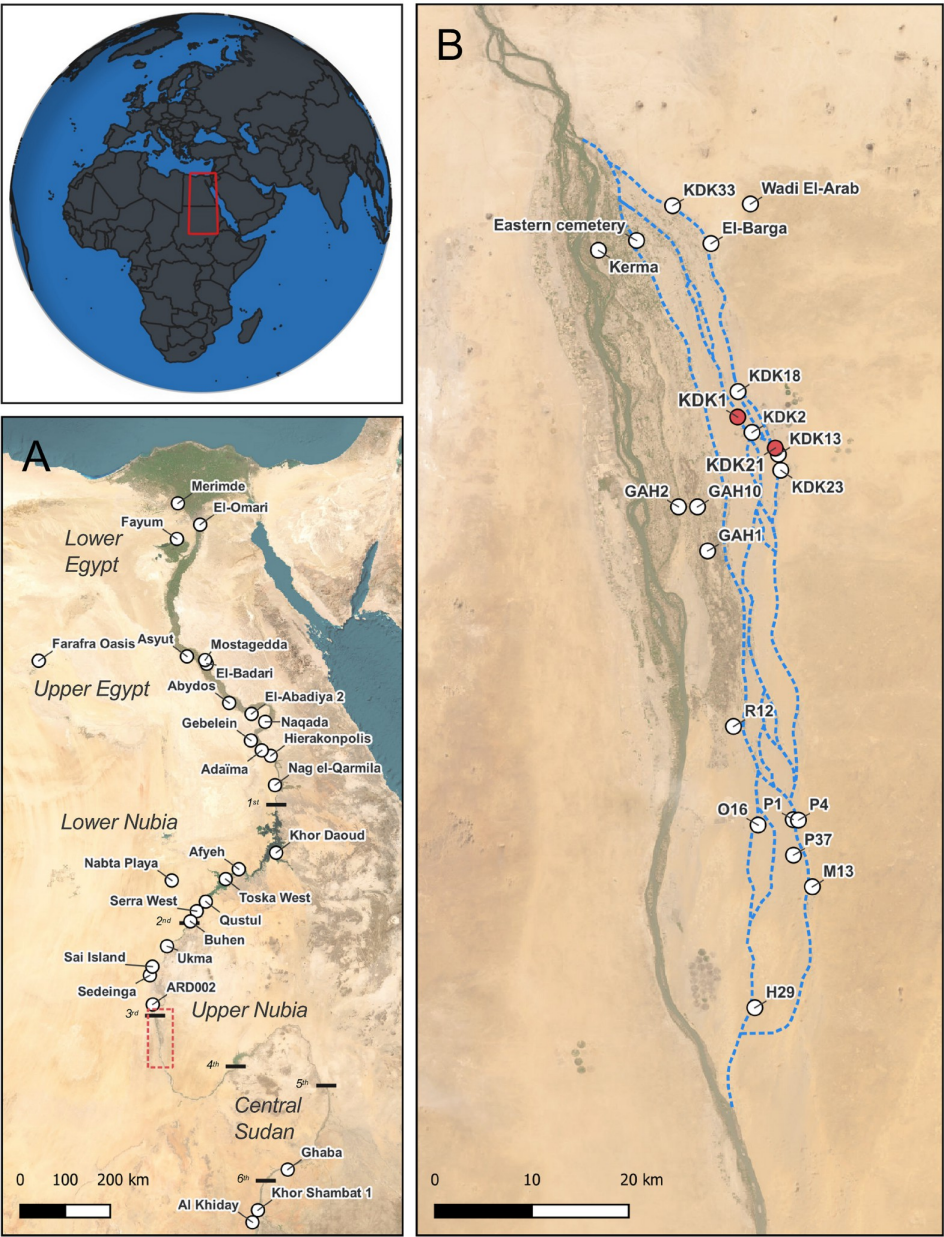
Locations of sites and regions discussed in the text. (A) Map of Nile Valley, red inset indicates location of panel B; (B) Map of alluvial plain and palaeochannel systems in the Northern Dongola Reach of Upper Nubia, study sites KDK21 and KDK1 indicated in red, palaeochannels indicated in blue. World Imagery base map sourced from: Esri, Maxar, Earthstar Geophysics, and the GIS User Community (ArcGIS Pro Licence 3.0).

Evidence for the arrival of Neolithic food producing groups in the Northern Dongola Reach of Upper Nubia, situated in the Middle Nile Valley (Fig 1), dates to the 6^th^ millennium BCE and coincides with Nile channel and floodplain contraction between 6250 and 5750 BCE [26–29]. At the sites of El-Barga and Wadi El-Arab on the desert plateau, human remains with more gracile skeletal morphology appear alongside new classes of grave goods, new building techniques and some limited evidence for food production in the form of a single cattle bucranium at El-Barga [28, 30]. With increasing aridity and further contraction of floodplains in the late 6^th^ millennium BCE, Neolithic populations shifted from the desert plateau to occupy the alluvial plain [27, 29]. Extensive surface scatters indicate widespread occupation along palaeochannel margins, while numerous cemeteries, as observed throughout the wider Nile Valley, attest to the elaboration of funerary traditions over the course of the 5^th^ millennium BCE [27, 31, 32].

Characterised by the increasing presence of domesticated animals and indications of an emergent social hierarchy, these Neolithic funerary traditions provide insight into the local emergence of the agropastoral kingdom of Kerma [33]. Despite the richness of these funerary assemblages, our understanding of Neolithic mobility and subsistence economies for this region of the Middle Nile Valley remains poor. Few Middle Neolithic habitations with stratified domestic contexts necessary for the investigation of subsistence economies are preserved on the alluvial plain. Consequently, archaeological research has focused on cemeteries, which has enhanced ideas of increased transhumance and emphasised the central role of herding economies during the 5^th^ millennium BCE [4, 5, 34–36]. Although rare archaeobotanical finds in funerary contexts indicate that Neolithic populations had access to Southwest Asian domesticated crops [37–41], it is difficult to reconcile the social actions manifested in symbolic contexts with routine economic practice [42]. Convincing evidence for local agricultural production is associated with the later pre-Kerma period (3500–2500 BCE) with site 8-B-52A on Sai Island consisting of numerous storage pits in which macrobotanical crop remains were occasionally preserved [43–45].

To advance our understanding of the role of plants in these early food producing economies and explore diachronic changes in dietary composition, we analysed and integrated archaeobotanical data from dental calculus with stable isotopic data from Middle Neolithic and Kerma period individuals from the cemeteries of Kadruka 21 and Kadruka 1. To ensure confident dietary interpretations, background environmental assessments of microparticles in sediments and enamel diagenetic tests using Fourier Transform Infrared Spectroscopy were also undertaken. The integration of high-resolution taxonomic classifications derived from microbotanical data in dental calculus [46–49], with broader stable isotopic signatures of plant reliance obtained from skeletal tissues (i.e. the relative dietary importance of C_3_/C_4_ plants or of animals consuming C_3_/C_4_ plants), provide different, but complementary, perspectives on diet. Existing stable isotope studies of Middle Nile Valley populations have highlighted the contribution of C_4_ plants to Neolithic diets [50, 51] and their continued importance to Kerma period populations compared to Egyptian groups [52–55]. Although previous analyses of archaeological dental calculus from Middle Nile Valley human remains have built on these stable isotope studies and provided high-resolution glimpses into past subsistence [37, 38, 41, 56–58], this study is the first to directly integrate these approaches in the study of a Middle Nile population and apply them to a relatively large sample size.

### Archaeobotanical evidence in Upper Nubia

The legacy of decades of archaeological research focusing on Neolithic cemeteries in lieu of the more ephemeral settlements has made understanding economic trajectories in Upper Nubia challenging [59, 60]. This gap, while largely attributable to the limited preservation of stratified habitation contexts, is compounded by the inadequate application of systematic archaeobotanical recovery techniques [25]. Consequently, aspects of subsistence such as the persistence of foraging and the timing of the first Southwest Asian winter crop production are poorly understood [25, 45, 59, 61, 62]. More recently, findings of decayed ‘vegetal’ material underlying graves [39, 40] as well as small-scale studies of microbotanical remains such as phytoliths from similar deposits, human dental calculus [37, 38, 41], and sediments recovered from pottery [63] are starting to provide insights into Neolithic plant use in Upper Nubia.

Botanical evidence from the sites of Kadruka (KDK) 1 and R12 in Upper Nubia and Ghaba in Central Sudan suggest that Neolithic groups in the Middle Nile had access to Southwest Asian domesticated cereals by the 5^th^ millennium BCE and potentially earlier during the late 6^th^ millennium BCE, necessitating a reconsideration of the timing of crop introductions to northeastern Africa [37–39, 41]. Based on radiocarbon dates obtained on phytoliths in a single grave at R12 and two graves at Ghaba [37], a 6^th^ millennium BCE or even earlier introduction of these cereals to the Middle Nile has been proposed [37, 38]. Some see this as evidence for the introduction of a Neolithic ‘package’ with crop production characterising initial Neolithic economies [59, 64]. As raised by Fuller and Lucas [25], however, the older dates obtained from phytoliths (5311–5066 BCE grave 46 at R12; 5620–5480 BCE grave 233 at Ghaba) are chronological outliers when considered within the established radiocarbon sequences for the initial use of each site (R12 = 4920–4450 BCE; Ghaba = 4750–4350 BCE) [64, 65]. Recent studies have demonstrated that radiocarbon dates on phytoliths can be unreliable if inappropriate extraction techniques are used, owing to the contribution of older soil carbon as well as the inadequate removal of extraneous materials and organic matter [66, 67]. These studies have cautioned against accepting outlier radiocarbon dates obtained from phytoliths using standard protocols such as those reported for R12 and Ghaba [66–68]. It is also worth noting that, excepting the phytolith date from grave 46 at R12, all published 6^th^ millennium BCE Neolithic sites in the Northern Dongola Reach are located either on the desert plateau or along the eastern fringes of the alluvial plain (KDK33, El-Barga and Wadi El-Arab), whereas sites on the alluvial plain date from the 5^th^ millennium BCE onwards [27, 31, 69]. Similarly, barring the phytolith date from grave 233 at Ghaba and some equally problematic dates at other sites, it is generally accepted that Neolithic food producing groups arrived in Central Sudan at the start of the 5^th^ millennium BCE [70–72]. As such, the appearance of domesticated crops in the Middle Nile should perhaps be re-evaluated in the context of the proliferation of Neolithic sites during the 5^th^ millennium BCE, which is broadly contemporaneous with the establishment of farming in the Nile Delta and Fayum [25]. However, as this evidence remains restricted to funerary assemblages and small quantities of wheat/barley (Triticeae) starch granules and phytoliths in dental calculus and burial sediments, the importance of crops to Neolithic subsistence economies in the Middle Nile remains contentious. Some scholars have argued for the early establishment of an agropastoral economy [39, 59, 64], while others have suggested that these archaeobotanical finds likely represent trade commodities [45, 73] or, at most, small-scale cultivation by seasonally mobile herders [25].

### The Kadruka 21 and Kadruka 1 cemeteries

The archaeological sites of KDK21 and KDK1 are located in the Northern Dongola Reach of Upper Nubia, approximately 20km southeast of the archaeological site of Kerma and near the modern-day town of Kadruka (Fig 1). The sites were first investigated during survey and rescue excavations by *la Section française de la direction des antiquités au Soudan* (SDFAS), directed by Jacques Reinold, between 1986 and 1999 [31, 33, 40, 74]. They are part of a larger site complex consisting of numerous cemeteries and remnants of past human occupations primarily dating to the 5^th^ millennium BCE [31, 75]. As observed elsewhere in the Northern Dongola Reach [27, 32], dense surface scatters of pottery and lithic artifacts attested to extensive Neolithic occupation on terraces above the braided palaeochannels of the Wadi el-Khowi [31]. Seventeen cemeteries situated on natural mounds were also recorded, with subsequent excavations focusing on six of these (KDK1, KDK2, KDK13, KDK18, KDK21 and KDK22) [31]. Particularly evident at KDK1, rich funerary assemblages containing various exotic commodities including–polished mace-heads and personal adornments made from Red Sea shells, amazonite, carnelian and natrolite as well as a single example of a glazed enstatite bead–point to the presence of emergent hierarchies participating in wide-ranging exchange networks [31, 33, 39, 74, 76, 77]. Since 2014, investigations at Kadruka have focused on the reanalysis of previously excavated material [77–79] in addition to the excavation of cemetery KDK23 and nearby habitations [63, 75, 80, 81].

KDK21 is a large burial mound dating to the early- to mid 5^th^ millennium BCE (see Discussion of radiocarbon dates below). Containing ~300 burials, it is one of the oldest investigated Middle Neolithic burial mounds within the Northern Dongola Reach [31]. While the excavation results from KDK21 are largely unpublished, recent reanalysis of the skeletal remains indicate that at least 234 individuals were excavated, though the remains are generally poorly preserved and highly fragmented [78, 79, 81]. The funerary mound was initially interpreted as anthropogenically raised with burials arranged around a central double burial [31], however, re-examination has revealed no clear spatial arrangement of burials and indicates that the mound is more likely natural [78].

KDK1 is a multiphase cemetery that also belongs to the Middle Neolithic period but is slightly younger than KDK21, dating to the mid- to late 5^th^ millennium BCE (see Discussion of radiocarbon dates below) [31, 33, 40]. It is estimated that 115 Middle Neolithic individuals were buried at the site in a broadly concentric arrangement [78, 79]. Interspersed amongst these burials were 37 individuals representing a period of reuse of the cemetery during the later Kerma period [31, 78, 82]. In contrast to the often poorly preserved and highly fragmented Middle Neolithic human remains, Kerma period burials at KDK1 were well preserved with numerous organic materials recovered including human hair and leather grave goods [33, 39, 74].

Differences in grave good assemblages between these sites and other cemeteries dating to the 5^th^ millennium BCE indicate the increasing proliferation of funerary paraphernalia and social markers [27, 31, 40]. For example, differences in the number of graves with cattle bucrania between KDK21 (20 graves, ~7%) and KDK1 (57 graves, ~52%) [31, 76, 78] attest to increasing symbolic and ideological values associated with domesticated fauna over the course of the Neolithic [83]. Additionally, the dietary role of secondary animal products is indicated by the presence of perforated bowls at KDK1 that are comparable to Southwest Asian cheese-strainers [33], as well as proteomic evidence for the consumption of milk obtained from the dental calculus of two KDK21 and KDK1 individuals [56].

Articulated spikes and chaff of domesticated barley found underlying an unspecified number of Middle Neolithic individuals at KDK1 provide a glimpse into early interactions with introduced cereals (see published image in Reinold 2000:87). In addition to the presence of ‘sickles’ in funerary assemblages and their inferred use [82], these cereal finds from KDK1 represent a crucial component of the currently limited Neolithic evidence for domesticated plants in the Middle Nile Valley [59]. As such these oft-cited finds have variously been interpreted as trade commodities or hallmarks of emerging agropastoral economies [25, 38, 45, 59, 60]. However, the association of the KDK1 botanicals with burials rather than domestic contexts leaves the interpretation of their subsistence importance open.

### A multiproxy approach to reconstructing past diet

In recent decades, studies of plant microparticles and other biomarkers preserved in human dental calculus (mineralised plaque) have provided a new window into often inaccessible aspects of past human diet [46–49]. Initial applications of this methodology in the Middle Nile Valley have started to transform our understanding of Neolithic subsistence economies and dietary breadth [37, 38, 41, 56–58]. However, there is increasing recognition of the interpretive constraints that limit the utility of these analyses as a proxy for past diet [84–86]. Studies continue to highlight the variability of recovered microparticle assemblages that present a partial record of dietary intake and fail to capture dietary breadth at the individual level [87, 88]. As such, dental calculus studies are increasingly applying larger population approaches embedded in multiproxy frameworks [57, 58, 89–92].

Bulk stable isotopic analyses of human and animal tissues are widely applied proxies in the reconstruction of ancient food webs and dietary breadth [93–96]. ẟ^15^N and ẟ^13^C analyses of bone and tooth collagen provide information regarding trophic level in addition to the contribution of C_3_ (e.g., trees, shrubs, temperate grasses and domesticated Southwest Asian cereals) and C_4_ (e.g., tropical grasses) resources to diet. The stable carbon values of C_3_ and C_4_ plants have a bimodal distribution ranging between -35‰ to -19‰ and -13‰ to -8‰, respectively [93, 97]. These distinct sets of values are passed onto consumers with a known fractionation in collagen of roughly +5‰ [98, 99]. While ẟ^13^C collagen values preferentially reflect high protein sources such as animal foods, ẟ^13^C carbonate values obtained from tooth enamel hydroxyapatite reflect total dietary input (i.e. carbohydrates, lipids and proteins) and are therefore more representative of overall consumption [100, 101].

ẟ^15^N values from bone and dentine collagen relate to the trophic level of consumers, with an increase of 3–5‰ per trophic level observed in both terrestrial and aquatic ecosystems [102, 103]. Larger food webs in aquatic systems are linked to higher ẟ^15^N enabling differentiation from terrestrial sources. Environmental factors can impact baseline ẟ^15^N in a given ecosystem. Increased ^15^N enrichment in consumers, and to a lesser degree in plants, is linked to aridity through the preferential loss of ammonia in soils [104]. The resulting baseline increase in ẟ^15^N is then enhanced in the tissues of drought adapted herbivores, particularly non-obligate drinkers and browsers, through the excretion of ^15^N depleted urea [105, 106]. Although generally associated with greater consumption of animal products or aquatic resources, intensive crop management (manuring) in agricultural contexts can also cause elevated ^15^N in humans [107, 108].

ẟ^18^O values measured on enamel hydroxyapatite reflect imbibed water as well as water and organic-bound oxygen derived from consumed plants and animals, thus providing information regarding mobility and past climatic conditions [109]. As stable oxygen isotope ratios in water are determined by local hydrological, geographical and climatic factors, ẟ^18^O values obtained from tooth enamel represent the environment in which the animal or person lived during tooth formation [109]. Reduced humidity in arid environments result in the enrichment of ^18^O in surface water and plant leaves through evaporation and evapo-transpiration, respectively [110, 111]. This enrichment is preferentially reflected in non-obligate drinkers obtaining higher proportions of body water from plants that are ^18^O enriched from preferential evapo-transpiration of the lighter ^16^O [111–113]. By integrating stable isotopic and plant microparticle data from a representative sample size, this study enables a more nuanced understanding of diachronic changes in dietary composition.

## Materials and methods

### Permission to conduct research

All necessary permits were obtained for the described study, which complied with all relevant regulations. As part of the Cultural Agreement Protocol established in 1969 between *la Section française de la direction des antiquités au Soudan* (SFDAS) and the National Corporation for Antiquities and Museums (NCAM), Khartoum, Sudan, archaeological samples from KDK21 and KDK1 were exported by Jacques Reinold (former director of SFDAS and excavator of the sites) between 1986 and 1999. In collaboration with Christian Simon (project bioarchaeologist) and Louis Chaix (project zooarchaeologist), the human and faunal remains were curated at the University of Geneva and the Museum of Natural History, Geneva, for further study. All samples analysed in this study were obtained for destructive analysis under the terms of an agreement with SFDAS and the University of Geneva. Ethical approvals were obtained from the Australian Government National Health and Medical Research Council (NHMRC), Human Research Ethics Application (HREA) #2018001148 and #2020002401. The microparticle extracts analysed in this study are currently stored at The University of Queensland Archaeology Laboratories. All remaining samples not destroyed in the study have been returned to the University of Geneva and the Museum of Natural History, Geneva.

### Archaeological samples

Samples from 55 human burials in addition to associated sediments and fauna from the sites of KDK21 and KDK1 were analysed (Table 1). These were obtained from the collections curated by the Commission of Anthropological Collections at the University of Geneva, Faculty of Sciences, Geneva (human remains) and the Museum of Natural History, Geneva (fauna).

**Table 1:** Analysed samples from KDK21 and KDK1.

Site	Phase	Dental calculus samples	Sediment samples	Human tooth samples	Faunal samples
Kadruka 21	Middle Neolithic	24	3	10	-
Kadruka 1	Middle Neolithic	22	4	11	-
	Kerma	9	-	9	7
	Total:	55	7	30	7

Refer to S1, S2 Tables in S1 File for individual specimen information.

**Fig 2:**
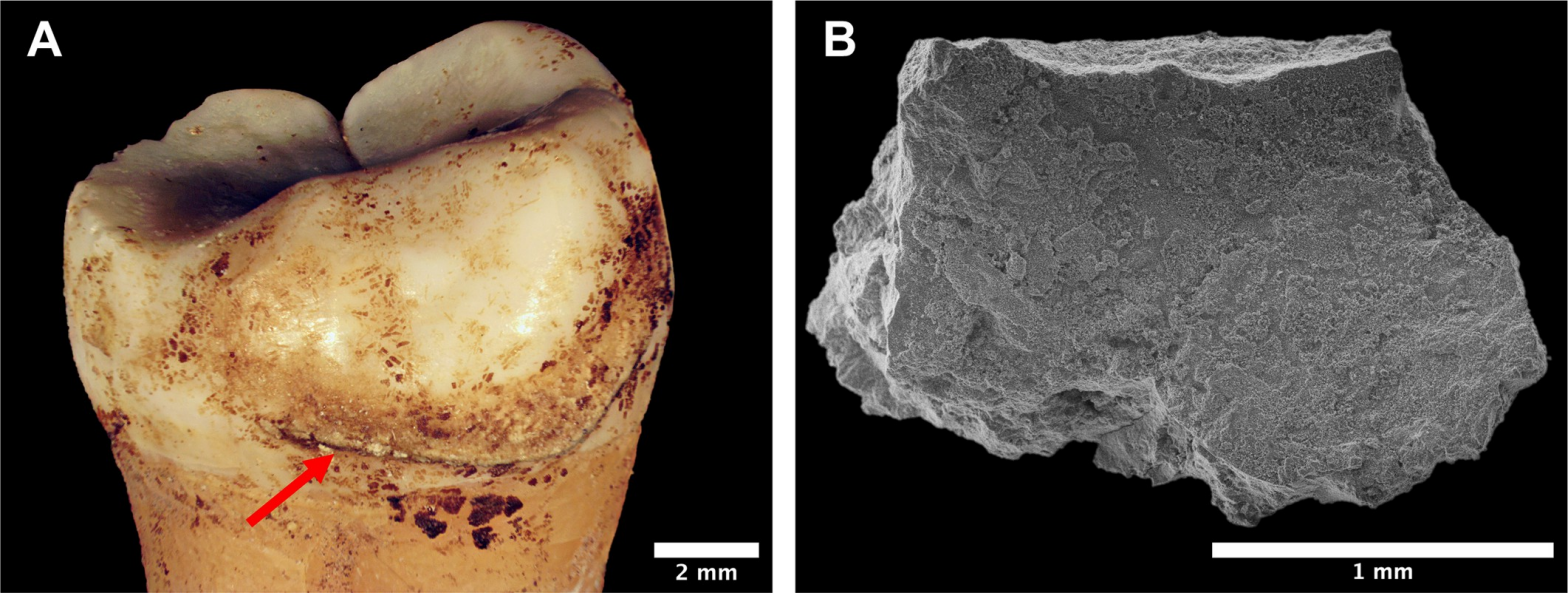
Example of in situ calculus deposit selected for analysis. (A) Supragingival calculus deposit (indicated by red arrow) on buccal surface of right M_3_, individual SK 63, KDK1; (B) Proximal surface (surface previously adhering to tooth) of sampled calculus from same individual under SEM.

Calculus deposits were visually inspected to ensure no adhering soil was present, with suitable deposits from 55 individuals photographed in situ (Fig 2A). A single deposit from each individual was selected for sampling with the location of each sample recorded. Selected deposits were then removed using a sterilised dental scaler, weighed and transferred to microcentrifuge tubes.

During sampling, differences in the structural integrity of dental calculus deposits were observed. While most calculus deposits remained consolidated and came off the tooth as a single piece, some were more friable and crumbled, particularly along the outer margins of the deposits. These differences in the structural morphology of calculus matrices may reflect increased diagenetic susceptibility of microparticles embedded in friable samples, compared to the ‘stable’ calculus microenvironment commonly reported in the literature [46, 89, 114]. This may also have implications regarding authenticity of recovered microparticles; for example, if they are restricted to these more friable dental calculus samples they may indicate exogenous contamination. Therefore, the dental calculus samples were classified according to observed differences in structural composition and size (easily disaggregated powder <0.5 mm or large solid pieces >0.5 mm). These observations were made using a low-power Olympus SZ61 stereozoom microscope (6–45x). Powder and solid fractions from the same sample were analysed separately to provide insight into the distribution of microparticles within the calculus matrix and help clarify the impact of post-depositional processes. Following the recommendations of Power et al. [115], SEM imaging using a Hitachi TM3030 (80–1000x) was conducted on large solid calculus samples to capture surface morphology and assess the presence of exogenous microparticles (Fig 2B). Sediments adhering to the crania of seven individuals were also collected to assess background starch and phytolith signatures in the burial environments (S1 Table in S1 File).

Teeth from 30 of the individuals sampled for dental calculus analysis were also sampled for stable isotope analyses (δ^15^N, δ^13^C, δ^18^O) and radiocarbon dating (S1 Table in S1 File). To avoid stable isotopic influences from weaning, third molars (which commence forming between 7–10 years) were sampled for all individuals except two adolescents that only had second molars (commence forming between 2.5–3 years) available [116]. Caprine bones (n = 5) and teeth (n = 2) recovered from KDK1 Kerma period grave good assemblages [117] were sampled for radiocarbon dating and to provide a stable isotopic baseline (S2 Table in S1 File). Matching observed patterns at the nearby Eastern Cemetery [118–120], the domesticated caprines excavated from the Kerma period graves at KDK1 are immature specimens [117]. Unfortunately, as these are the only fauna available for this study, this necessitated sampling of immature specimens for stable isotopic analyses.

### AMS radiocarbon dating of collagen

Based on visual inspection, 20 of the 30 human teeth sampled for stable isotopic analyses of enamel were taken forward for collagen extraction; the remaining 10 teeth were not suitably preserved to warrant attempted collagen extraction from dentine. These 20, in addition to five Kerma period faunal bone samples, were pre-treated at the Radiocarbon Laboratory at the Australian Nuclear Science and Technology Organisation (ANSTO) for stable carbon and nitrogen isotope analyses, and for radiocarbon dating. In brief, the samples were cleaned with a drill, washed with deionised water, dried and then crushed. The crushed samples were pre-treated using the acid-base-acid (ABA) method followed by gelatinisation in a pH3 hydrochloric acid (HCl) solution at 75°C for 20 h and ultrafiltration using pre-cleaned Millipore 30kD ultrafilters [121–123]. If only a small amount of collagen solution remained after gelatinisation, the ultrafiltration step was not applied. The >30kD or full (without ultrafiltration) collagen solution of each sample was then freeze dried, a small portion of which was used for C/N, ẟ^13^C and ẟ^15^N analyses (see below section on stable isotope analysis).

The remaining (either >30kD or full) collagen of each sample with an acceptable C/N ratio between 2.9–3.6 [124–126] was combusted and then converted to graphite for radiocarbon analysis by accelerator mass spectrometry (AMS) [127]. Radiocarbon analysis was carried out using the Vega AMS Facility at ANSTO [128]. Age calibration was performed using the IntCal20 Northern Hemisphere calibration curve [129] and OxCal program, version 4.4.2 [130].

Previously published radiocarbon dates, including the directly-dated Kerma period individual SK 68 from KDK1 [56] and Middle Neolithic dates obtained from freshwater *Aspatharia* bivalves at KDK21 and KDK1 [31, 40], were recalibrated as part of this study. Freshwater shells can have older ^14^C ages due to the freshwater reservoir effect (FRE) resulting from the presence of dissolved old carbonates or the so-called hardwater effect [131–133]. However, comparative studies in the Kerma area have indicated that local FRE is negligible [27]. Therefore, no FRE correction was applied when re-calibrating the existing Middle Neolithic dates from KDK21 and KDK1.

A Bayesian chronological model for KDK21 and KDK1 was constructed using the radiocarbon ages of human tooth dentine and faunal bone collagen in the current study together with the published dates from freshwater bivalves and human hair. The model consisted of two contiguous phases representing the Middle Neolithic and Kerma periods, also incorporating prior information from pottery typologies and funerary assemblages at each site regarding the chronological sequence of archaeological groups. The modelling was carried out using the OxCal program, version 4.4.2 [130].

### Extraction and analysis of plant microparticles

Microparticle extractions from dental calculus were performed in accordance with published recommendations for ancient starch research [134, 135]. Samples were processed in a dedicated ancient starch laboratory at The University of Queensland equipped with a HEPA filter and laminar flow hood to minimise environmental contamination. Starch-free gloves and laboratory suits, and autoclaved consumables were used in all procedures. Throughout sample preparation and analysis all workspaces were periodically wiped down with 2% sodium hydroxide (NaOH) to remove surface contaminants [135]. Experimental negative controls (n = 15) were also run during sample decalcification and slide preparation.

Solid calculus samples were first vortexed in reverse osmosis (RO) water to separate any loose exogenous material, with supernatant removed using a pipette once all visible calculus fragments had settled. Each sample was then treated with 1 ml of 2% NaOH for 1 min to destroy residual exogenous starch granules. Samples were rinsed and centrifuged three times with RO water at 13,000 RPM for 5 min with supernatant removed using a pipette between rinses. All samples were then decalcified with 1.5 ml of 0.5 M ethylenediaminetetraacetic acid (EDTA) for 6–24 h depending on individual sample decalcification rates [136, 137]. Once fully decalcified, samples were rinsed and centrifuged three times with RO water at 13,000 RPM for 5 min with supernatant removed using a pipette between rinses.

While representative sediment samples from the areas underlying the graves were not available for this study, small amounts of sediment adhering to the crania of seven individuals were sampled at the University of Geneva to assess background microparticle signatures from the burial environment. The sediments, primarily coarse to fine grained sand aggregates, were sieved through an autoclaved 250 μm mesh to remove larger sand grains. Contamination controls during separation and preparation of starch fractions followed those applied for dental calculus extractions outlined above. Around 0.3–0.4 g was weighed out per sample for separate starch and phytolith extractions. Samples less than 0.3 g in weight were split for these analyses. Phytoliths were extracted using a combination of microwave-assisted acid digestion [138] followed by heavy liquid flotation (S3 Table in S1 File). Starch extractions followed established protocols (S4 Table in S1 File) [139–141].

All calculus and sediment extracts were mounted on pre-weighed, autoclaved microscope slides, dried and reweighed to determine analysed weights. Autoclaved coverslips were attached to slides with clear fingernail varnish. Slides were rehydrated with RO water and scanned at 200–600x with an Olympus BX50 light microscope. Photographs of selected microparticles were taken using a MIchrome 5 pro camera and Mosaic software (V2.0). Calculus samples and starch extracts from sediments were fully scanned under both plane and crossed-polarised light. A total of 200 diagnostic phytoliths were recorded per sediment sample as a baseline for assessing variations in morphotype diversity between sediment and dental calculus assemblages.

Phytolith types were classified and described according to the International Code for Phytolith Nomenclature (ICPN 1.0 and 2.0) [142, 143]. Interdigitate phytoliths from grass inflorescences were classified separately to Elongate types in accordance with established morphological criteria (S1 Fig in S1 File) [144–147]. Starch granules were described using standard morphological variables including three-dimensional shape; hilum position (centric, eccentric, highly eccentric); extinction cross form; presence and form of vacuoles, fissures, lamellae and facets; surface texture; and degree of birefringence [148–152]. These were then classified into morphotypes based on multiple morphological features, and qualitatively compared with published taxa descriptions (S2 Fig in S1 File) [148, 149, 152–154].

Counts of phytoliths, starch granules and other microparticles of interest recovered from dental calculus and sediment samples were presented using Tilia v3.0.1 with cluster analysis [155] used to explore phytolith assemblage diversity between sediment samples. A Jaccard similarity test [156] was performed to assess variation in the composition of phytolith assemblages between correlated dental calculus samples (solid and powder). As this similarity test excludes co-absences it provides a robust indicator of variations in morphotype occurrence. Presenting a range from 0–1, with a result of 0 reflecting the absence of co-occurring morphotypes and a result of 1 signifying identical morphotype distributions between fractions [156], this test was used to establish the degree of intersection between correlated fractions.

Due to its suitability for highly variable archaeobotanical data with many zero values, detrended correspondence analysis (DCA) was performed on the phytolith assemblages from dental calculus to evaluate the distribution of morphotypes between archaeological groups (KDK21 Middle Neolithic, KDK1 Middle Neolithic and KDK1 Kerma) [157, 158]. DCA was applied instead of regular CA to remove the arch effect resulting from the distortion of the second (vertical) axis [159]. To reduce the impact of highly variable samples, raw counts of diagnostic phytoliths were first converted to presence/absence. Associated solid and powder calculus fractions were combined as a single dataset for the purpose of comparing differences in assemblage composition between archaeological groups. Analysis of similarity (ANOSIM) was conducted to measure the significance of observed clustering of archaeological groups by comparing the mean of ranked between group to within group dissimilarities [160].

### Fourier Transform Infrared Spectroscopy (FTIR) assessment of enamel preservation

Bulk enamel samples were obtained from 20 KDK1 individuals and 10 KDK21 individuals to determine stable carbon and oxygen isotope ratios. Enamel fragments from two immature sheep recovered from Kerma period graves at KDK1 were also bulk sampled. Air-abrasion was used to clean selected teeth and tooth fragments of adhering material. Approximately 2–4 mg of enamel powder was then obtained per sample through gentle abrasion of the buccal surface with a DREMEL equipped with a diamond tipped drill bit. To avoid cross-contamination, the drill bit was cleaned with 0.5 M HCl then rinsed with ethanol between each sample. Samples were transferred to 1.5 ml microcentrifuge tubes.

While enamel apatite is more resistant to post-depositional diagenesis than bone apatite, numerous studies have demonstrated that subtle alterations of enamel carbonate environments can occur during fossilization resulting in altered isotopic values [161, 162]. This is particularly pertinent to this study, as Middle Neolithic skeletal remains at KDK21 and KDK1 display clear taphonomic indicators of cyclical humid and dry burial conditions [78, 79] associated with the wetter environmental conditions of the middle Holocene prior to the onset of progressive aridification around 2200 BCE [27, 29, 163–165]. Therefore, to assess diagenetic structural and compositional modification of enamel bioapatite, the crystal-chemical properties of each sample were assessed using Fourier Transform Infrared Spectroscopy (FTIR) prior to stable isotopic pre-treatment (S8, S9 Tables in S1 File). FTIR analysis, which absorbs radiation at discrete vibrational frequencies related to the crystallographic structure of key functional groups, can be used to detect both the presence of contaminant carbonates and changes in enamel crystallinity [162]. Observed absorbance bands can be ascribed to the internal vibrations of molecular groups, phosphates (PO^3^4), carbonates (CO^2^3), and hydroxyl groups (OH), in apatite (S7, S8 Tables in S1 File) [166, 167].

Powdered enamel from each sample was analysed in triplicate between 500 and 4000 cm^-1^ by FTIR with Attenuated Total Reflectance (FTIR-ATR–NICOLET 5700 from Thermo Electron) at the Australian Institute for Bioengineering and Nanotechnology Centre for Advanced Imaging at The University of Queensland. Sample background was subtracted prior to a baseline correction using Spectragryph v1.2.15 software [168]. Spectra baselines were normalised with the replicate spectra for each sample averaged before calculation of infrared indices. The potential for secondary contaminant calcite intrusions was assessed by checking for an absorbance peak at 711 cm^-1^ [162]. To ensure reproducibility of the measurements, only spectra with a minimum absorbance of 0.06 for the highest phosphate band at ~1035 cm^-1^ were considered for calculation of indices. The reproducibilities of the indices BPI, API, BAI, PCI and WAMPI are ±0.02, ±0.008, ±0.09, ±0.02 and ±0.01, respectively. Infrared indices obtained on the archaeological samples were compared with published empirical indices to assess the degree of diagenetic alteration [161, 162, 167, 169–171].

To identify statistically significant differences in enamel crystallinity and structure between the three archaeological human groups that may reflect diagenetic alteration of stable isotopic values, analysis of variance (ANOVA) followed by post-hoc Tukey pair-wise comparisons were conducted for each of the FTIR indices of enamel apatite (PCI, BPI, API, BAI and WAMPI). FTIR indices of the two faunal enamel samples were excluded from these statistical tests.

### Stable isotope analysis of enamel and collagen

Following diagenetic assessment by FTIR-ATR, enamel powder samples were pre-treated following established protocols [172]. Samples were purified using 0.2–0.4 ml (dependent on individual sample weight) of dilute 0.1 M acetic acid for 10 min, followed by three rinses with ultrapure water to neutralise the samples [172, 173]. Individual microcentrifuge tubes were then covered in parafilm, pierced with a small hole and placed in a freeze drier for 6 h. Approximately 1 mg of each sample was placed into phosphoric acid-resistant borosilicate glass vials. Following reaction with 100% phosphoric acid, the stable carbon and oxygen isotope compositions of gases evolved from the samples were determined in the Stable Isotope Geochemistry Laboratory at The University of Queensland using an Elementar Isoprime 100 Dual Inlet Isotope Ratio Mass Spectrometer (DI-IRMS) coupled to a multiprep bench. Stable isotopic values were reported in per mil (‰) relative to VSMOW (Vienna Standard Mean Ocean Water) for oxygen and VPDB (Vienna Pee Dee Belemnite) for carbon. Stable isotopic values were calibrated using international standards NBS18 (ẟ^13^C -5.014 ± 0.035‰, ẟ^18^O +7.20 ± 0.1‰), NBS19 (ẟ^13^C +1.95‰, ẟ^18^O +28.65‰) and USGS44 (ẟ^13^C ~-42.21 ± 0.05‰) via a three-point normalisation for ẟ^13^C and two-point normalisation for ẟ^18^O. Measurement uncertainty was monitored using an internal laboratory calcite standard (BCS) with a well-characterised isotopic composition (ẟ^13^C -3.88 ± 0.03‰, ẟ^18^O +10.3 ± 0.09‰). Precision (1σ) was determined to be ± 0.049‰ for ẟ^13^C and ± 0.079‰ for ẟ^18^O on the basis of repeated measurements of calibration standards and check standards. Accuracy or systematic error was determined to be ± 0.03 for ẟ^13^C and ± 0.098 for ẟ^18^O on the basis of the difference between the observed (n = 12) and known ẟ values of the check standard and the long-term standard deviation of the check standard. However, as the composition of the check standard is more homogenous than enamel, these values likely underrepresent the analytical uncertainty for enamel which is expected to be ~± 0.3 for both parameters based on existing studies.

All ẟ^18^O and ẟ^13^C results from human enamel samples were tested for normality using the Shapiro-Wilk test and histogram observations, prior to application of ANOVA tests to determine whether statically significant differences occurred between groups (KDK21 Middle Neolithic, KDK1 Middle Neolithic and KDK1 Kerma). This was followed by post-hoc Tukey pair-wise comparisons to determine where significant variance occurred between groups in relation to ẟ^18^O and ẟ^13^C values.

C/N, ẟ^13^C and ẟ^15^N ratios of bone and dentine collagen samples extracted for AMS ^14^C were determined using an Elementar vario ISOTOPE Select Elemental Analyser coupled with an Elementar EcovisION Continuous Flow Isotope Ratio Mass Spectrometer (EA-IRMS). C/N ratios were normalised to the reference standard Acetanilide with a precision of 0.1 (1σ). Stable isotopic values were calibrated using the international standards USGS40 (ẟ^13^C -26.38 ± 0.042‰, ẟ^15^N -4.52 ± 0.1‰) and USGS41a (ẟ^13^C +36.55 ± 0.07‰, ẟ^15^N +47.55 ± 0.09‰) via a two-point normalisation for both ẟ^13^C and ẟ^15^N. Acceptable stable isotopic ratios were also carried forward for interpretation of diet with values reported in per mil (‰) relative to AIR (Ambient Inhalable Reservoir) for nitrogen and VPDB for carbon. Measurement uncertainty was monitored using a certified protein (casein) reference standard from Elemental Microanalysis (EM B2155) with a well-characterised stable isotopic composition (ẟ^13^C -26.98 ± 0.13‰, ẟ^15^N +5.83 ± 0.08‰). Precision (1σ) was determined to be ± 0.059‰ for ẟ^13^C and ± 0.067‰ for ẟ^15^N on the basis of repeated measurements of calibration standards and check standards. Accuracy or systematic error was determined to be ± 0.338 for ẟ^13^C and ± 0.18 for ẟ^15^N on the basis of the difference between the observed (n = 7) and known ẟ values of the check standard and the long-term standard deviation of the check standard.

Statistical analyses and data presentation of dental calculus and stable isotope results were conducted using R software [174] with tidyverse, vegan and ggplot2 packages [175–177].

## Results

### AMS radiocarbon results and Bayesian model

Collagen sufficiently preserved for reliable AMS ^14^C analysis (i.e. C/N ratios between 2.9–3.6) was only extracted from seven Kerma period samples (S5 Table in S1 File) [124–126]. None of the Middle Neolithic samples preserved sufficient collagen for dating, likely due to the diagenetic impact of recharge hydrological regimes resulting from the fluctuation of wet and dry periods during the middle Holocene [178–180]. In contrast, progressive aridification in the Northern Dongola Reach from around 2200 BCE [27, 29, 163–165] has facilitated enhanced preservation of Kerma period remains at KDK1. Of the seven samples, one, OZAC37 (SK 26), only had a small amount of material remaining after gelatinisation. The ultrafiltration step was therefore not performed for this sample to maximise the material remaining for radiocarbon dating. The collagen extraction for the other six samples included the final ultrafiltration step. The C/N ratios and radiocarbon results for these seven samples, together with published radiocarbon dates for KDK1 and KDK21 [31, 33, 56], are presented in S5 Table in S1 File. With the exception of faunal sample T95/2, all C/N ratios exceeded the conservative range of 2.9–3.4 expected for animals with a mixed C_3_/C_4_ diet [181]. Higher C/N ratios between 3.4–3.6 in the remaining samples may reflect a degree of contamination likely derived from humics [181, 182]. However, as the local environment was increasingly arid throughout and subsequent to the Kerma period [27, 29], exogenous humic contaminants derived from the decay of organic plant matter are unlikely. Rather, as the AMS radiocarbon dates obtained from these individuals with higher C/N ratios are similar to the date obtained from sample T95/2 (see S5 Table in S1 File) and fall within the expected age based on the material culture [74], elevated C/N ratios likely reflect endogenous humic contribution. Linked to the in situ breakdown of the collagen itself, endogenous humics would not alter the ^14^C date [126, 182], but would have a slight influence on ẟ^13^C dietary values [181].

**Fig 3:**
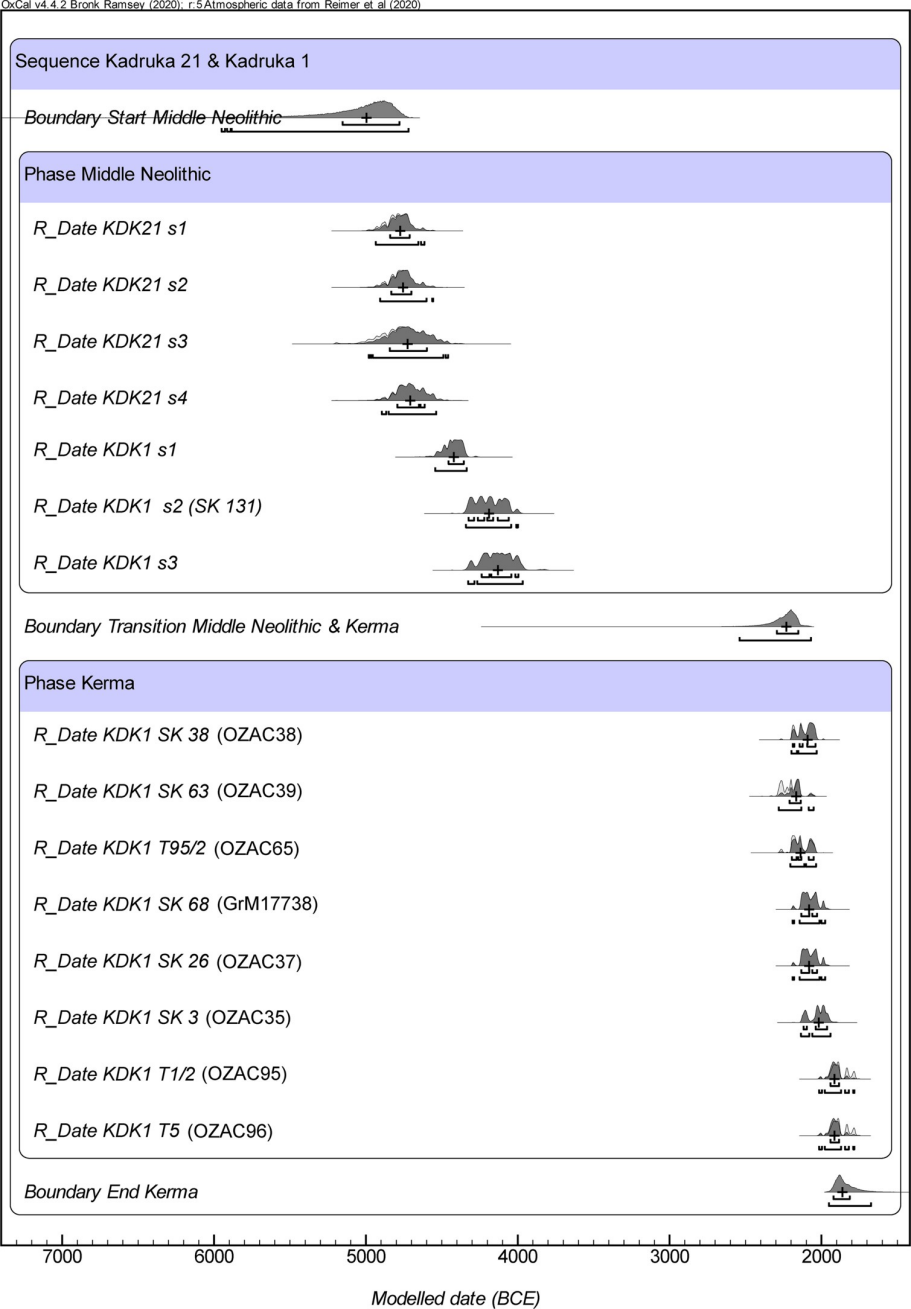
Bayesian model of ^14^C dates from KDK21 and KDK1. Prior distributions (unmodelled calibrations) are shown in light grey. Posterior distributions (modelled dates) are shown in dark grey and the plus symbol indicates the median modelled age. The Kerma ^14^C date for individual SK 68 was obtained from a hair sample [56]. Middle Neolithic ^14^C dates were obtained from *Aspatharia* spp. bivalves included in grave assemblages [31, 40]. Refer to S5 Table in S1 File.

A model for KDK21 and KDK1 was built using the new radiocarbon determinations from human tooth dentine and faunal bone collagen, and the existing determinations from freshwater bivalves and human hair. The model consists of two contiguous phases representing the Middle Neolithic and Kerma periods. The agreement index (A_model_) of the model is 106%, which is much higher than the accepted level of 60% [130], indicating that the posterior estimates conform well to the prior information. The modelling result is presented in S5 Table in S1 File and illustrated in Fig 3. Differences in the distribution of Middle Neolithic dates between KDK21 and KDK1 correlate with the Middle Neolithic A and B periods, respectively [65]. The model indicates that the onset of Kerma burials at KDK1 dates to between 2540–2070 BCE (95.4% CI). The estimate for the end of the Kerma phase at KDK1 dates to between 1952–1674 BCE (95.4% CI). The new radiocarbon determinations for KDK1 confirm the previous relative chronology based on pottery typologies and personal adornments [74], and demonstrate that the period of cemetery reuse occurred at the end of the Kerma *Ancien* (2500–2050 BCE) and continued into the Kerma *Moyen* (2050–1750 BCE) [183, 184]. The estimated duration of the Kerma phase at KDK1 is between 173 and 817 years (95.4% CI).

### Dental calculus

**Fig 4:**
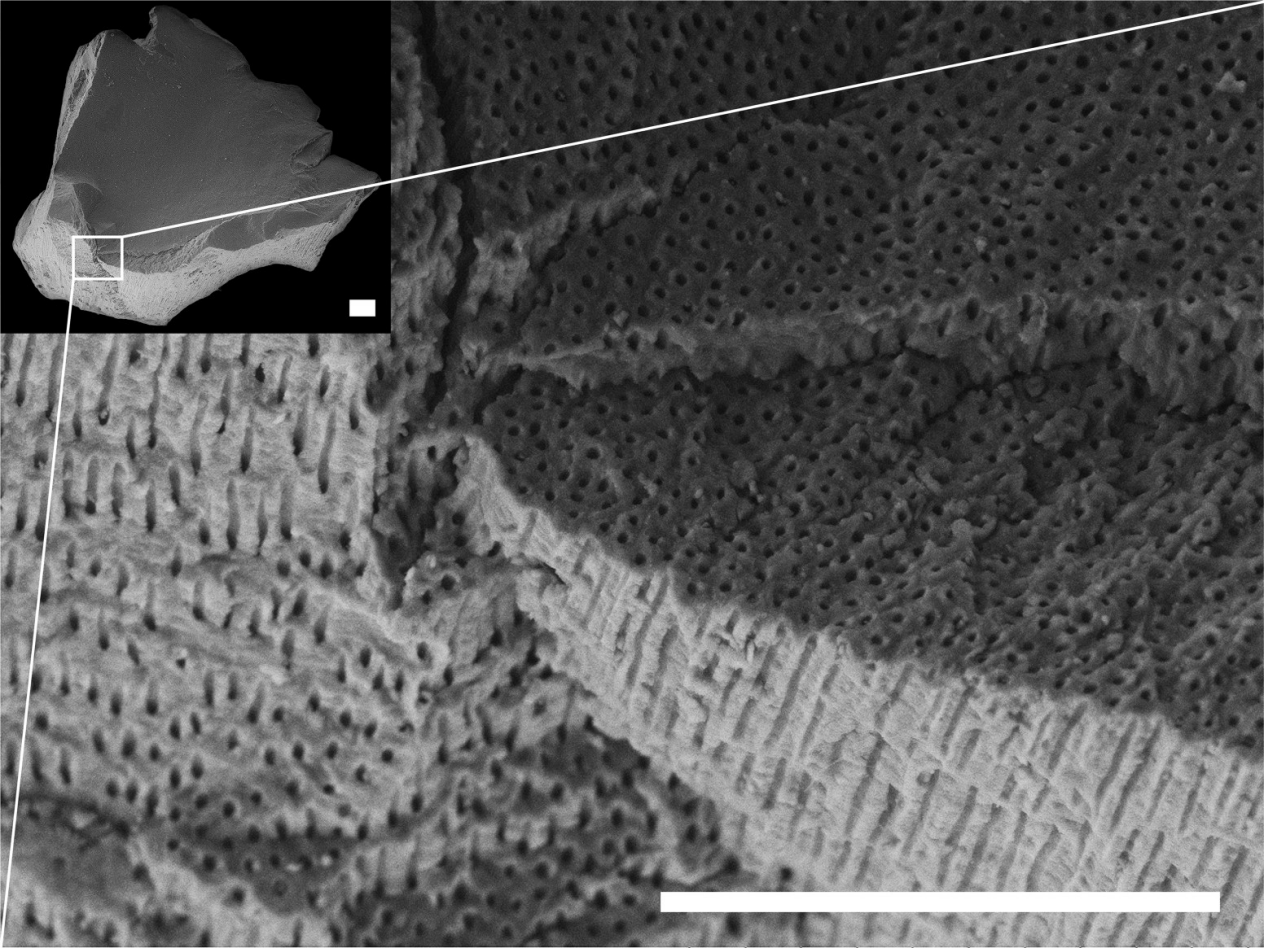
Supragingival calculus sample from lingual surface of right M^1^, individual SK 62, KDK1. Example of dental calculus sample with pores arranged perpendicular to the proximal interface (interface that previously adjoined the tooth surface). Scale bar (100 μm) applies to both panels.

SEM imaging of larger dental calculus pieces (>0.5 mm) revealed a high degree of morphological heterogeneity between samples. Of particular note was the presence of pores, ranging from ~1.1–3.6 μm in diameter, covering the surface previously adjoining the tooth, creating channels that penetrate into the calculus matrix. These pores and channels were present in the entire calculus matrix of five Neolithic (12.5%) and Kerma (22.2%) period samples (SK 24, SK 39, SK 62, SK 87a and SK 96) from KDK1, but were not observed in KDK21 samples (Fig 4). As calculus mineralises in a multi-stage process, the presence of these pores within some archaeological samples may indicate cell lysis (bacterial fratricide) relating to the decay of non-mineralised rod-shaped bacilli within the calculus matrix [185, 186] and has been observed in previous SEM studies of archaeological dental calculus [187]. Comparison of microparticle assemblages revealed no apparent differences in occurrence or diversity within dental calculus samples with pores compared to those without, nor any clear correlation between the occurrence of pores and the tendency of calculus samples to fragment during sampling. Nonetheless, the presence of these pores requires further archaeological testing and may have implications for microparticle diagenesis in calculus microenvironments (particularly of bacteria-susceptible starch granules) as well as the suitability of decontamination protocols for some samples, particularly chemical procedures operating under the assumption of an impermeable calculus matrix.

Within this study, the structural integrity of analysed calculus deposits varied, with 27 samples fully or partially disaggregating into smaller powder fragments (<0.5 mm) during sampling. To assess variation in the distribution of microparticles, the morphotype diversity of separately analysed fractions (powder and solid) from nine dental calculus samples was compared using a Jaccard similarity test (S6 Table in S1 File). Morphotype presence varied between corelated samples and the number of observed phytolith morphotypes was generally greater in powder fractions, however, this correlated with larger extract weights and may therefore reflect the size of the analysed fraction (S6 Table in S1 File). Interestingly, while overall recovery was low within this study, starch granules were only recovered from solid calculus samples and may indicate that less structurally robust calculus deposits were not conducive to the preservation of archaeological starch.

#### Microparticles recovered from dental calculus

No plant microparticles were recovered from the 15 negative controls run in conjunction with the archaeological samples. Plant microparticles were present in dental calculus from 34 of the 55 sampled individuals, occurring in 66.6% (n = 6), 63.6% (n = 14) and 58.3% (n = 14) of KDK1 Kerma, KDK1 Middle Neolithic and KDK21 Middle Neolithic samples, respectively (see S2 File for complete dental calculus microparticle results).

**Fig 5:**
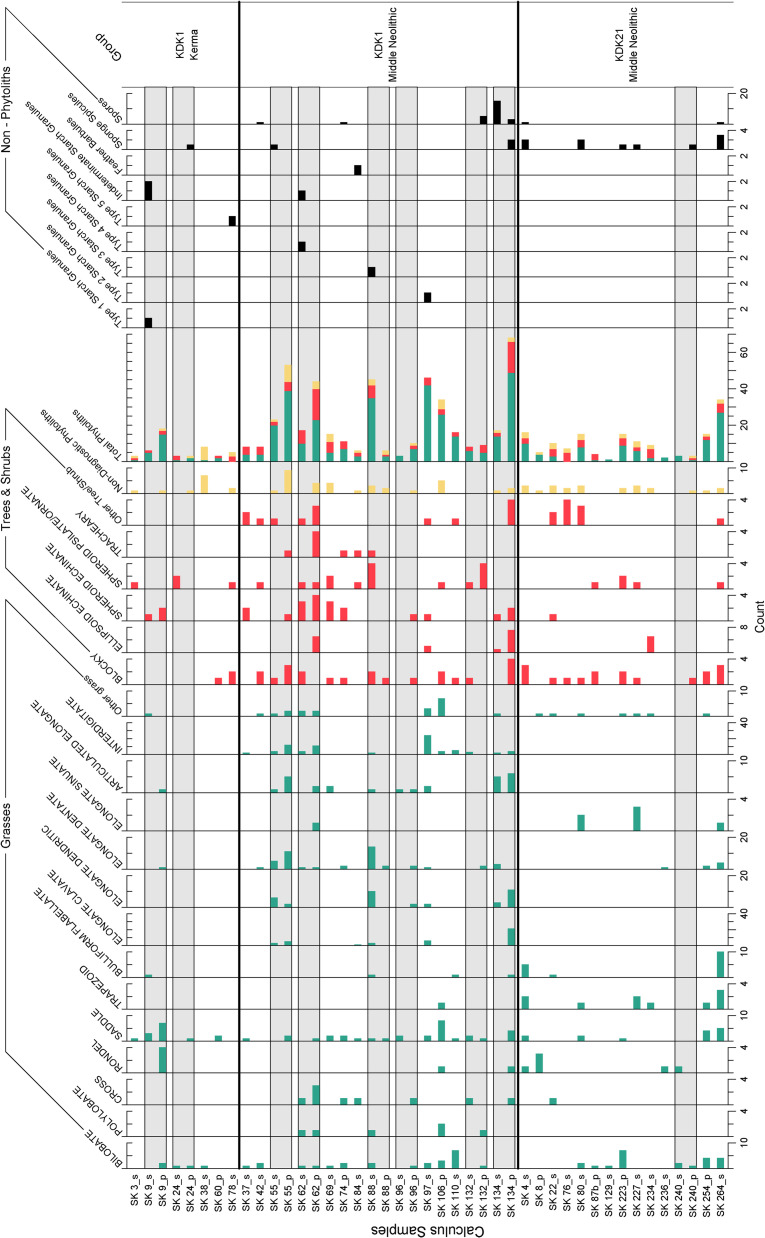
Summary of microparticles recovered from KDK1 and KDK21 dental calculus. Count data from 34 individuals with preserved microparticles. Samples are arranged according to site and chronological phase (‘Group’). Sample suffix _s indicates solid calculus, _p indicates powder calculus. Individual calculus samples with corresponding solid and powder fractions are indicated with shading for comparison of microparticle diversity/density.

**Fig 6:**
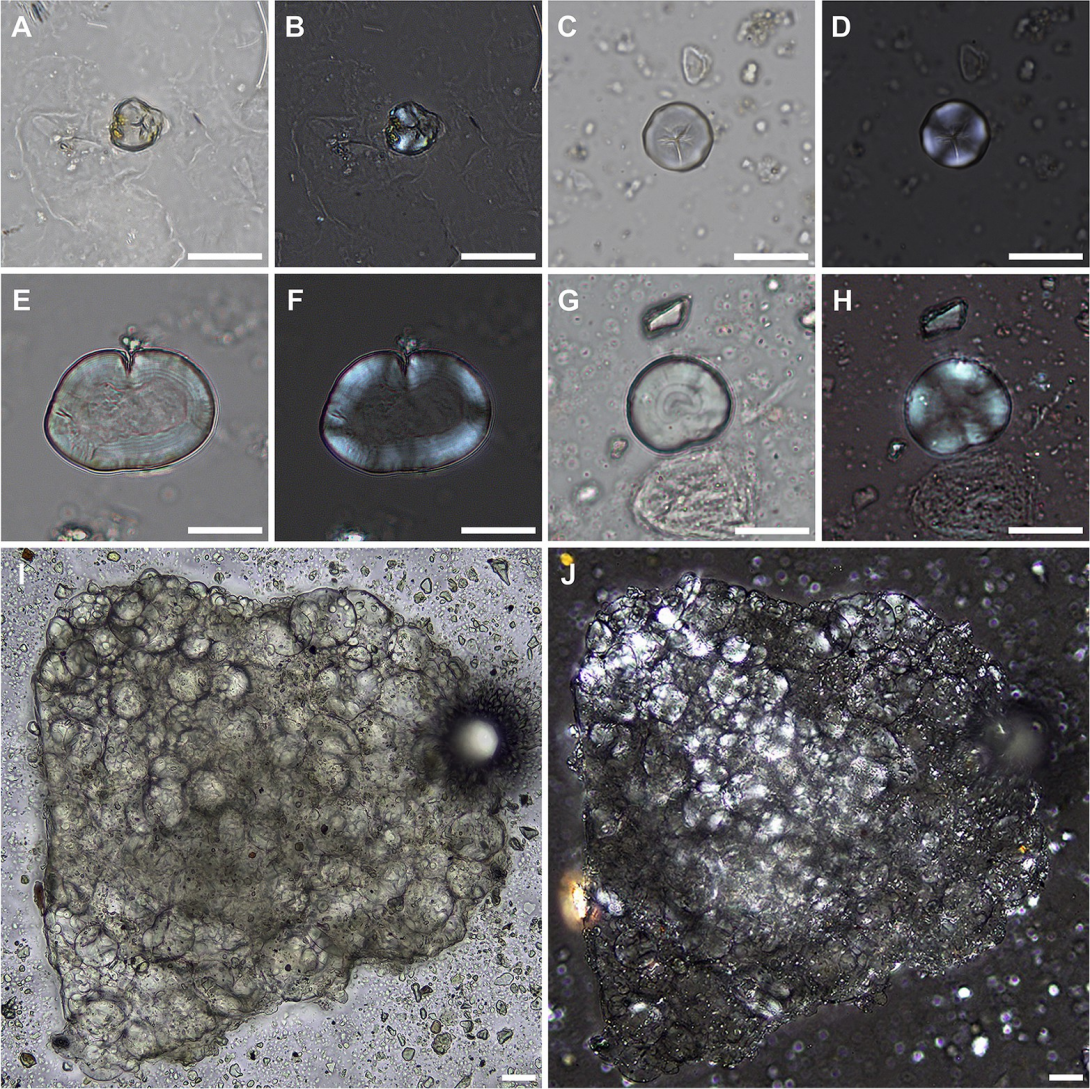
Diagnostic starch granules recovered from dental calculus of individuals from KDK1. (A–B) Type 1 native starch granule under plane (A) and cross-polarised light (B), individual SK 9 (Kerma); (C–D) Type 2 native starch granule under plane (C) and cross-polarised light (D), individual SK 97 (Middle Neolithic); (E–F) Type 3 modified starch granule under plane (E) and cross-polarised light (F), individual SK 88 (Middle Neolithic); (G–H) Type 4 native starch granule under plane (G) and cross-polarised light (H), individual SK 62 (Middle Neolithic); (I–J) cluster of Type 5 A-type and B-type starch granules under plane (G) and cross-polarised light (H), individual SK 78 (Kerma). Scale bar (20 μm) applies to all panels.

Starch granules occurred in 9.1% (n = 5) of dental calculus samples and were restricted to KDK1 Middle Neolithic and Kerma individuals (Fig 5). Five distinctive morphotypes (Table 2) were observed in this study. Type 1 (n = 1) was a subangular-polygonal, faceted granule with y-shaped fissure, slightly eccentric arms and a maximum width of 14.05 μm. This type matches published morphological and size criteria for Panicoid grasses (Fig 6A and 6B) [152, 154, 188, 189]. This type was represented by a single native starch granule recovered from the dental calculus of individual SK 9. Type 2 (n = 1) was a prismatic-polygonal granule with a centric stellate hilum and maximum width of 19.12 μm. This type also matched published characteristics of Panicoid grasses (Fig 6C and 6D) [148, 154] and was represented by a single granule in the calculus of individual SK 97. Type 3 (n = 1) was a modified cylindroid-ovate granule with lamellae visible on the outer margin and a maximum width of 46.70 μm. This type was represented by a single granule recovered from the calculus of individual SK 88 (Fig 6E and 6F). Evidence for swelling, a collapsed centre and loss of birefringence within this granule match published characteristics of early stages of cooking damage to Faboideae spp. [153]. Type 4 (n = 1) was globular-orbicular in shape, with a centric hilum, visible lamellae and a maximum width of 28.57 μm (Fig 6G and 6H). This morphotype is cautiously assigned to Fabaceae spp. based on published characteristics [148] and was represented by a single granule recovered from the calculus of individual SK 62.

**Table 2:** Summary of starch granule morphotypes preserved within the dental calculus of KDK1 individuals.

Type	Shape	Features	Max. W (μm)	Kerma		Middle Neolithic		
				SK 9	SK 78	SK 62	SK 88	SK 97
1	Subangular-polygonal	Faceted	14.05	1				
		y-shaped fissure						
		Slightly eccentric arms						
2	Prismatic-polygonal	Centric stellate hilum	19.12					1
3	Cylindroid-ovate	Lamellae visible on outer margin	46.70				1	
		Collapsed centre						
		Loss of birefringence						
4	Globular-orbicular	Centric hilum Visible lamellae	28.57			1		
5	Lenticular (A-type) and round/sub-oval (B-type)	Cluster with bimodal distribution	A-type = 51.37		1			
			B-type = <10					
Indeterminate				2*		1		

* partially gelatinised

The cluster recovered from Kerma period individual SK 78 was comprised of Type 5 starch granules with a bimodal distribution characteristic of Triticeae species [49, 151, 190, 191]. This bimodal distribution includes large lenticular (A-type) granules ranging in size from 21.89–51.37 μm and smaller round/sub-oval (B-type) granules <10 μm in size (Fig 6I and 6J). A-type starch granules within the cluster generally exhibited evidence of swelling and diminished birefringence indicative of food processing [153]. Three indeterminate starch granules displaying morphological modification were also recovered (Table 2).

**Fig 7:**
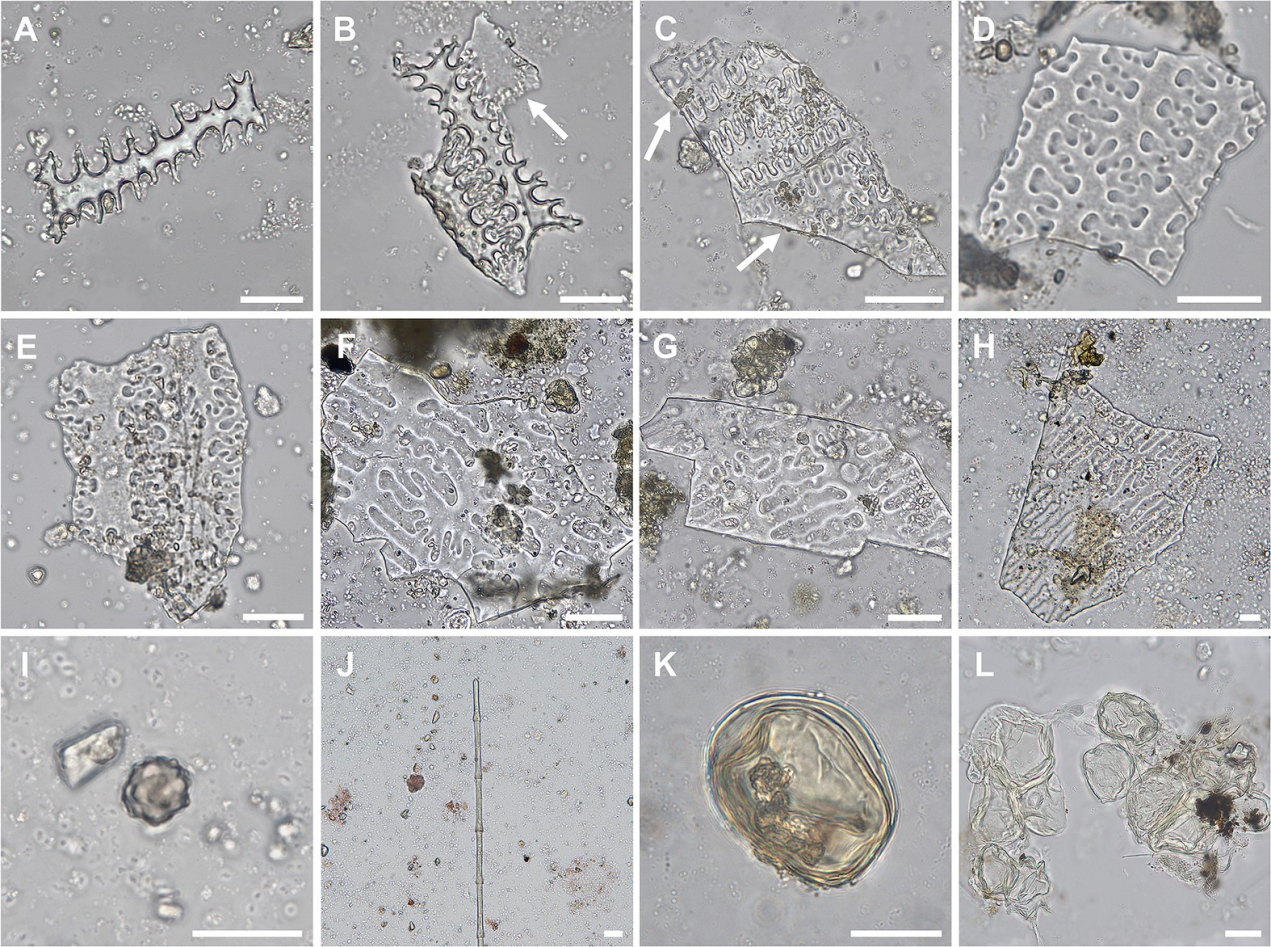
Key microparticle types recovered from dental calculus of KDK1 Middle Neolithic period individuals. (A) Elongate dendritic phytolith, individual SK 134; (B–C) Elongate dendritic phytoliths overlaying Interdigitate phytoliths, individuals SK 55 (B) and SK 97 (C); (D) Interdigitate phytoliths (*cf*. *Panicum* spp.), individual SK 55; (E) Interdigitate phytoliths (*cf*. *Panicum* spp.), individual SK 62; (F–H) Interdigitate phytoliths (*Echinochloa* spp.), individual SK 97; (I) Spheroid echinate phytolith, individual SK 134; (J) Feather barbule fragment, individual SK 84; (K–L) Fungal spores, individuals SK 134 (K) and SK 42 (L). White arrows in Panel B and C indicate Interdigitate phytolith layer attached to Elongate dendritic phytoliths. Scale bar (20 μm) applies to all panels.

While highly variable, microparticle assemblages from KDK21 and KDK1 dental calculus samples were primarily composed of phytoliths, with a total of 567 taxonomically identifiable phytoliths recovered from 34 of the 55 individuals (Fig 5). Phytoliths indicative of woody taxa (incl. Blocky tabular/facetate/granulate, Tracheary annulate, Spheroid echinate/psilate/ornate and Ellipsoid echinate [143, 192–194] were infrequently observed within the dental calculus assemblages (Fig 5). While the taxonomic specificity of these morphotypes is generally limited, Spheroid echinate phytoliths (n = 24) indicative of Arecaceae (palm species) [195–197] were present in dental calculus samples from all three studied groups (Fig 7I).

Diagnostic grass phytoliths had high ubiquity within the assemblage, occurring in 94% of dental calculus samples (32/34) that contained plant microparticles. These were also abundant, with 74.6% (n = 423) of diagnostic phytoliths attributable to grasses (Fig 5). A total of 148 short cell (Bilobate, Saddle, Trapezoid, Rondel, Cross) phytoliths were identified, with Bilobate and Saddle (n = 110) morphotypes associated with Panicoid and Chloridoid grasses present in all sample groups [198]. Diagnostic grass Elongate phytoliths with clavate, dendritic, dentate or sinuate projections along the lateral cell margins occurred individually (n = 123) and in articulated sheets (n = 27). These Elongate morphotypes were primarily recovered from KDK1 Middle Neolithic samples (Fig 7A). Observed in 13 samples, Elongate dentate phytoliths were the most common (n = 62), while Elongate dendritic phytoliths (n = 36, Fig 7A and 7B) were only present in the dental calculus of five KDK1 Middle Neolithic individuals. Generally considered diagnostic of Triticeae inflorescences in agricultural contexts [199–202], Elongate dendritic phytoliths also occur in wild African grass inflorescences, particularly within Panicoid grasses [203, 204]. Due to the limited recovery of articulated Elongate dendritic phytoliths from dental calculus and the potential non-Triticeae origin, we are precluded from confidently establishing any affinity to *Triticum* and *Hordeum* species through comparison with published morphometric parameters [200]. Furthermore, articulated Elongate dendritic phytoliths were frequently attached to Interdigitate phytolith fragments from Paniceae grasses (Fig 7B and 7C). These represent the preserved anatomical organisation of the outer (abaxial) layers of the fertile lemma and palea, with Elongate phytoliths underlying an Interdigitate phytolith layer in Paniceae grasses [144, 146]. The majority (80%, n = 4/5) of dental calculus samples with individual Elongate dendritic phytoliths also contained Interdigitate phytoliths, further suggesting association with wild Paniceae grasses rather than domesticated Triticeae cereals.

**Fig 8:**
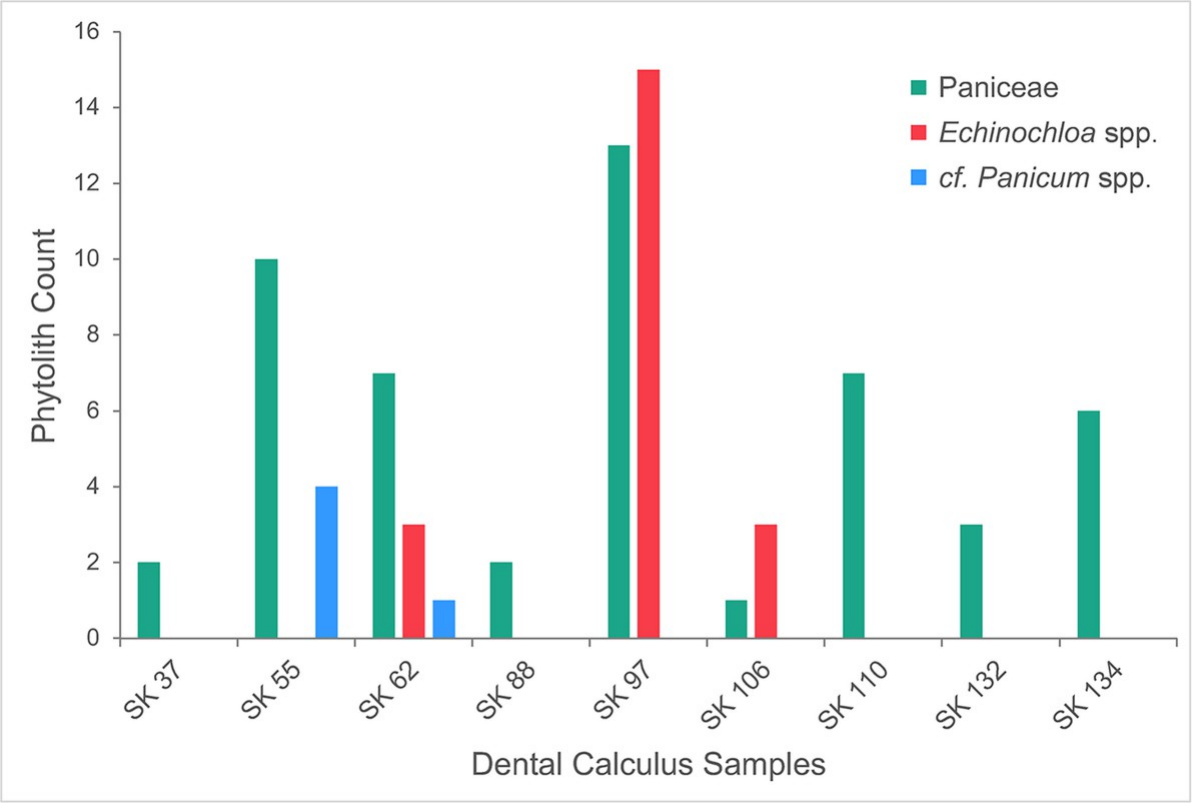
Interdigitate phytolith abundance and taxonomic classification. Taxonomic specificity of Interdigitate phytoliths present in dental calculus samples from KDK1 Middle Neolithic period individuals.

Fragments of Interdigitate phytoliths (n = 77) from the fertile floret (husk) of Paniceae grasses occurred in 64% (n = 9) of KDK1 Middle Neolithic individuals that contained diagnostic microparticles within the dental calculus matrix (Fig 8). Observation of key morphological traits (β-type undulations with attached Papillate cells and articulate terminal margins) enabled higher taxonomic classification of 21 Interdigitate phytoliths to *Echinochloa* spp. (Fig 7F and 7H) [38, 144, 146]. A further five Interdigitate phytoliths, matched published morphological attributes for *Panicum laetum* with η-type undulations, no Papillate cells and articulate/brachiate terminal margins [146, 205]. However, as these Interdigitate phytoliths were limited to a few fragments, these were tentatively classified as Paniceae *cf*. *Panicum* spp. (Fig 7D and 7E).

A single feather barbule fragment was recovered from KDK1 Middle Neolithic individual SK 84 (Fig 7J). Sponge spicules were observed across all sample groups. Fungal spores of various shapes and sizes characteristic of Glomeromycota (arbuscular mycorrhizal fungi) were observed in Middle Neolithic samples from KDK21 and KDK1 (Fig 7K and 7L) [206].

#### Phytolith assemblage variation between archaeological groups

**Fig 9:**
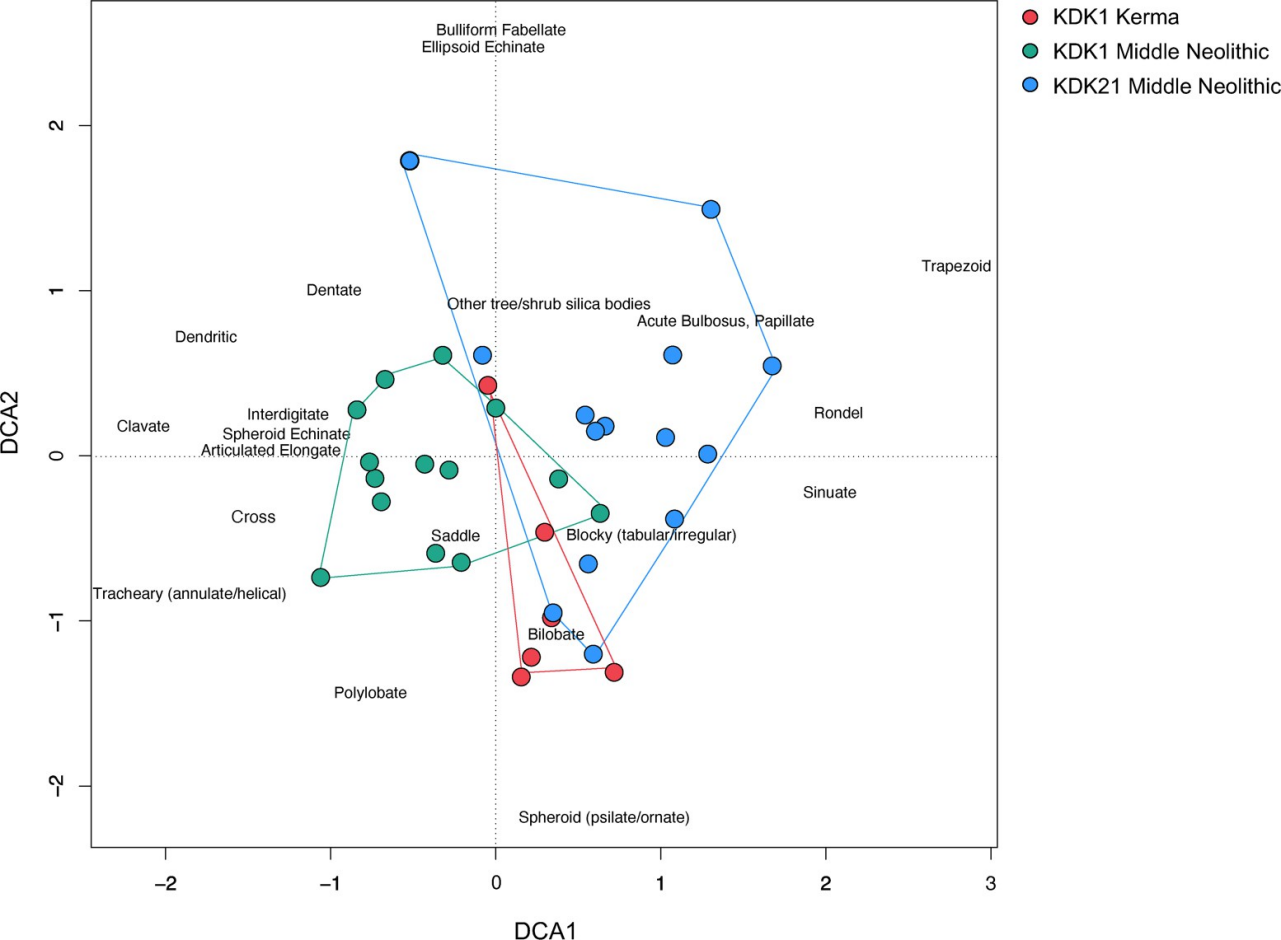
Detrended correspondence analysis (DCA) scatterplot of phytolith morphotype presence/absence data from dental calculus samples according to archaeological group. DCA scatterplot displaying 28.04% of the variance within the sample data, primary variation in the data is represented along axis 1, while secondary variation is represented along axis 2. The eigenvalues of axes 1 and 2 are 0.3280 and 0.2537, respectively. The total inertia is 2.075.

DCA of phytolith morphotype presence/absence in dental calculus between individuals indicate limited separation between the three archaeological groups (KDK1 Kerma, KDK1 Middle Neolithic, and KDK21 Middle Neolithic) (Fig 9). This observation is supported by the results of the ANOSIM, with an R value of 0.2862 indicating greater variation within each archaeological group than between groups (*p* = 0.001, refer to S7 Table in S1 File). This result is expected due to the co-occurrence of morphotypes within individual plants (multiplicity) and between species (redundancy), as well as the random nature of microparticle incorporation. However, there is some separation between archaeological groups along axis 1 with KDK1 Middle Neolithic samples clustering towards wild grass inflorescence morphotypes (Elongate clavate/dendritic and Interdigitate) observed exclusively within dental calculus samples from this archaeological group.

### Microparticles recovered from sediment samples

Only one starch granule was recovered from the seven sediments analysed; a Type 1 starch granule, maximum width 14.05 μm, in the sediment sample associated with KDK1 Middle Neolithic individual SK 132. No starch granules were observed in the corresponding dental calculus sample from this individual. Particulate charcoal fragments were observed in all sediment samples, and a single feather barbule fragment was observed in the sediment adhering to SK 236.

**Fig 10:**
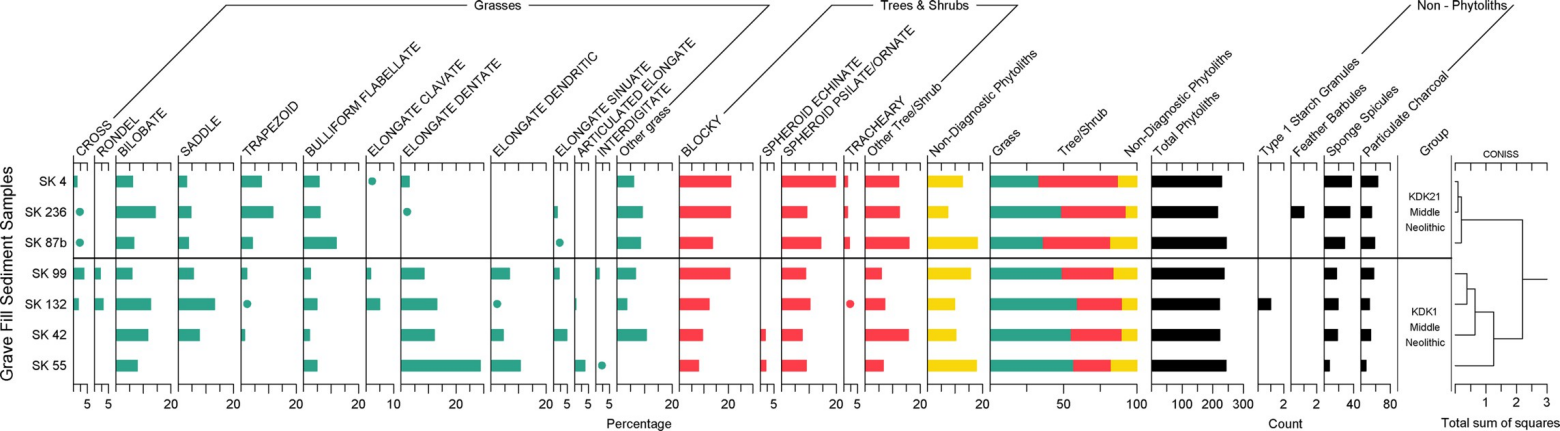
Summary of microparticle assemblages recovered from sediment samples from KDK1 and KDK21 Middle Neolithic grave fills. Phytolith morphotypes are presented as percentages, other microparticles are presented as counts. CONISS cluster analysis conducted on phytolith morphotype percentages only. Refer to S2 File for phytolith counts.

**Fig 11:**
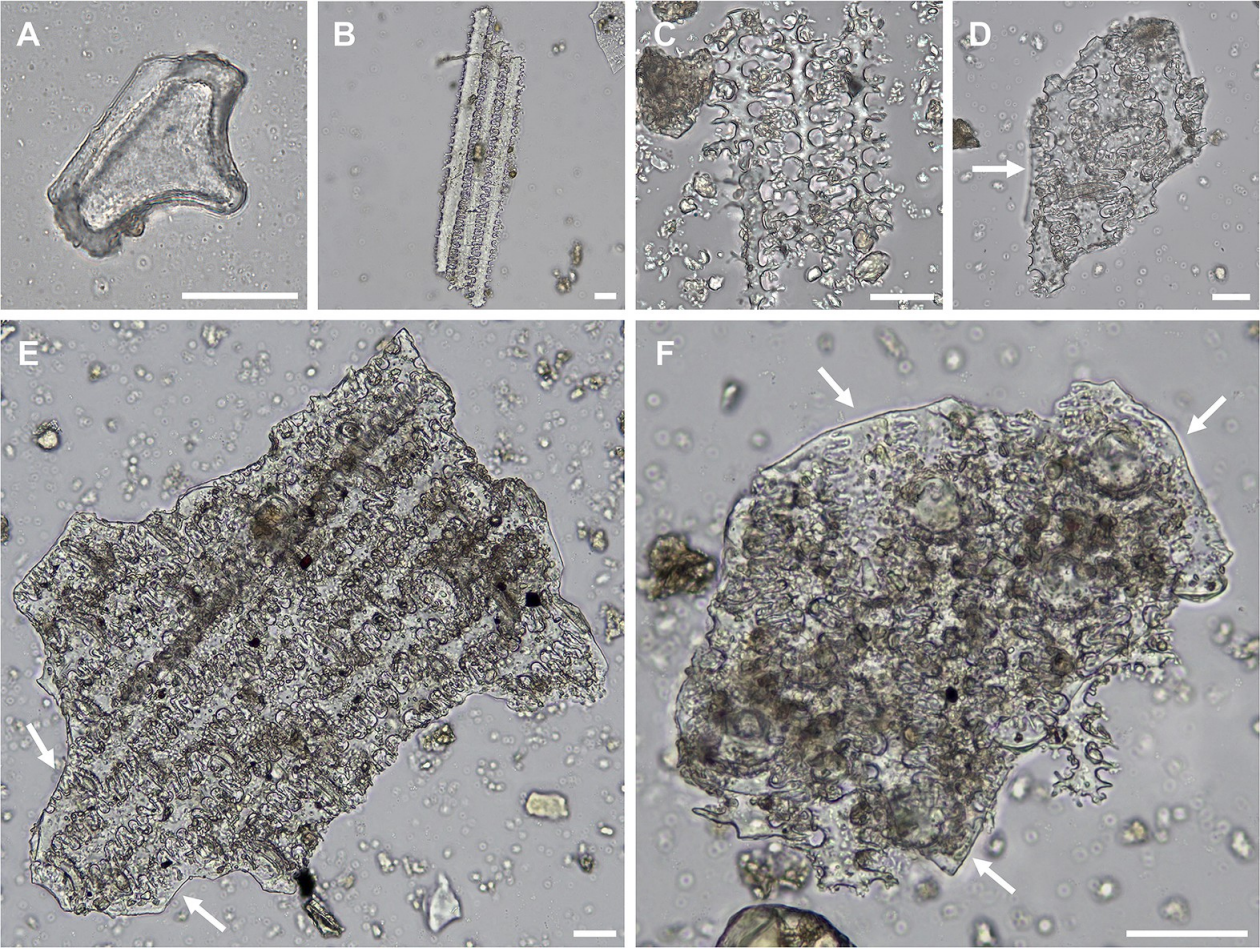
Examples of phytolith morphotypes extracted from sediment samples from KDK1 Middle Neolithic grave fills. (A) Bulliform flabellate phytolith extracted from sediment adhering to individual SK 42; (B–F) Phytoliths extracted from sediment adhering to individual SK 55, (B) Articulated Elongate dentate phytoliths; (C) Articulated Elongate dendritic phytoliths; (D) Articulated Elongate dendritic with attached Interdigitate layer; (E–F) Articulated Elongate dendritic with attached Interdigitate layer and Papillate cells. White arrows in Panel D, E and F indicate Interdigitate phytolith layer attached to Elongate dendritic phytoliths. Scale bar (20 μm) applies to all panels.

Phytoliths were present in all sediment samples associated with the KDK burials. Comparison of these assemblages supported by constrained incremental sum-of-squares (CONISS) analysis indicate a clear difference in morphotype composition between KDK1 and KDK21 samples (Fig 10). Elongate phytoliths were infrequent (<3.5%) in KDK21 sediments in comparison to KDK1 sediments (>21%). Grass morphotypes from KDK1 sediments suggest the presence of culm/leaf material (Elongate dentate (Fig 11C) and Bulliform flabellate (Fig 11D) phytoliths) and, to a lesser extent, chaff (Elongate dendritic and Interdigitate phytoliths) (Fig 10). Interdigitate phytoliths were present in low quantities in SK 55 and SK 99 sediments (1% n = 2 and 1.5% n = 3, respectively) in contrast to Elongate phytoliths (48% n = 96 and 22.5% n = 45, respectively). Sediment samples from three KDK1 graves (SK 42, 55 and 99) in particular, contained relatively high abundances of disarticulated Elongate dendritic phytoliths (21–47% relative to sum of grass short cells plus Elongate dendritic phytoliths).

### Fourier Transform Infrared Spectroscopy (FTIR) results

The full results of infrared indices obtained through FTIR-ATR analysis are provided in S10 Table in S1 File. All samples displayed classic enamel FTIR spectra, with no absorbance bands linked to the presence of secondary carbonate calcite observed (711 cm^-1^) (S3 Fig in S1 File). ANOVA and post-hoc Tukey pair-wise comparison showed no significant difference in A-site carbonation (S11, S12 Tables in S1 File), B-site carbonation (S13, S14 Tables in S1 File), dehydration/organic decay (WAMPI) (S15, S16 Tables in S1 File), crystallinity (PCI) (S17, S18 Tables in S1 File), or B-site/A-site carbonation ratio (BAI) (S19, S20 Tables in S1 File) between the archaeological human groups. Though a lack of comparative local modern samples limits direct assessment of diagenetic change, all archaeological groups present characteristic crystal-chemical signatures consistent with existing studies of enamel apatite (S4 Fig in S1 File) [161].

Loss of organic material and fossilisation of the apatite matrix is evident in most samples through low WAMPI, increased PCI and a relative increase in B-site versus A-site carbonate ions (low API and higher BAI). However, these changes in enamel crystal-chemical structure are expected within archaeological contexts and are not considered to have major impacts on stable carbon and oxygen isotopic measurements [161, 162]. While most samples fall within published ranges for archaeological enamel apatite [161, 162, 169–171], two Kerma period samples (T22 and SK 60) had elevated WAMPI and API values (S4 Fig in S1 File). This is particularly evident in fauna sample T22, which exceeds reported WAMPI values for modern fauna in East Africa (>0.3) [161]. As both the fauna (T22) and human (SK 60) samples originate from adolescent individuals, elevated WAMPI likely reflects higher organic and water content of immature enamel [161, 207].

### Stable isotope results

#### Stable carbon and oxygen isotope results from enamel

**Fig 12:**
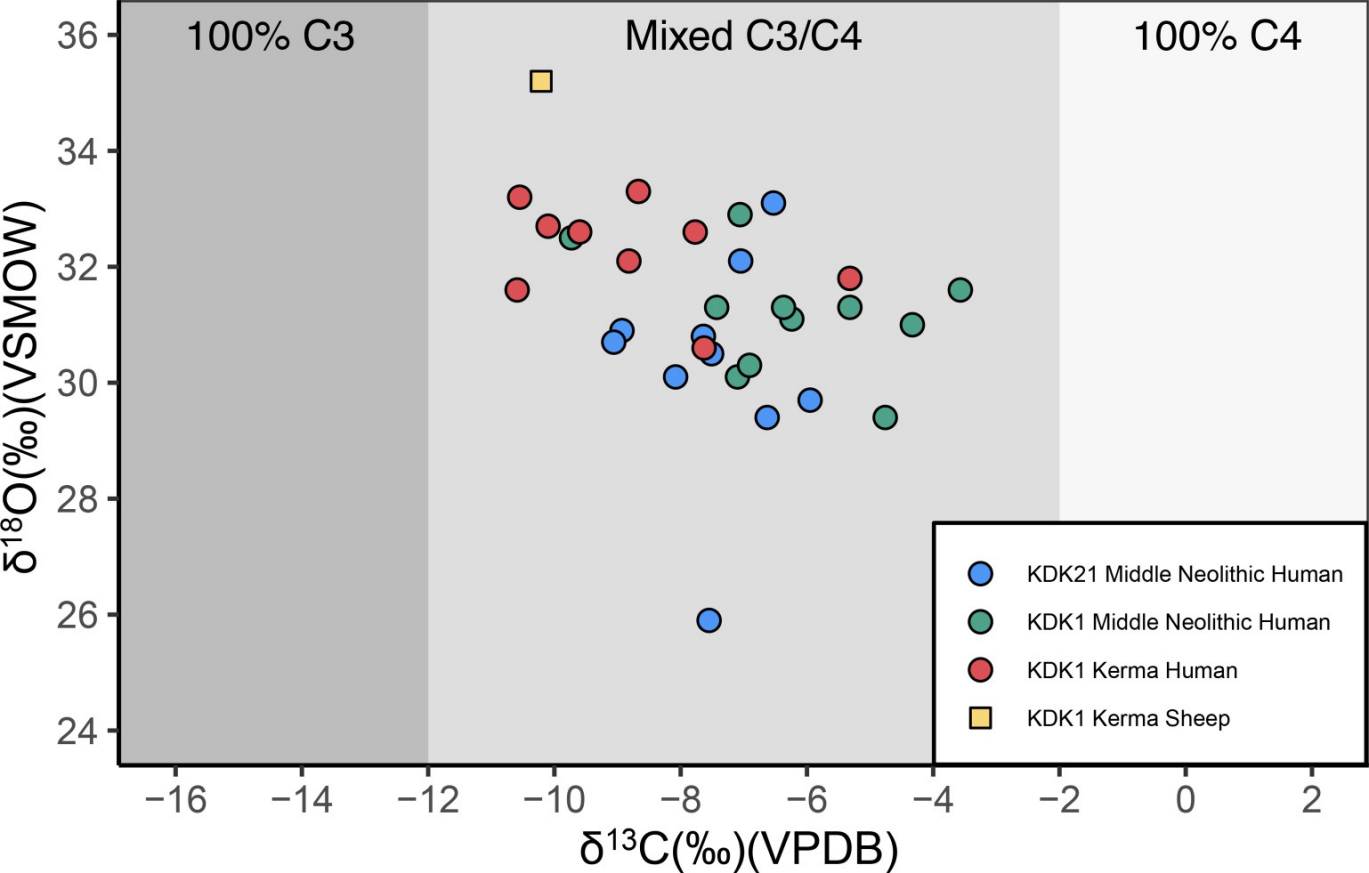
ẟ^13^C and ẟ^18^O measurements of human and faunal enamel from KDK21 and KDK1, Middle Neolithic and Kerma period. KDK1 Kerma faunal sample T22 is plotted in S5 Fig in S1 File. Shading indicates estimated carbonate ẟ^13^C for individuals consuming 100% C_3_, mixed C_3_/C_4_, and 100% C_4_ sources [208]. VPDB = Vienna Pee Dee Belemnite. VSMOW = Vienna Standard Mean Ocean Water.

The full dataset of ẟ^13^C and ẟ^18^O tooth enamel measurements of humans and fauna is shown in Fig 12 and S21 Table in S1 File.

ẟ^18^O carbonate values for humans ranged from 25.9‰ to 33.3‰ with a mean value of 31.2 ± 1.5‰. Mean ẟ^18^O carbonate values for KDK21 Middle Neolithic, KDK1 Middle Neolithic and KDK1 Kerma samples were 30.3 ± 1.8‰, 31.2 ± 0.9‰ and 32.3 ± 0.8‰, respectively. An ANOVA of ẟ^18^O by archaeological group followed by post-hoc Tukey pair-wise comparison reveals a significant distinction between KDK21 Middle Neolithic and KDK1 Kerma groups (F(2,27) = 5.084, *p* = <0.05, CI [-3.483, -0.434]) (S22, S23 Tables in S1 File). This distinction reflects both the differences in the aridity of the environments inhabited by these groups and the ẟ^18^O value of individual SK 237 from KDK21 (25.9‰). This outlier has a ẟ^18^O value greater than two standard deviations below the ẟ^18^O mean for KDK21 individuals. The ANOVA was thus rerun with this outlier removed to confirm the distinction between KDK21 Middle Neolithic and KDK1 Kerma individuals (F(2,26) = 5.209, *p* = <0.05, CI [-2.652, -0.281]) (S24, S25 Tables in S1 File).

ẟ^18^O values from the Kerma period faunal samples T9 and T22 were 35.2‰ and 45.2‰, respectively. Higher oxygen values within *Ovis aries* specimens are consistent with semi-obligate drinkers obtaining higher proportions of body water from plants that are ^18^O enriched from preferential evapo-transpiration of ^16^O [111, 112]. However, sample T22, an ~2.5- to 3-month-old *Ovis aries* [117], was excluded from direct comparison with human groups as both the FTIR and the ẟ^18^O carbonate values are well above typical reported values and likely reflect immature enamel (S21 Table and S5 Fig in S1 File).

ẟ^13^C carbonate values for humans ranged from -3.5‰ to -10.5‰ with a mean value of -7.4 ± 1.7‰. Mean ẟ^13^C carbonate values for KDK21 Middle Neolithic, KDK1 Middle Neolithic and KDK1 Kerma samples were -7.5 ± 1.0‰, -6.3 ± 1.7‰ and -8.8 ± 0.8‰, respectively. An ANOVA of carbonate ẟ^13^C by group, followed by post-hoc Tukey pair-wise comparison reveals a significant distinction between KDK1 Middle Neolithic and Kerma groups (F(2,27) = 6.944, *p* = <0.05, CI [0.844, 4.210]) (S26, S27 Tables in S1 File). In contrast to the other individuals from the same archaeological group, Kerma period individual SK 87a and Middle Neolithic period individual SK 106 had ẟ^13^C values of -5.3‰ and -9.7‰, respectively (Fig 12). The Kerma period *Ovis aries* sample T9, had a ẟ^13^C carbonate value of -10.2‰ consistent with a mixed C_3_/C_4_ diet.

#### Stable carbon and nitrogen isotope results from collagen

**Fig 13:**
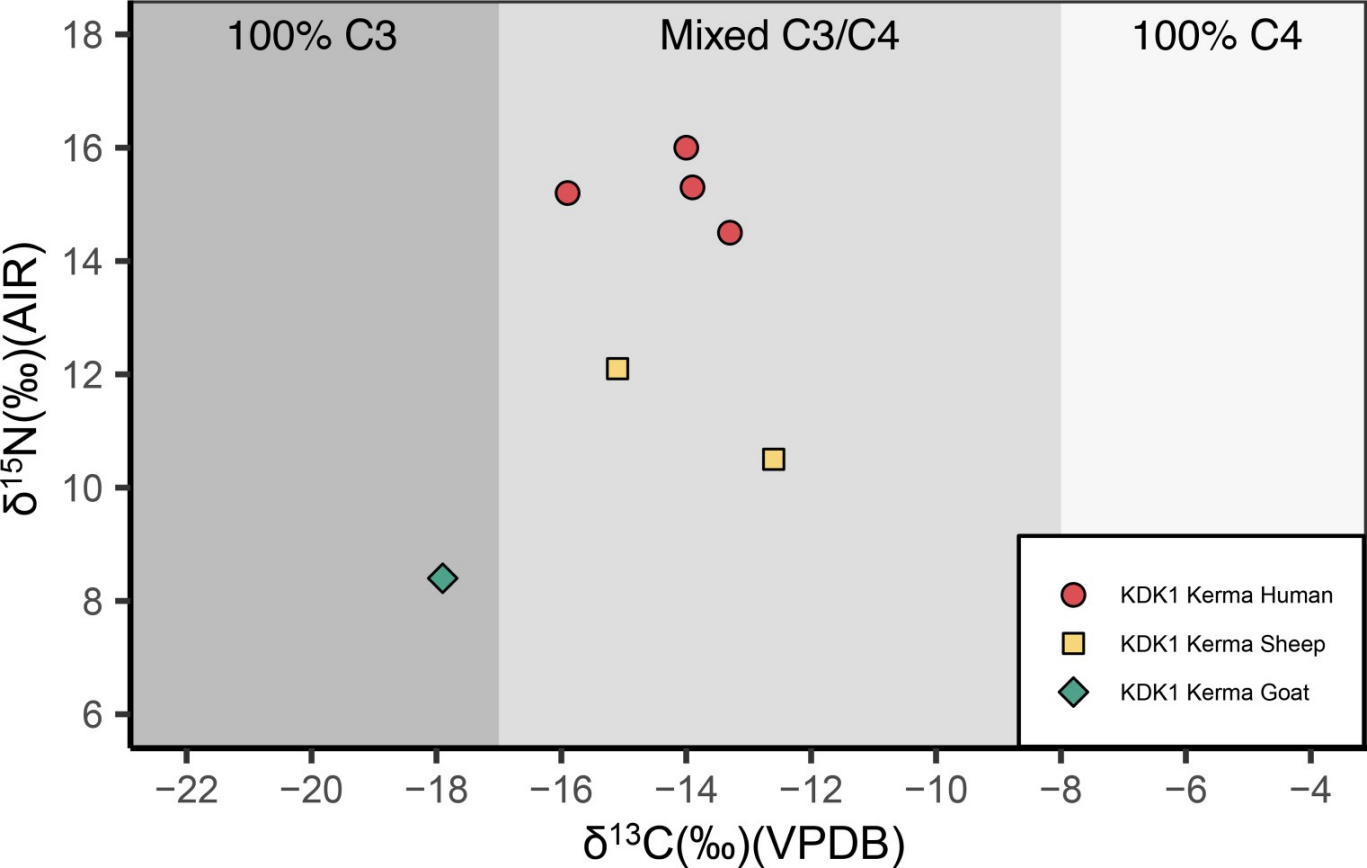
ẟ^13^C and ẟ^15^N measurements of human tooth dentine and faunal bone collagen from KDK1, Kerma period. Shading indicates estimated collagen ẟ^13^C for individuals consuming 100% C_3_, mixed C_3_/C_4_, and 100% C_4_ sources [208]. VPDB = Vienna Pee Dee Belemnite. AIR = Ambient Inhalable Reservoir.

The full dataset of ẟ^13^C and ẟ^15^N KDK1 Kerma period human tooth dentine and faunal bone collagen measurements is shown in Fig 13 and S5 Table in S1 File.

Kerma period human ẟ^13^C collagen values, ranging from -13.3‰ to -15.9‰ with a mean value of -14.2 ± 1.1‰, match corresponding bioapatite measurements, which indicate a mixed C_3_/C_4_ diet. ẟ^13^C collagen values for two *Ovis aries* samples, of -15.1‰ and -12.6‰, also indicate a mixed C_3_/C_4_ diet as expected for grazers [101]. In contrast, the -17.9‰ ẟ^13^C collagen value for the *Capra hircus* sample is consistent with an expected C_3_ dominated signature for browsers [209]. As the C/N ratios for all samples, excepting *Ovis aries* sample T95/2, fall within the liberal upper limit (3.4–3.6) proposed by Guiry and Szpak [181], we might potentially expect a slight carbon isotopic shift of ~1‰ in the collagen samples. Observed ẟ^13^C values for the fauna are, however, consistent with the expected stable isotopic composition for each taxon (greater C_3_ input in browsers/greater C_4_ input in grazers) [209]. Similarly, direct comparison of ẟ^13^C_coll_ and ẟ^13^C_enamel_ values for the Kerma period individuals indicates stable isotopic spacing of 5.1 ± 0.4‰. This broadly correlates with published human ẟ^13^C carb-coll variation between enamel and dentine [50, 99]. Regardless, a slight shift in ẟ^13^C in the collagen samples would not alter the interpretation of the broad dietary patterns observed in this study.

In contrast to the ẟ^13^C values, higher C/N ratios have a negligible influence on ẟ^15^N values [181]. ẟ^15^N collagen values for humans ranged from 14.5‰ to 16.0‰ with a mean of 15.2 ± 0.6‰. The ẟ^15^N collagen value for the *Capra hircus* sample was 8.4‰, while the *Ovis aries* samples had values of 10.5‰ and 12.1‰. These faunal values are generally consistent with existing regional studies demonstrating nitrogen enrichment derived from consumption of plants within arid environments [54, 55, 210, 211]. However, the nitrogen value of 12.1‰ from T5, a young *Ovis aries* specimen, exceeds published values for archaeological caprines within the region and likely reflects a weaning effect. Comparison between KDK1 Kerma humans and fauna ẟ^15^N values, excluding T5, reveal a mean trophic level enrichment within humans of 5.8‰.

## Discussion and conclusion

### Dietary signatures at KDK21 and KDK1

While the comparatively infrequent recovery of phytoliths and total absence of starch granules within the dental calculus of Middle Neolithic individuals from KDK21 limit interpretation, ẟ^13^C carbonate values indicate that they consumed a mixed C_3_/C_4_ diet. In contrast, ẟ^13^C carbonate values for KDK1 Middle Neolithic individuals are more varied, with C_4_ resources forming the dominant contribution to the diet of 5 individuals (Fig 12). These results are supported by the microparticle assemblages from Middle Neolithic individuals at KDK1 which include starch granules from Panicoid grasses and Fabaceae species in addition to phytoliths from wild grass inflorescences. Furthermore, the dental calculus of nine Middle Neolithic individuals from KDK1 contained Interdigitate phytoliths, including fragments diagnostic of *Echinochloa* spp. and potentially *Panicum laetum*. These C_4_ wetland adapted grasses would have occurred in dense stands along the channel margins and in seasonally inundated areas of the alluvial plain with grains maturing following the retreat of Nile floodwaters in the dry season.

Existing studies have demonstrated that Interdigitate phytoliths occur within the fertile floret (lemma and palea closely adhering to the grain) of C_4_ Paniceae grasses [144, 146, 147, 212]. The presence of these Interdigitate fragments within dental calculus likely reflects the contamination of grains during dehusking or the consumption of these small grains without dehusking as has been observed ethnographically in northern Africa [213]. A recent study of the dentition of Middle Neolithic individuals from KDK21 and KDK1 indicated high frequencies of caries and dental calculus [78]. While these traits are often associated with an agricultural diet rich in fermentable carbohydrates [116, 214], the dietary data reported in this study suggest that the high prevalence of these dental pathologies is likely attributable to the significant dietary contribution of wild grasses also rich in fermentable carbohydrates.

It is worth noting that grass inflorescence phytoliths also occur in the four sediment samples from KDK1 graves (Fig 10). Previous studies have established that a high abundance of Elongate dendritic phytoliths in sediments is indicative of anthropogenic accumulations from the collection and processing of grass seed inflorescences [154, 203]. These phytolith types have been observed in sediments underlying a Middle Neolithic individual at R12 [37] and five Kerma *Ancien* individuals at H29 [215]. Although the high abundance of Elongate dendritic phytoliths in three sediment samples from KDK1 (SK 42, SK 55 and SK 99) resemble the reported Triticeae phytolith assemblage from grave 46 at the Middle Neolithic site of R12 [37, 38], the prevalence of Bilobate and Saddle short cell phytoliths and scarcity of Rondel phytoliths in the KDK1 sediments is more suggestive of a signal composed of Chloridoideae and Panicoideae wild grasses [203, 204].

While there is overlap in phytolith morphotypes between dental calculus and sediment samples from KDK1, we are confident that the dental calculus signature from the nine Middle Neolithic individuals reflects human consumption of wild grasses. In contrast to the dental calculus assemblages, the composition of the sediments indicates a mixed input characterised by culm/leaf material (Elongate dentate and Bulliform flabellate phytoliths) and, to a lesser extent, chaff/inflorescence material (Elongate dendritic and occasional Interdigitate phytoliths) which likely reflect the deliberate deposition of grasses at the time of burial (Fig 10). This low abundance of Interdigitate phytoliths (n = 5) in the sediments relative to other grass phytoliths may reflect the limited presence of fertile florets.

**Fig 14:**
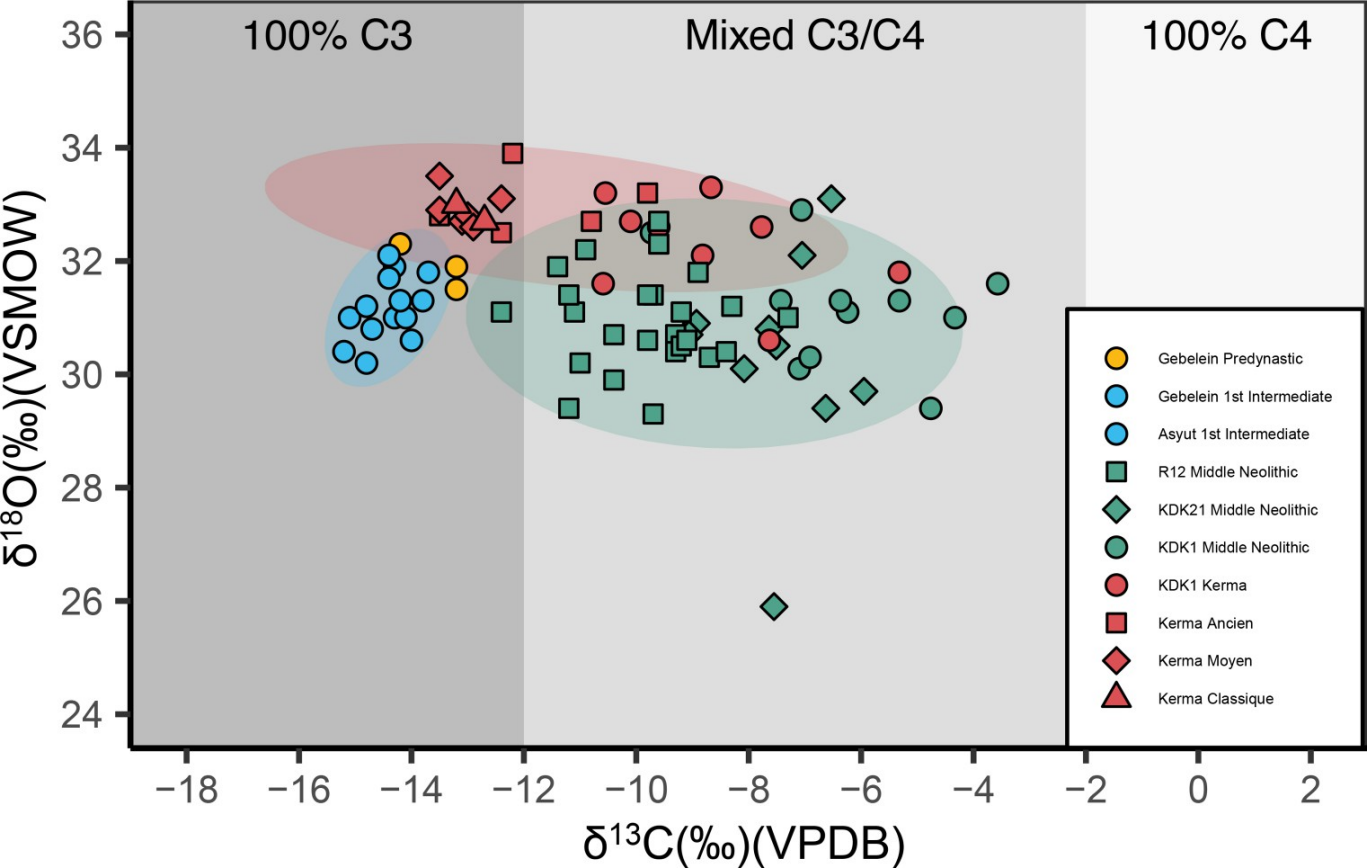
ẟ^13^C and ẟ^18^O carbonate measurements of humans from this study and relevant Egyptian and Upper Nubian Nile Valley sites prior to New Kingdom conquest 1500 BCE. Refer to S28 Table in S1 File for mean values. Gebelein and Asyut [53], R12 [50], Kerma *Ancien*, *Moyen* and *Classique* refer to Eastern Cemetery individuals [52]. Shading indicates estimated carbonate ẟ^13^C for individuals consuming 100% C_3_, mixed C_3_/C_4_, and 100% C_4_ sources [208]. VPDB = Vienna Pee Dee Belemnite. VSMOW = Vienna Standard Mean Ocean Water.

**Fig 15:**
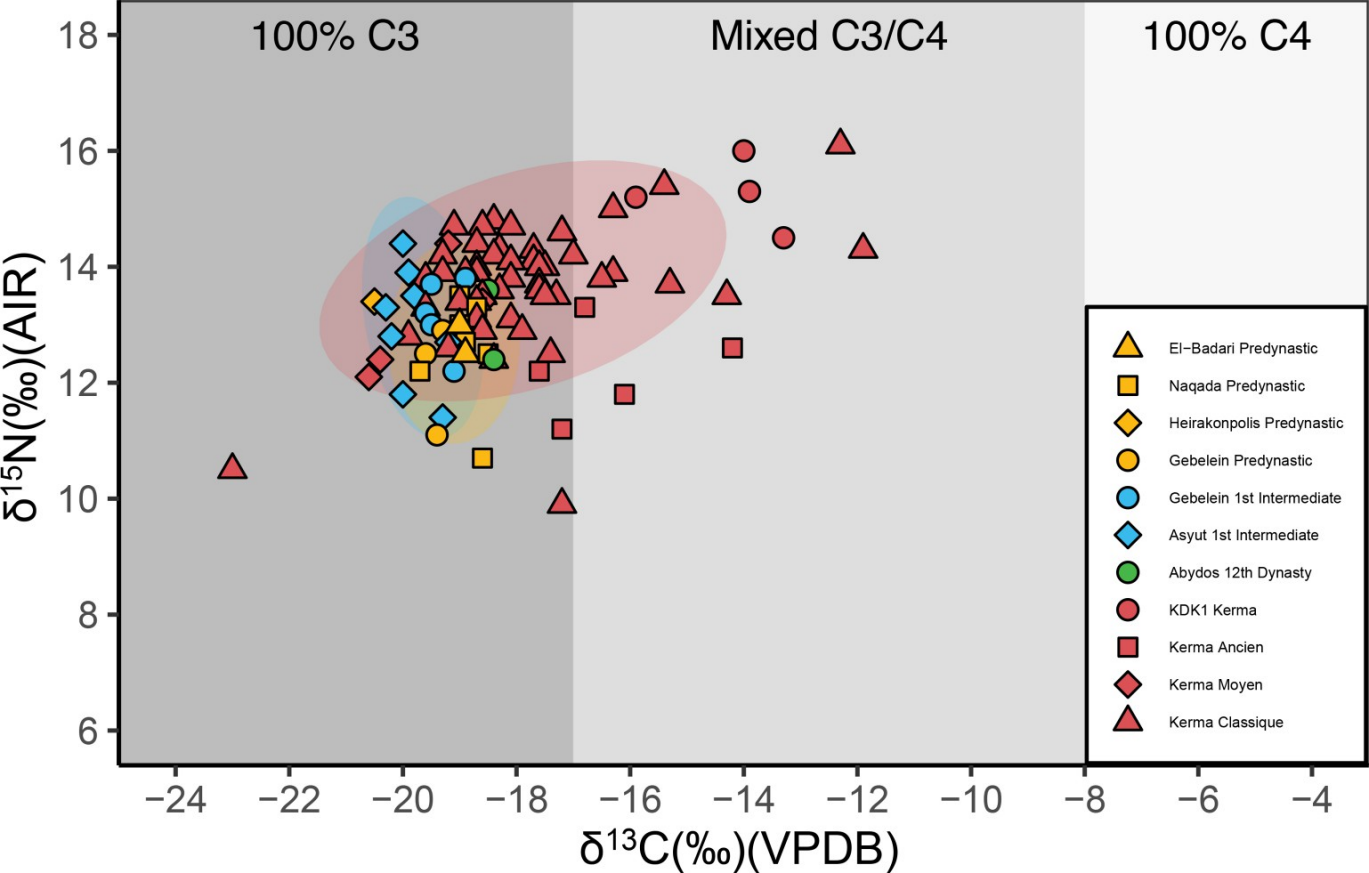
ẟ^13^C and ẟ^15^N collagen measurements of humans from this study and relevant Egyptian and Upper Nubian Nile Valley sites prior to New Kingdom conquest 1500 BCE. Refer to S28 Table in S1 File for mean values. Abydos, El-Badari, Naqada and Hierakonpolis [55], Gebelein and Asyut [53], Kerma *Ancien*, *Moyen* and *Classique* refer to Eastern Cemetery individuals [52, 54]. Shading indicates estimated collagen ẟ^13^C for individuals consuming 100% C_3_, mixed C_3_/C_4_, and 100% C_4_ sources [208]. VPDB = Vienna Pee Dee Belemnite. AIR = Ambient Inhalable Reservoir.

Marked differences in the microparticle assemblages of Kerma period individuals at KDK1 likely reflect changing foodways, with lower recovery of plant microparticles potentially suggesting the diminished use of wild plants, particularly wild grasses. This coincides with evidence for an elaboration of storage facilities at the end of the Kerma *Ancien* period [216, 217]. New radiocarbon dates reported here for Kerma period individuals and fauna at KDK1 confirm that this population dates to the end of the Kerma *Ancien* and beginnings of the Kerma *Moyen* period (Fig 3). The recovered cluster of Triticeae starch from the dental calculus of Kerma period individual SK 78 provides direct evidence for the consumption of domesticated cereals and is in line with a greater contribution of C_3_-derived resources reflected in the stable isotope signatures (mean carbonate value of -8.8 ± 0.8‰, mean collagen value of -14.3 ± 1.1‰) relative to Middle Neolithic individuals (mean carbonate value of -7.49 ± 1.0‰ for KDK21 and -6.26 ± 1.7‰ for KDK1). However, the stable carbon isotopic values from these KDK1 Kerma period individuals are slightly enriched relative to reported *Ancien* (mean carbonate value of -11.7 ± 1.5‰, mean collagen value of -16.3 ± 1.3‰) and *Moyen* (mean carbonate value of -13.1 ± 0.4‰, mean collagen value of -19.7 ± 0.9‰) values from the Eastern Cemetery at Kerma (Figs 14 and 15 and S28 Table in S1 File) [50, 52].

Similarly, ẟ^15^N values for Kerma period individuals at KDK1 (mean value of 15.2 ± 0.6‰) are also higher when compared to the urban population (*Ancien* mean value of 12.2 ± 0.7‰, *Moyen* mean value of 13.1 ± 1.0‰) from the Eastern Cemetery at Kerma (Fig 15) [52]. As elevated ẟ^15^N values and corresponding ẟ^13^C human collagen values at KDK1 track the *Ovis aries* values from the site (Fig 13), higher ẟ^15^N in these humans likely reflects the significant dietary contribution of protein derived from isotopically enriched semi-obligate drinkers consuming nitrogen enriched grasses on the margins of the alluvial plain, which is supported by the elevated ẟ^18^O value in the fauna [105, 106]. ẟ^15^N enrichment does slightly exceed the accepted range of 3–5‰ per trophic level [102, 103], which may indicate the additional inclusion of some freshwater resources in the diets of these Kerma period individuals at KDK1. Although fish bones have not been observed in funerary assemblages, Kerma settlements are typically located directly adjacent to palaeochannels [32, 165, 218] with fish remains recovered from habitation contexts at both Gism el-Arba (GAH1) and Kerma [219]. Interestingly, previously published ẟ^15^N and ẟ^13^C values obtained from a hair sample from KDK1 Kerma individual SK 68 (ẟ^13^C -17.0‰, ẟ^15^N 12.0‰ [56]) were greater than two standard deviations below the means reported in this study. Stable isotopic values in bone collagen and hair keratin represent different periods of an individual’s life with differences in amino acid compositions between these proteins resulting in bone collagen values generally being slightly enriched (ẟ^13^C +1.4‰, ẟ^15^N +0.9‰) relative to hair keratin [220]. Although confirmation requires further palaeopathological analysis of this individual, reduced isotopic values in the hair of SK 68 may also reflect diminished protein intake or trauma towards the end of this individual’s life [221, 222].

ẟ^18^O values for Kadruka individuals are consistent with existing studies of Middle Nile Valley populations [223]. However, KDK21 Middle Neolithic individual SK 237 has an ẟ^18^O value resembling individuals of non-Nile Valley origin [224], which suggests this individual may have originated from outside the Nile Valley. Higher ẟ^18^O values in Kerma period individuals is linked to greater evaporation and evapo-transpiration consistent with increasing regional aridification [110, 111].

### Reconsidering evidence for the uptake of agriculture in the Northern Dongola Reach of Upper Nubia

The domesticated barley remains found underlying several Middle Neolithic individuals at KDK1 have variously been interpreted as trade commodities or indicators for an early establishment of flood recession farming in Upper Nubia [31, 39, 45, 59]. However, direct dietary evidence obtained through the analysis of 22 Middle Neolithic individuals associated with these grave goods provides no clear indication for routine consumption of domesticated cereals. Rather, the combined stable isotopic and microbotanical results demonstrate that these individuals were consuming wild plants including Fabaceae spp. and wetland adapted wild C_4_ grasses that would have been locally abundant along the margins of the Wadi el-Khowi and low-lying areas of the alluvial plain following the annual retreat of floodwaters. Attesting to the maintenance of subsistence flexibility and dietary breadth through the use of wild resources, these results are in line with existing archaeobotanical evidence reported from Neolithic sites in other regions of northeastern Africa such as Farafra and Nabta Playa in the Western Desert [1, 6, 225, 226], and later Khartoum Neolithic settlements and cemeteries in Central Sudan [37, 38, 57, 58, 227–231].

Although the lack of direct dietary evidence does not necessarily rule out small-scale cultivation of cereals by Neolithic populations at KDK1, these new results necessitate a critical reconsideration of the economic significance of the barley spikes and chaff included in the funerary assemblages. While researchers have argued that the presence of chaff and articulated spikes at KDK1 confirm local production [59], hulled cereals such as barley were often transported and stored in spikelet form to reduce spoilage [232, 233]. Furthermore, the infrequent presence of cereals as grave goods associated with emergent elite hierarchies at KDK1 and lack of evidence for routine consumption suggests value associations linked to initial access to exotic trade commodities [234, 235], for which the grave good assemblages at KDK1 and other Neolithic cemeteries in Upper Nubia provide ample evidence [31, 36, 77, 236, 237].

The Neolithic evidence for Southwest Asian cereals in Upper Nubia remains limited to a small number of funerary contexts at R12 (1 analysed sample, [37]) and KDK1 (unspecified number of graves, [39]) with the dietary signature reported here suggesting that wild plants rather than crops formed a key dietary component at KDK1. These new multidisciplinary findings, obtained from a representative sample size with robust contamination controls applied, are supported by recently published microbotanical evidence for wild *Cyperus esculentus* and *Vigna luteola* in pottery sediments at the Middle Neolithic site of KDK23 [63]. Furthermore, microbotanical investigations of grave sediments at the site of H29 indicate that wild grasses remained culturally important during the Kerma *Ancien* period (2500–2050 BCE) [215].

While the archaeological visibility and investigation of Neolithic habitations on the alluvial plain of Upper Nubia is limited, there is no evidence for storage facilities similar to those observed at contemporaneous sites in the Fayum and Nile Delta [16, 17, 21, 236, 238]. Rather, Neolithic habitations on the alluvial plain suggest periodic seasonal occupations, with discrete cultural layers interspersed with Nile silts [27, 238]. Combined with the Middle Neolithic dietary signatures at KDK1, which indicate the consumption of hydrophytic wild grasses that would have been abundant following the retreat of the Nile floodwaters, evidence for seasonal Neolithic occupations may indicate that these forager-herder populations maintained dietary flexibility through horizontal transhumance, moving seasonally between the alluvial plain and hinterland areas situated close to wadis [70, 238–240].

**Fig 16:**
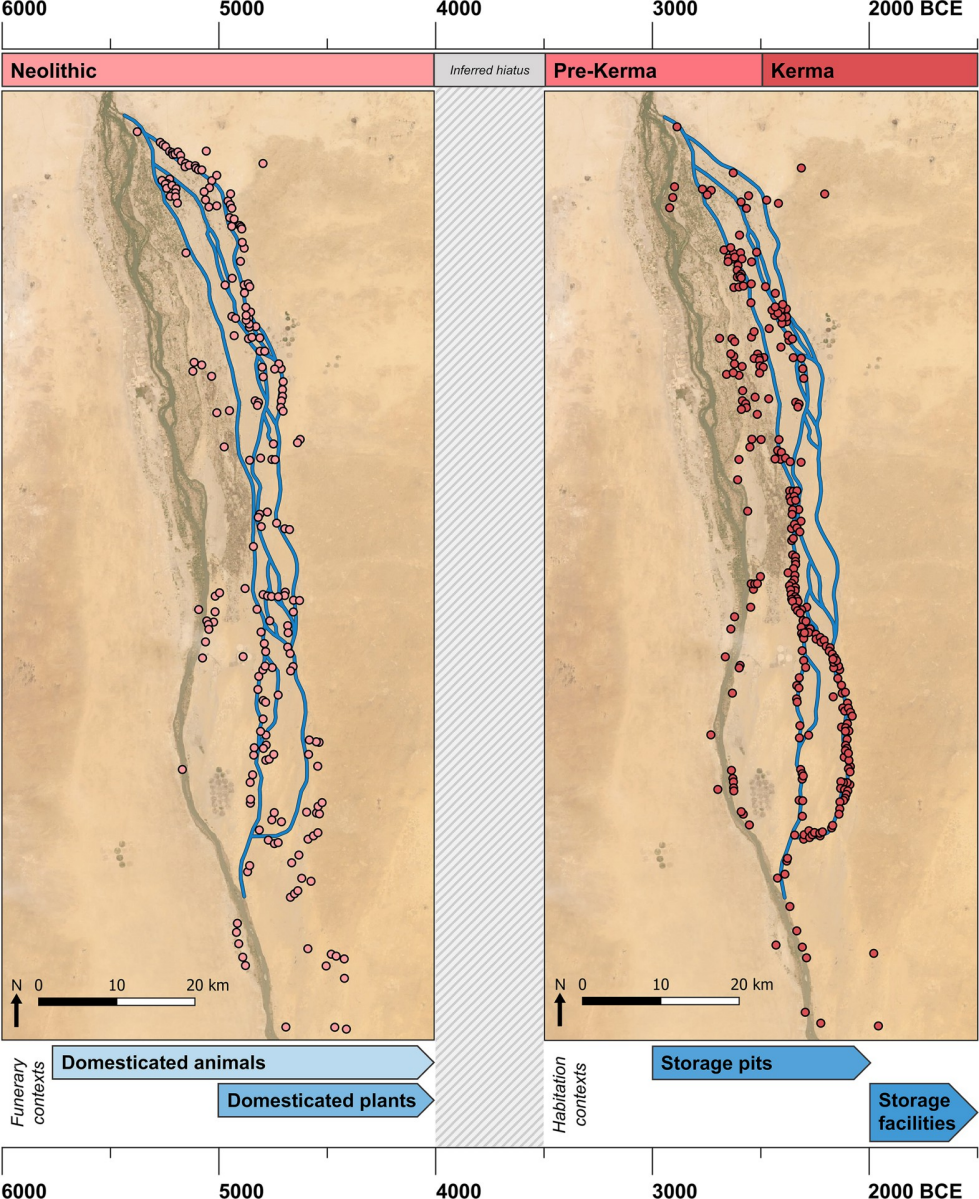
Changes in site distribution on the alluvial plain and corresponding evidence for food production economies. A) Distribution of Neolithic period sites on the alluvial plain; B) Distribution of pre-Kerma and Kerma period sites on the alluvial plain, note increased clustering along palaeochannels. Inferred hiatus between 4000–3500 BCE based on the limited evidence for a Late Neolithic presence on the alluvial plain which suggests a significant population reduction during this period. Site locations georeferenced using existing data [27, 32, 40]. World Imagery base map sourced from: Esri, Maxar, Earthstar Geophysics, and the GIS User Community (ArcGIS Pro Licence 3.0).

Situated within a regional context, the results of this study suggest a later economic transition in Upper Nubia postdating the suggested Late Neolithic hiatus on the alluvial plain (Fig 16 and Table 3) [25, 27, 45]. Although recently published dates from KDK5A and KDK5B indicate some occupation continuity during the early 4^th^ millennium BCE [75], there is clearly a significant reduction in the number of sites on the alluvial plain at the end of the 5^th^ millennium BCE [27, 241, 242]. The limited archaeological evidence for Late Neolithic groups on the alluvial plain during the early- to mid 4^th^ millennium BCE correlates with the progressive drying up of the desert wadis combined with an episode of reduced Nile flow and floodplain contraction [27, 29, 163]. While archaeological evidence indicates Predynastic populations in Egypt responded with increased agricultural production (Table 3), with stable isotopic studies indicating a heavily C_3_ focused diet (Figs 14 and 15), Neolithic groups located on the alluvial plain of Upper Nubia seem to have adopted a more flexible response to this period of environmental stress [27]. It is during this period that large-scale facilities linked with the storage of domesticated crops first appear in A-Group contexts in Lower Nubia [22, 243, 244]. Signalling a shift in subsistence orientation, these facilities are subsequently observed in Upper Nubia following the appearance of the pre-Kerma culture (3500–2500 BCE) and increased site density on the alluvial plain [43, 45, 238, 245–247].

**Table 3:** Summary of early archaeological evidence for Southwest Asian cereals in the Nile Valley prior to New Kingdom conquest 1500 BCE.

Context	Site	Chronological Period and Age	Further information
**The Delta and Lower Egypt**			
Storage pits	Fayum	Neolithic (4550–4350 BCE)	Kom K has storage pits containing emmer and barley [16, 18, 19]
Habitation	Merimde	Neolithic (4500 BCE)	Storage pits containing emmer wheat and barley [17, 21]
Habitation	El-Omari	Neolithic (4100 BCE)	Storage pits containing emmer wheat and barley [20]
**Upper Egypt**			
Storage pits	Mostagedda	Predynastic (4352–4243 BCE)	Directly dated emmer wheat and barley seeds, precise archaeological context unclear [5]
Habitation	El-Abadiya 2	Predynastic (3960–3640 BCE)	Threshing remains from emmer wheat and barley [248]
Habitation	Hierakonpolis	Predynastic (3800–3200 BCE)	Large scale crop processing facilities [23, 24]
Habitation	Adaïma	Predynastic (3500–2900 BCE)	Charred and desiccated emmer wheat and barley remains preserved in *pisé* (pit linings), recovered from funerary and habitation contexts [249, 250]
Habitation	Nag el-Qarmila	Predynastic (3800–3600 BCE)	Storage pits—inferred use [251]
**Lower Nubia**			
Storage pits	Khor Daoud	A-Group (3800–3100 BCE)	578 storage pits—inferred use [243]
Funerary	Qustul	A-Group (3800–3100 BCE)	Storage pits—inferred use [244]
Funerary	Serra West	A-Group (3800–3100 BCE)	Storage pits—inferred use [252]
Habitation	Afyeh	A-Group (3800–3100 BCE)	Storage pits containing carbonised emmer wheat and barley [22]
Funerary	Toska West	C-Group (2400–1550 BCE)	Barley in graves [62]
Habitation	Buhen	Old Kingdom (2686–2181 BCE)	Barley, context unclear [62]
**Upper Nubia**			
Funerary	R12	Middle Neolithic (Period 1A, 4920–4450 BCE)	Triticeae phytoliths identified in one sediment sample from grave 46 (superior) [37, 38]. Outlier C^14^ date (5311–5066 BCE) directly obtained from phytoliths possibly inaccurate (refer to text)
Funerary	KDK1	Middle Neolithic (4544–3967 BCE)	Barley glumes and spikes underlying unknown number of Neolithic graves. ‘Vegetal matter’ and/or glumes were observed in 19 Middle Neolithic graves, unclear how many of these deposits contained domesticated cereals [31, 39, 78]
Storage pits	8-B-52A (Sai Island)	Pre-Kerma (3600–2600 BCE)	134 storage pits, emmer wheat and barley present in some pits. Barley directly dated to 2800–2600 BCE [43–45]
Habitation	Site 1, Eastern Cemetery	Pre-Kerma (3127–2877 BCE)	>285 storage pits—inferred use [238, 241, 245]
Habitation	Sedeinga	Pre-Kerma (3341–3083 BCE)	Storage pits—inferred use [246]
Habitation	ARD002 (Arduan Island)	Pre-Kerma (3200–2500 BCE)	Storage pits—inferred use [247, 253]
Funerary	H29	Kerma *Ancien* (2500–2050 BCE)	Potential Triticeae phytoliths identified in one sediment sample from grave 158 [215]
Funerary	O16	Kerma *Ancien* (2500–2050 BCE)	Barley fragments burial C3 skeleton 20 (stomach contents?) [254]
Funerary	P37	Kerma *Moyen* (2050–1750 BCE)	Charred emmer wheat and barley in seven graves [254]
Funerary	SKC1 (Sai Island)	Kerma *Moyen* (2050–1750 BCE)	Charred barley in Tombs 53 and 54 [255, 256]
Funerary	Eastern Cemetery	Kerma (2500–1450 BCE)	Cereal offerings in tombs [217]
Habitation	Kerma	Kerma *Ancien* (2500–2050 BCE)	Circular storage pits reminiscent of pre-Kerma examples, some above ground silos [183, 217]
		Kerma *Moyen* (2050–1750 BCE)	Above ground silos (diameter ranging from 0.6m – 4.8m), some semi-subterranean mudbrick storage pits, total of 50 silos that reduce in number closer to the Kerma *Classique* period [217]. Bakery installations with ovens and bread moulds [183, 245]
		Kerma *Classique* (1750–1450 BCE)	Storage centralisation—13 large storage silos (diameter 3m – 7m) [183, 217]. Bakery installations with ovens and bread moulds [183, 245]
Habitation	GAH1 (Gism el-Arba)	Kerma *Moyen* (2050–1750 BCE)	Semi-subterranean mudbrick storage pits and raised mudbrick silos—inferred use [217, 257]
		Kerma *Classique* (1750–1450 BCE)	Raised storage buildings with stone foundations—inferred use [217, 257]
Habitation	GAH2 (Gism el-Arba)	Kerma *Moyen* (2050–1750 BCE)	Semi-subterranean mudbrick storage pits and one raised storage building with stone foundations—inferred use [217, 257]
		Kerma *Classique* (1750–1450 BCE)	Emmer wheat and barley remains. 21 raised storage buildings with stone foundations [217, 257; Marchi pers comm]
Habitation	GAH10 (Gism el-Arba)	Kerma *Moyen* (2050–1750 BCE)	Semi-subterranean mudbrick storage pits—inferred use [217, 257]
		Kerma *Classique* (1750–1450 BCE)	Raised storage buildings with stone foundations—inferred use [217, 257]
Habitation	P4	Kerma *Classique* (1750–1450 BCE)	Raised storage building with stone foundations—inferred use [32, 258]
Habitation	M13	Kerma *Classique* (1750–1450 BCE)	Numerous raised storage buildings with stone foundations—inferred use [32, 258]
Funerary	Ukma	Kerma *Classique* (1750–1450 BCE)	Emmer wheat and barley found in graves [259]
**Central Sudan**			
Dental calculus	Ghaba	Neolithic (4750–3650 BCE)	Singular large starch granules attributed to Triticeae found in seven dental calculus samples [37]. Outlier C^14^ date from grave 233 (5620–5480 BCE) directly obtained from phytoliths possibly inaccurate (refer to text)
Dental calculus	Al Khiday	Neolithic (4604–4239 BCE)	Possible presence of Triticeae starch granules with bimodal distribution in one Neolithic dental calculus sample [57]

Egyptian sites represent early finds with secure early identifications and ^14^C determinations. Sites with storage pits that do not have direct botanical evidence are not necessarily indicators of cereal storage. Select examples of raised storage ‘granaries’ from the Kerma *Classique* period in Upper Nubia listed, refer to Welsby 2001 for additional sites. Refer to Fig 1 for site locations.

At KDK1, direct dietary evidence for the consumption of domesticated cereals was only associated with one individual from the Kerma *Ancien/Moyen* period, corresponding with a shift in isotopic values towards a more C_3_-derived diet. This is consistent with existing evidence in Upper Nubia for increased agricultural production based on flood recession farming of hulled barley and emmer wheat on the alluvial plain at the beginning of the Kerma *Moyen* period (2050 BCE). This shift follows the 4.2 ka arid event that was characterised locally by significantly reduced Nile flow and is often associated with the collapse of the Old Kingdom in Egypt [29, 163–165, 260]. Although direct archaeobotanical evidence remains limited during the Kerma period, the Kerma *Moyen* transition is characterised by an isotopic shift to primarily C_3_ comprised diets at the Eastern Cemetery (Figs 14 and 15) [50, 52] and increased occurrence of cereal remains in graves (Table 3). These indicators correlate with broader socio-cultural markers clearly indicating a change in economic intensity including greatly increased site density along the Alfreda and Seleim palaeochannels (Fig 16) [29, 32, 165], a shift to mudbrick building techniques [183], development of storage facilities at non-urban sites on the alluvial plain [32, 217, 245, 258], the appearance of communal storage facilities at Kerma [183, 217], and greater social stratification at the Eastern Cemetery [184].

The new evidence presented in this paper provides an integrated multidisciplinary analysis of diachronic changes in plant utilisation in Upper Nubia combined with a critical reappraisal of the economic significance of existing evidence for agriculture. The integration of dietary isotopes with microbotanical analyses of human dental calculus indicates that Middle Neolithic individuals at KDK1 were consuming hydrophytic wild grasses and other riverine plant resources combined with animal derived products [56]. Providing no clear indication for routine consumption of domesticated cereals, these results necessitate a critical reconsideration of the significance of domesticated cereals recovered from the funerary assemblages of these individuals. Although previous studies have suggested that the Neolithic evidence for domesticated cereals supports an early agricultural transition as part of a Neolithic ‘package’, presence within funerary contexts and absence within corresponding dietary signatures is more reflective of high value associations of trade items [4, 234, 235]. The results of this study correlate with existing studies of early low-level food production systems [261–263], demonstrating that Middle Neolithic populations on the alluvial plain maintained dietary flexibility through the use of domesticated animal products and readily available wild resources. While Neolithic populations in Upper Nubia had access to domesticated cereals and small-scale cultivation may have formed a limited contribution to local diets, current evidence suggests a later economic transition linked to broader socio-cultural changes occurring amidst increasingly arid environmental conditions.

## Supporting information

S1 File Supporting information. (DOCX)

S2 File Results from dental calculus and sediment samples. (XLSX)
