# Supplementary material for: Ecological flexibility and adaptation to past climate change in the Middle Nile Valley: A multiproxy investigation of dietary shifts between the Neolithic and Kerma periods at Kadruka 1 and Kadruka 21
Source: PLoS One. 2023 Feb 2;18(2):e0280347. doi: 10.1371/journal.pone.0280347 (PMC9894462; doi:10.1371/journal.pone.0280347)
Supplement: S1 File — (DOCX) [file pone.0280347.s001.docx]

S1 File. SUPPORTING INFORMATION

# Ecological flexibility and adaptation to past climate change in the Middle Nile Valley: A multiproxy investigation of dietary shifts between the Neolithic and Kerma periods at Kadruka 1 and Kadruka 21

Charles Le Moyne^1,2,*^, Patrick Roberts^3,2,1^, Quan Hua^4,1^, Madeleine Bleasdale^5,2^, Jocelyne Desideri^6^, Nicole Boivin^1,2^ Alison Crowther^1,2^

^1^ School of Social Science, The University of Queensland, Michie Building, Saint Lucia, QLD, Australia

^2^ Department of Archaeology, Max Planck Institute for Geoanthropology, Jena, Germany

^3^ isoTROPIC Research Group, Max Planck Institute for Geoanthropology, Jena, Germany

^4^ Australian Nuclear Science and Technology Organisation (ANSTO), Locked Bag Kirrawee DC, NSW, Australia

^5^ Department of Archaeology, University of York, Kings Manor, Exhibition Square, York

^6^ Laboratory of African Archaeology and Anthropology, Section of Biology, University of Geneva, Geneva, Switzerland

* Corresponding author:

Email address: [lemoyne@shh.mpg.de](mailto:lemoyne@shh.mpg.de) (CLM)

**Supporting Information:**

**Supporting Note 1. Biological characteristics of individuals selected for this study.**

**Supporting Figures:**

**S1 Fig. Reference Interdigitate phytoliths.**

**S2 Fig. Reference starch granule morphotypes.**

**S3 Fig. Averaged FTIR spectra for enamel samples from each group.**

**S4 Fig. Box plots of the infrared indices (API, BPI, WAMPI, PCI, BAI).**

**S5 Fig. ẟ^13^C and ẟ^18^O measurements of human and faunal samples from KDK1 and KDK21.**

**Supporting Tables:**

**S1 Table. List of individuals with dental calculus selected for this study.**

**S2 Table. List of fauna samples from KDK1 Kerma period graves selected for this study, classified by Chaix [12].**

**S3 Table. Protocol for phytolith extraction from sediments through microwave digestion and heavy liquid flotation.**

**S4 Table. Protocol for starch granule extraction from sediments through deflocculation, oxidisation and heavy liquid flotation.**

**S5 Table. Isotopic and AMS radiocarbon results on collagen extracted from faunal bone and human tooth dentine, published radiocarbon dates for KDK1 and KDK21, and modelled ages.**

**S6 Table. Difference in phytolith assemblage composition between dental calculus fractions.**

**S7 Table. Results of Analysis of Similarity by sample group, based on the presence or absence of diagnostic phytolith morphotypes in dental calculus.**

**S8 Table. Description of the FTIR spectrum of enamel bioapatite.**

**S9 Table. Description of the FTIR indices of enamel bioapatite.**

**S10 Table. Table of the infrared indexes (WAMPI, API, BPI, PCI, BAI) for sample groups subjected to Fourier-Transform Infrared Spectroscopy diagenesis study (as per Roche et al. [10]).**

**S11 Table. Results of ANOVA for API of diagenesis study groups.**

**S12 Table. Results of post-hoc Tukey pair-wise comparison for API of diagenesis study groups.**

**S13 Table. Results of ANOVA for BPI of diagenesis study groups.**

**S14 Table. Results of post-hoc Tukey pair-wise comparison for BPI of diagenesis study groups.**

**S15 Table. Results of ANOVA for WAMPI of diagenesis study groups.**

**S16 Table. Results of post-hoc Tukey pair-wise comparison for WAMPI of diagenesis study groups.**

**S17 Table. Results of ANOVA for PCI of diagenesis study groups.**

**S18 Table. Results of post-hoc Tukey pair-wise comparison for PCI of diagenesis study groups.**

**S19 Table. Results of ANOVA for BAI of diagenesis study groups.**

**S20 Table. Results of post-hoc Tukey pair-wise comparison for BAI of diagenesis study groups.**

**S21 Table. Bulk δ^13^C and δ^18^O measurements of enamel human and faunal specimens from KDK1 and KDK 21.**

**S22 Table. Results of ANOVA for δ^18^O (VSMOW) by group.**

**S23 Table. Results of post-hoc Tukey pair-wise comparison for δ^18^O (VSMOW) by group.**

**S24 Table. Results of ANOVA for δ^18^O (VSMOW) by group with outlier (SK 237) removed.**

**S25 Table. Results of post-hoc Tukey pair-wise comparison for δ^18^O (VSMOW) by group with outlier (SK 237) removed.**

**S26 Table. Results of ANOVA for δ^13^C (VPDB) by group.**

**S27 Table. Results of post-hoc Tukey pair-wise comparison for δ^13^C (VPDB) by Site/Phase.**

**S28 Table. Existing human stable isotopic (ẟ^13^C, ẟ^18^O and ẟ^15^N) data from relevant Egyptian and Upper Nubian Nile Valley sites prior to New Kingdom conquest 1500 BCE.**

**Supporting Note 1. Biological characteristics of individuals selected for this study.**

The skeletons excavated from KDK21 and KDK1 curated at the University of Geneva were originally analysed by C. Simon [1]. The Middle Neolithic individuals from KDK21 and KDK1 were reanalysed by E. Maines [2] and colleagues currently working on Neolithic material in the Kadruka concession [3]. Kerma period individuals included in this study were reanalysed by J. Desideri.

Skeletal elements are generally poorly preserved, particularly those assigned to Middle Neolithic individuals. The biological sex and age at death of individuals included in this study are listed below (Table 1). Depending on the preservation of the individuals, morphological features of the pelvis bones were used to determine biological sex [4, 5]. In cases where the craniofacial bones were well preserved, observations of the cranial morphology were used as a secondary criterion. Determination of age-at-death was primarily based on the observation of the auricular surface [6, 7]. Immature individuals were aged using dental eruption [8, 9].


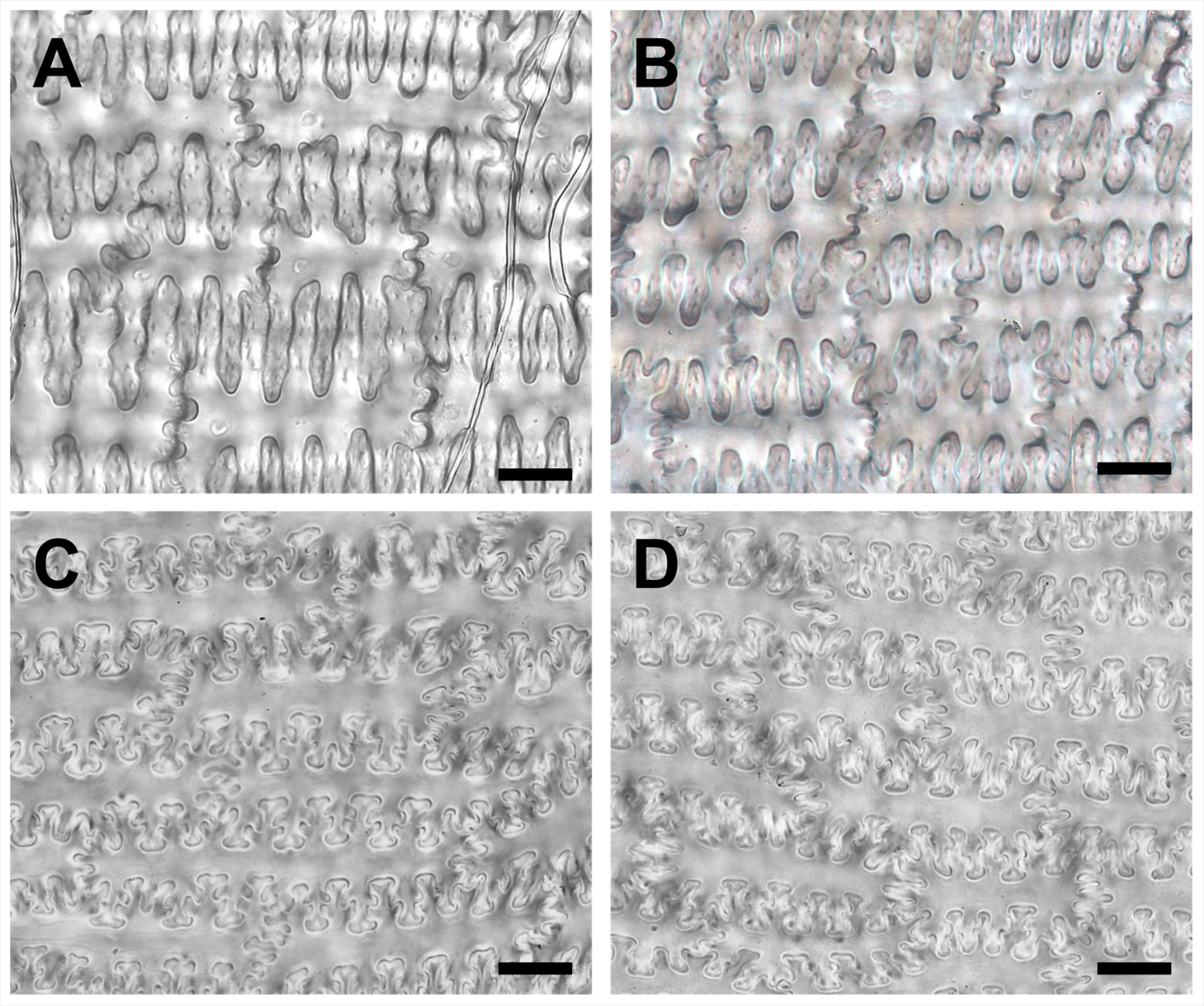


**S1 Fig. Reference Interdigitate phytoliths.**

(A–B) Reference *Echinochloa* spp. (*Echinochloa colona*) Interdigitate phytolith layer from fertile lemma with β-type undulations, attached Papillate cells and articulate terminal margins; (C–D) Reference *Panicum* spp. (*Panicum laetum*) Interdigitate phytolith layer from fertile lemma with η-type undulations, no Papillate cells and articulate/brachiate terminal margins. Scale bar (20 µm) applies to all panels.


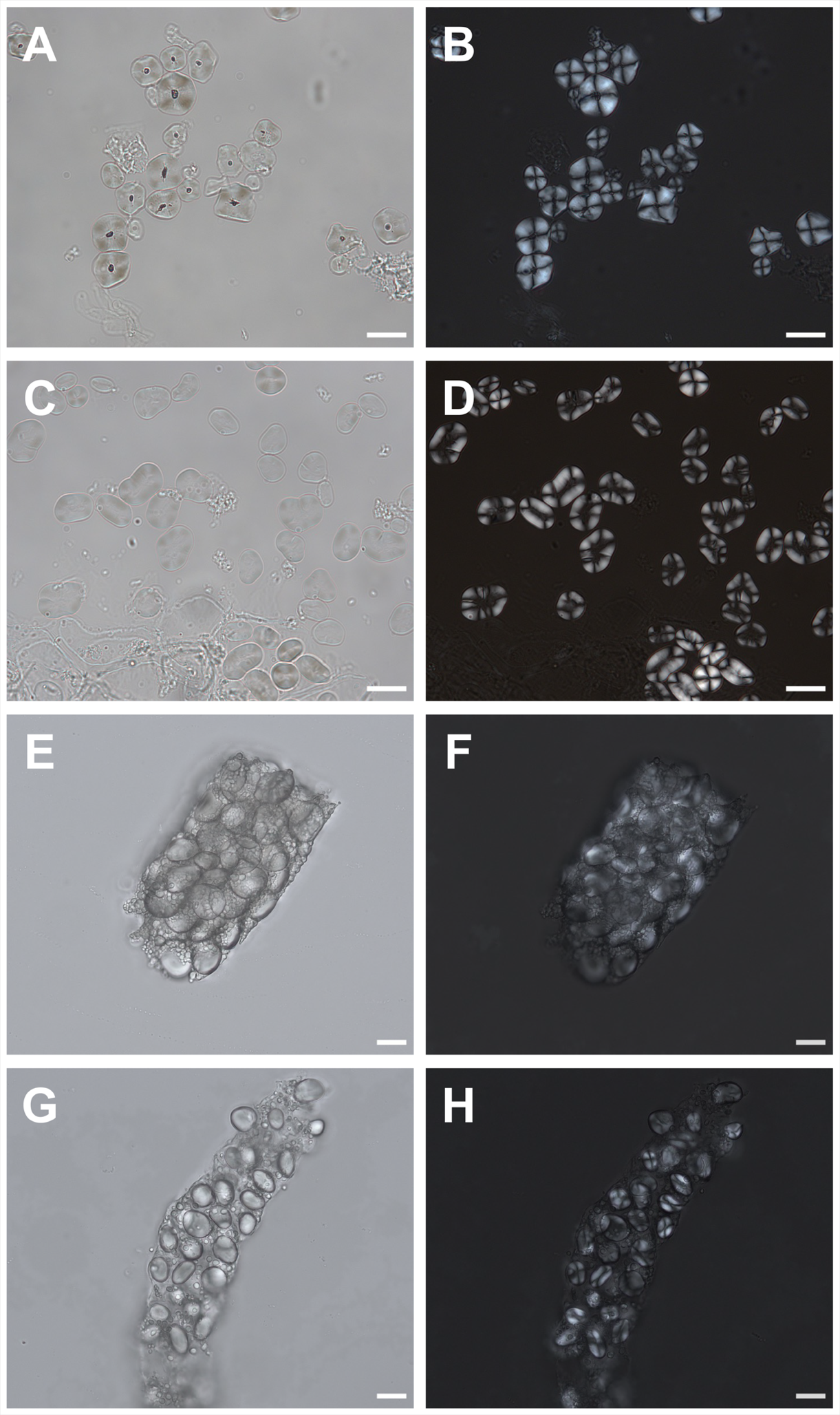


**S2 Fig. Reference starch granule morphotypes.**

(A­–B) Reference Panicoid spp. (*Sorghum bicolor* race *durra*), Type 1 and Type 2 native starch granules under plane (A) and cross-polarised light (B); (C–D) Reference Faboideae spp. (*Vigna unguiculata*), Type 3 and Type 4 native starch granules under plane (C) and cross-polarised light (D); (E–H) Reference Triticeae spp. (*Hordeum vulgare*), Type 5 native starch granule clusters with bimodal distribution under plane (E and G) and cross-polarised light (F and H). Scale bar (20 µm) applies to all panels.


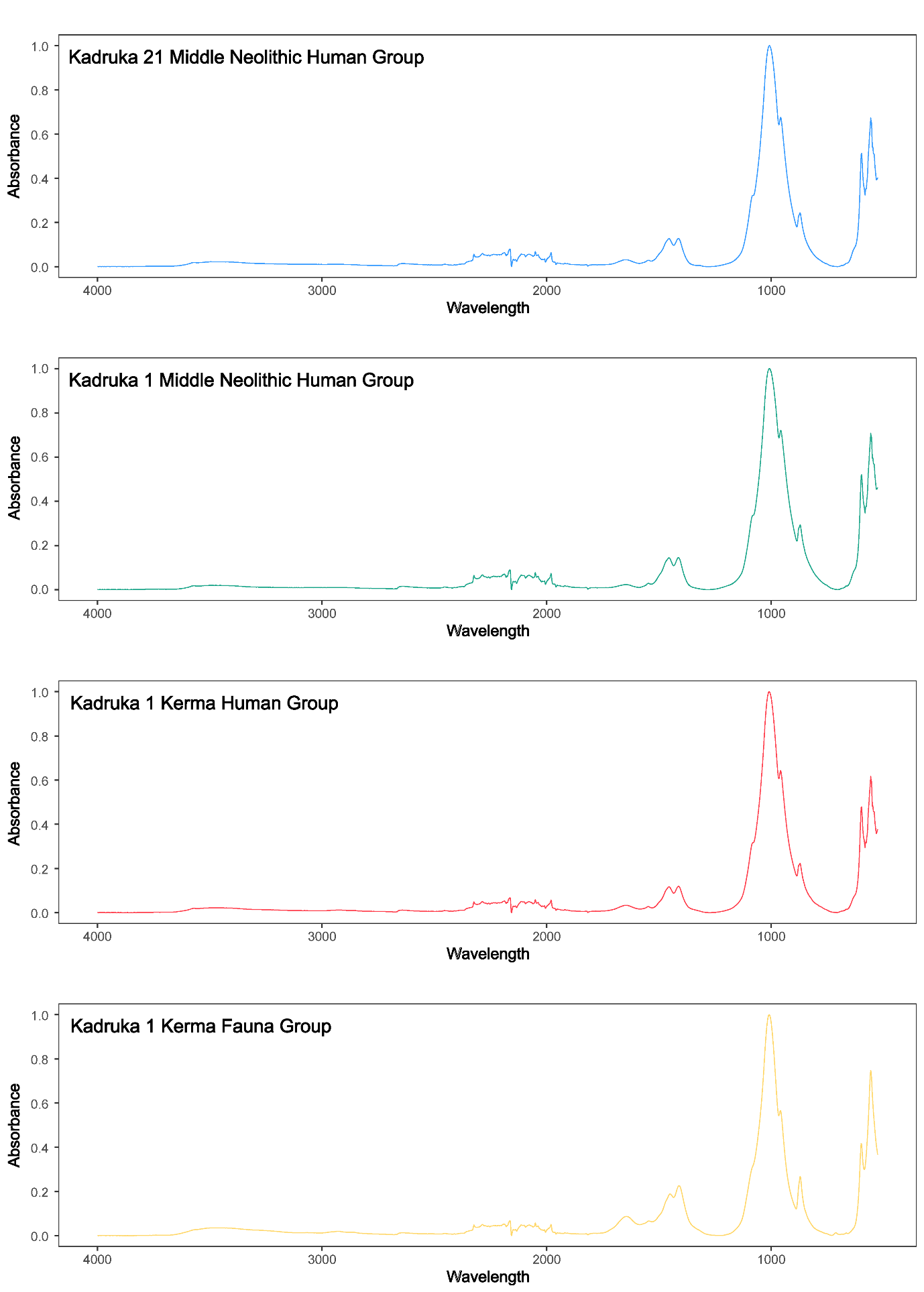


**S3 Fig. Averaged FTIR spectra for enamel samples from each group.**


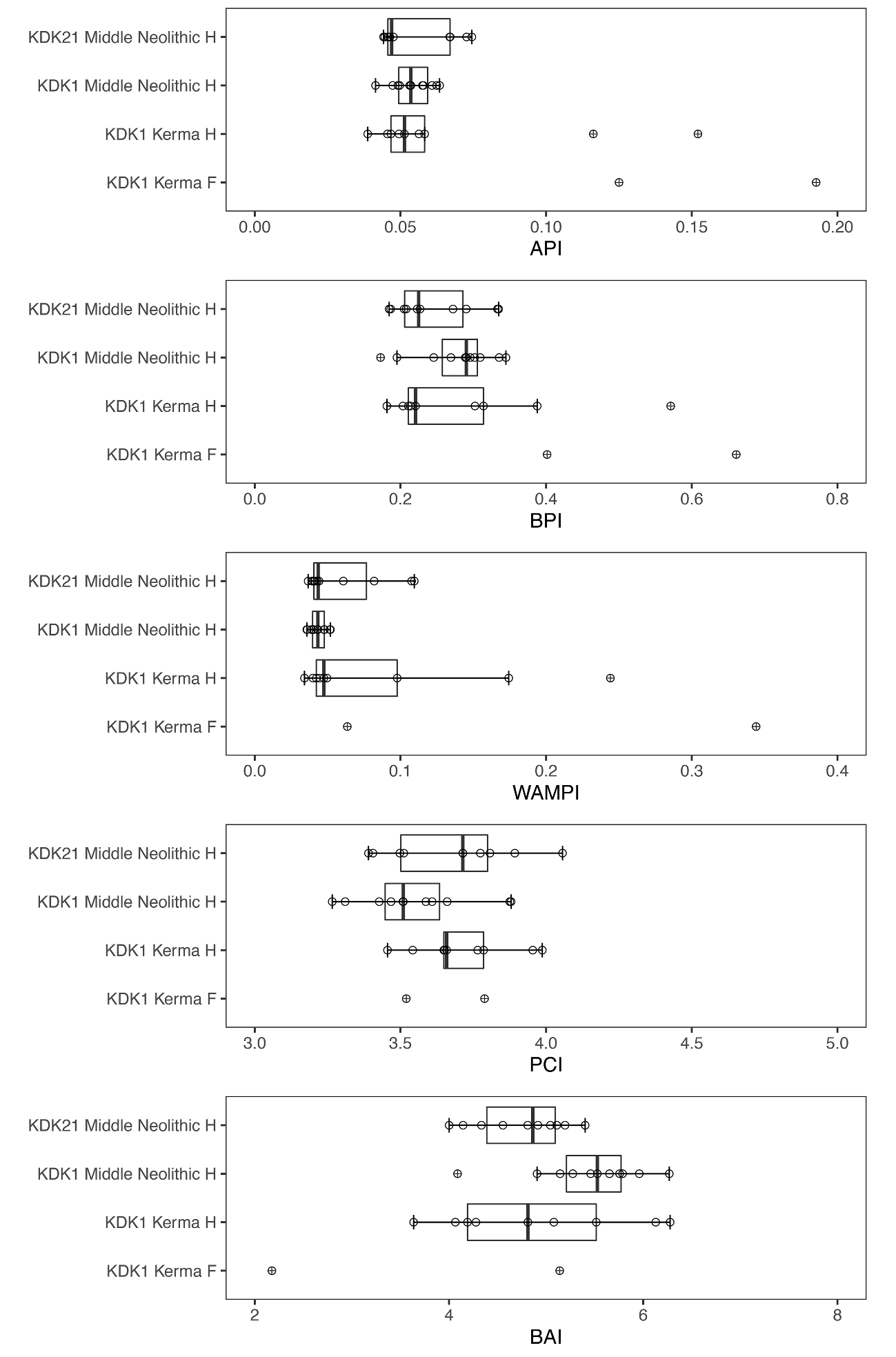


**S4 Fig. Box plots of the infrared indices (API, BPI, WAMPI, PCI, BAI).**

Determined using Fourier Transfer Infrared Spectroscopy for human and faunal sample groups as per Roche et al. [10] (see S7 Table).


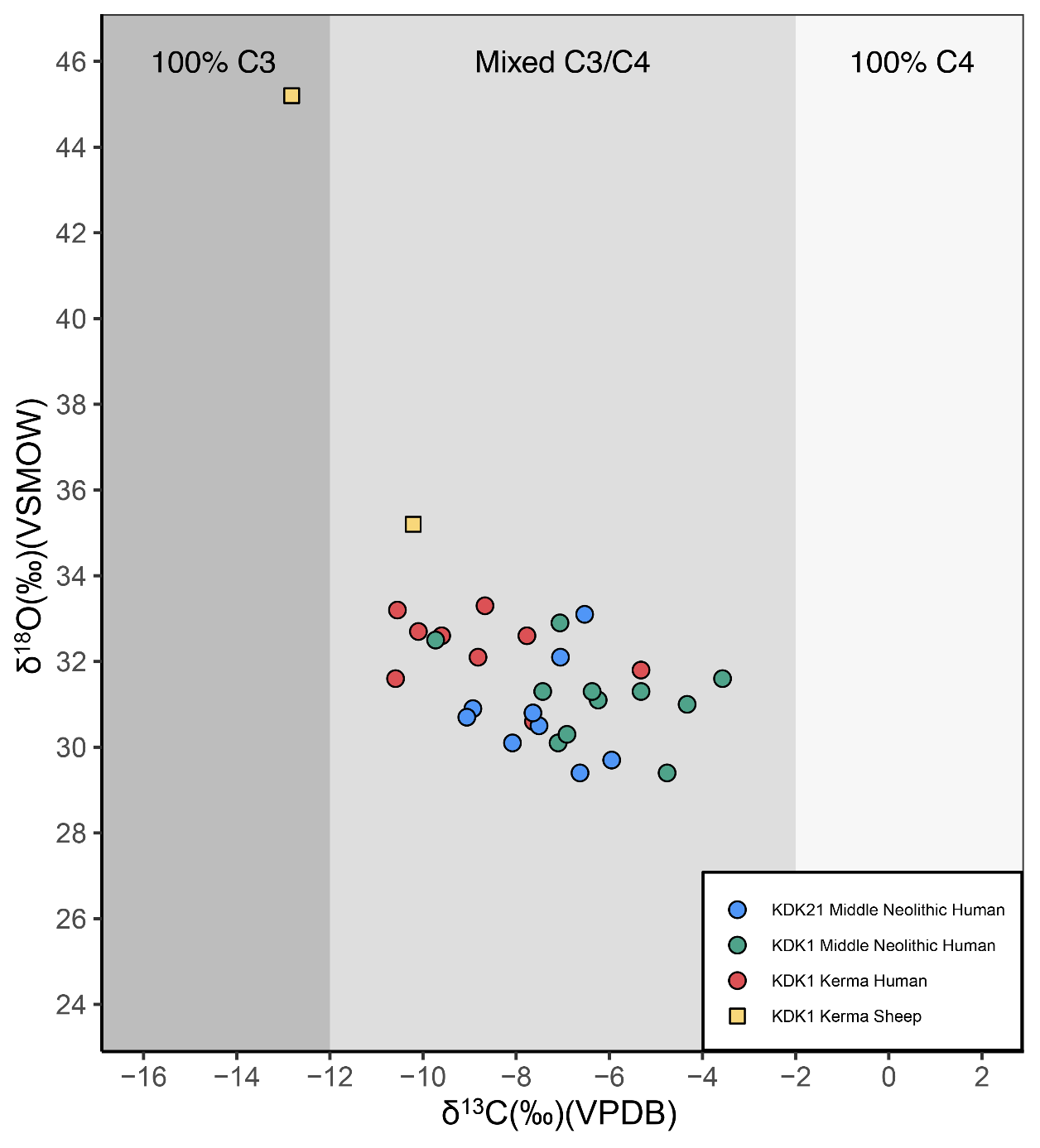


**S5 Fig. ẟ^13^C and ẟ^18^O measurements of human and faunal samples from KDK1 and KDK21.** Includes KDK1 Kerma faunal sample T22 with elevated ẟ^18^O value of 45.2‰ likely reflecting immature enamel. Shading indicates estimated carbonate ẟ^13^C for individuals consuming 100% C_3_, mixed C_3_/C_4_, and 100% C_4_ sources [11]. VPDB = Vienna Pee Dee Belemnite. VSMOW = Vienna Standard Mean Ocean Water.

**S1 Table. List of individuals with dental calculus selected for this study.**

Biological sex: M, Male; F, Female; PM, Probable Male: PF, Probable Female; INDT, Indeterminate.

* Individuals with associated sediment controls.

# Also sampled for δ^13^C, δ^15^N and ^14^C analysis of tooth dentine.

| Site | Phase | Individual ID * | Biological Sex | Age at Death | Sampled Tooth for Isotopic Analysis # |
| --- | --- | --- | --- | --- | --- |
| KDK1 | Kerma | SK 3 | M | 15 – 19 | Lower M3R # |
| KDK1 | Kerma | SK 9 | F | 30 – 40 | Lower M3L # |
| KDK1 | Kerma | SK 24 | F | 25 – 35 | Upper M3L # |
| KDK1 | Kerma | SK 26 | PF | 25 – 35 | Lower M3L # |
| KDK1 | Kerma | SK 38 | M | 25 – 35 | Lower M3R # |
| KDK1 | Kerma | SK 60 | PM | 5 – 9 | Upper M3R |
| KDK1 | Kerma | SK 63 | M | Adult | Lower M3R # |
| KDK1 | Kerma | SK 78 | PM | 5 – 9 | Upper M2R |
| KDK1 | Kerma | SK 87a | PM | Adult | Upper M3R |
| KDK1 | Middle Neolithic | SK 37 | INDT | 15 – 19 | – |
| KDK1 | Middle Neolithic | SK 39 | INDT | Adult | Lower M3L |
| KDK1 | Middle Neolithic | SK 42 * | INDT | Adult | – |
| KDK1 | Middle Neolithic | SK 55 * | F | >50 | Lower M3L |
| KDK1 | Middle Neolithic | SK 62 | M | 30 – 49 | Lower M3L # |
| KDK1 | Middle Neolithic | SK 69 | F | >50 | Lower M3R |
| KDK1 | Middle Neolithic | SK 74 | PM | 15 – 19 | Lower M3R |
| KDK1 | Middle Neolithic | SK 83 | PM | 15 – 19 | – |
| KDK1 | Middle Neolithic | SK 84 | INDT | 30 – 49 | – |
| KDK1 | Middle Neolithic | SK 88 | INDT | Adult | – |
| KDK1 | Middle Neolithic | SK 96 | PM | 15 – 19 | Upper M3L # |
| KDK1 | Middle Neolithic | SK 97 | INDT | Adult | Upper M3L # |
| KDK1 | Middle Neolithic | SK 99 * | INDT | Adult | – |
| KDK1 | Middle Neolithic | SK 101 | M | 30 – 49 | – |
| KDK1 | Middle Neolithic | SK 102 | INDT | Adult | – |
| KDK1 | Middle Neolithic | SK 106 | M | 30 – 49 | Upper M3L # |
| KDK1 | Middle Neolithic | SK 110 | F | 30 – 49 | – |
| KDK1 | Middle Neolithic | SK 125 | M | Adult | – |
| KDK1 | Middle Neolithic | SK 132 * | F | 30 – 49 | Upper M3L # |
| KDK1 | Middle Neolithic | SK 133 | M | Adult | Lower M3L |
| KDK1 | Middle Neolithic | SK 134 | M | >50 | Upper M3L # |
| KDK1 | Middle Neolithic | SK 139 | INDT | Adult | – |
| KDK21 | Middle Neolithic | SK 4 * | INDT | 30 – 49 | Lower M3L # |
| KDK21 | Middle Neolithic | SK 8 | INDT | Adult | – |
| KDK21 | Middle Neolithic | SK 22 | INDT | Adult | – |
| KDK21 | Middle Neolithic | SK 52 | INDT | Adult | – |
| KDK21 | Middle Neolithic | SK 76 | INDT | Adult | – |
| KDK21 | Middle Neolithic | SK 80 | INDT | 30 – 49 | Lower M3R # |
| KDK21 | Middle Neolithic | SK 87b * | PF | >50 | – |
| KDK21 | Middle Neolithic | SK 129 | INDT | Adult | – |
| KDK21 | Middle Neolithic | SK 131 | INDT | Adult | – |
| KDK21 | Middle Neolithic | SK 143 | PM | 10 – 14 | Lower M3R |
| KDK21 | Middle Neolithic | SK 172 | PM | 10 – 14 | Lower M2R |
| KDK21 | Middle Neolithic | SK 200 | PM | 5 – 9 | – |
| KDK21 | Middle Neolithic | SK 207 | M | 20 – 29 | – |
| KDK21 | Middle Neolithic | SK 223 | INDT | 20 – 29 | – |
| KDK21 | Middle Neolithic | SK 227 | INDT | Adult | – |
| KDK21 | Middle Neolithic | SK 234 | PM | 10 – 14 | – |
| KDK21 | Middle Neolithic | SK 236 * | INDT | Adult | Lower M3R # |
| KDK21 | Middle Neolithic | SK 237 | F | 20 – 39 | Upper M3L # |
| KDK21 | Middle Neolithic | SK 240 | INDT | Adult | Upper M3L # |
| KDK21 | Middle Neolithic | SK 247 | PM | Adult | – |
| KDK21 | Middle Neolithic | SK 254 | PM | Adult | – |
| KDK21 | Middle Neolithic | SK 264 | PM | Adult | Upper M3R # |
| KDK21 | Middle Neolithic | SK 268 | INDT | Adult | Lower M3L # |
| KDK21 | Middle Neolithic | SK 271 | INDT | Adult | Lower M3L # |

**S2 Table. List of fauna samples from KDK1 Kerma period graves selected for this study, classified by Chaix [12].**

Bone samples analysed for δ^13^C, δ^15^N and ^14^C. Enamel apatite analysed for δ^13^C and δ^18^O.

| Sample ID | Species | Age at Death | Sampled skeleton element |
| --- | --- | --- | --- |
| T1/2 | Capra hircus | Subadult | 2^nd^ phalange (bone collagen) |
| T5 | Ovis aries | Young | 1^st^ phalange (bone collagen) |
| T38 | Ovis aries | Subadult | 2^nd^ phalange (bone collagen) |
| T78 | Ovis aries | Young | 1^st^ phalange (bone collagen) |
| T95/2 | Ovis aries | Adult | 2^nd^ phalange (bone collagen) |
| T9 | Ovis aries | Young | Lower M1L (enamel apatite) |
| T22 | Ovis aries | Young | Lower M2R (enamel apatite) |

**Microparticle extraction protocols for sediments**

**S3 Table. Protocol for phytolith extraction from sediments through microwave digestion and heavy liquid flotation.**

Protocol adapted from Parr [13].

| 1. Samples placed into tared MARS Teflon tubes on scale and sample weights recorded |
| --- |
| 1. 3 ml of 70% Nitric Acid (HNO_3_) and 3 ml 32% Hydrochloric Acid (HCl) added to each sample |
| 1. Samples run through CEM MARSX microwave digestor, reaching maximum temperature of 170°C |
| 1. Samples decantated into pre-weighed 50 ml Falcon tubes and rinsed 3 times with di-ionised (dH_2_O) water at 3,500 rpm for 5 minutes |
| 1. 10 ml of 30% Hydrogen peroxide (H_2_O_2_) was then added to each sample and left overnight to remove organic component |
| 1. Rinsed 3 times with dH_2_O at 3,500 rpm for 5 minutes |
| 1. Samples dried at 37°C and weighed to record Acid Insoluble Fraction (AIF) |
| 1. 10 ml of specific gravity 2.3 sodium polytungstate (SPT) added to each sample and gently swirled to disperse extract |
| 1. Centrifuged at 3,000 rpm for 5 minutes, with supernatant decanted into new 50 ml tubes. |
| 1. Steps 8 and 9 repeated twice |
| 1. dH_2_O added to each sample to lower specific gravity and rinsed 3 times at 3,500 rpm for 5 minutes, supernatant poured into SPT recycling containers |
| 1. Phytolith extract transferred to pre-weighed 5 ml vials and placed in oven at 37°C to dry. Once dry, tubes reweighed to obtain phytolith extract weight |

**S4 Table. Protocol for starch granule extraction from sediments through deflocculation, oxidisation and heavy liquid flotation.**

Protocol adapted from Duncan [14]; Pearsall et al. [15]; Perry et al. [16].

| 1. Samples placed into tared 15 ml centrifuge tubes and sample weights recorded |
| --- |
| 1. Sample deflocculated with 5 ml of 0.1% EDTA for 2 hrs and rinsed 3 times with dH_2_O at 2,000 rpm for 10 minutes |
| 1. 2 ml of 5% H_2_O_2_ added to each sample and left to sit at room temperature for 30 minutes to oxidise |
| 1. Rinsed 3 times with dH_2_O at 2,000 rpm for 10 minutes |
| 1. 0.3 µm glass fibre filters placed over the top of each sample tube with samples dried at 37°C |
| 1. 2 ml of specific gravity 1.8 SPT added to each sample and gently swirled to disperse extract |
| 1. Centrifuged at 2,000 rpm for 10 minutes, with supernatant decanted into new autoclaved 15 ml tubes |
| 1. Steps 6 and 7 repeated 4 times to maximise recovery of starch granules |
| 1. dH_2_O added to each sample to lower specific gravity and rinsed 3 times at 2,000 rpm for 10 minutes, supernatant poured into SPT recycling containers |
| 1. Starch extract transferred to pre-weighed autoclaved 5 ml tubes |
| 1. Sampled dried at 37°C with 0.3 µm glass fibre filters over the top of each ample. Once dry, tubes reweighed to obtain starch extract weight |

**S5 Table. Isotopic and AMS radiocarbon results on collagen extracted from faunal bone and human tooth dentine, published radiocarbon dates for KDK1 and KDK21, and modelled ages.**

| Sample | Site / Period | Material, species | Laboratory code | % Collagen | C:N | ẟ^13^C | ẟ^15^N | ^14^C Age (BP) | 1σ | cal BCE* | Median cal BCE | Modelled Age BCE (95% CI) | Median Modelled Age BCE |
| --- | --- | --- | --- | --- | --- | --- | --- | --- | --- | --- | --- | --- | --- |
| SK 3 | KDK1 Kerma | Dentine, Homo sapiens | OZAC35 | 0.39 | 3.58 | -15.9 | 15.2 | 3650 | 25 | 2135–1941 | 2018 | 2135–1941 | 2018 |
| SK 26** | KDK1 Kerma | Dentine, Homo sapiens | OZAC37 | 0.49 | 3.60 | -13.3 | 14.5 | 3685 | 25 | 2282–2053 | 2167 | 2282–2053 | 2167 |
| SK 38 | KDK1 Kerma | Dentine, Homo sapiens | OZAC38 | 0.94 | 3.56 | -13.9 | 15.3 | 3720 | 25 | 2205–2036 | 2139 | 2205–2036 | 2139 |
| SK 63 | KDK1 Kerma | Dentine, Homo sapiens | OZAC39 | 0.20 | 3.47 | -14.0 | 16.0 | 3780 | 25 | 2191–1976 | 2082 | 2191–1976 | 2082 |
| T95/2 | KDK1 Kerma | Bone, Ovis aries | OZAC64 | 4.15 | 3.36 | -12.6 | 10.5 | 3740 | 25 | 2192–1976 | 2082 | 2191–1976 | 2082 |
| T1/2 | KDK1 Kerma | Bone, Capra hircus | OZAC95 | 3.94 | 3.51 | -17.9 | 8.4 | 3550 | 25 | 2016–1784 | 1915 | 2016–1784 | 1915 |
| T5 | KDK1 Kerma | Bone, Ovis aries | OZAC96 | 0.54 | 3.46 | -15.1 | 12.1 | 3550 | 25 | 2016–1915 | 1915 | 2016–1785 | 1915 |
| SK 68 | KDK1 Kerma | Hair, Homo sapiens | GrM17738 | – | 3.15 | -17.2 | 12.03 | 3685 | 25 | 2199–2032 | 2092 | 2199–2032 | 2092 |
| –  s1 | KDK1 Middle Neolithic | Shell, *Aspatharia* spp. | – | – | – | – | – | 5590 | 60 | 4544–4337 | 4422 | 4544–4337 | 4422 |
| SK 131  s2 | KDK1 Middle Neolithic | Shell, *Aspatharia* spp. | – | – | – | – | – | 5360 | 70 | 4342–3999 | 4189 | 4342–3999 | 4189 |
| –  s3 | KDK1 Middle Neolithic | Shell, *Aspatharia* spp. | – | – | – | – | – | 5290 | 80 | 4327–3967 | 4132 | 4327–3967 | 4132 |
| –  s1 | KDK21 Middle Neolithic | Shell, *Aspatharia* spp. | – | – | – | – | – | 5910 | 60 | 4935–4615 | 4774 | 4935–4615 | 4774 |
| –  s2 | KDK21 Middle Neolithic | Shell, *Aspatharia* spp. | – | – | – | – | – | 5890 | 60 | 4907–4557 | 4756 | 4907–4557 | 4756 |
| –  s3 | KDK21 Middle Neolithic | Shell, *Aspatharia* spp. | – | – | – | – | – | 5875 | 110 | 4982–4459 | 4726 | 4982–4459 | 4726 |
| –  s4 | KDK21 Middle Neolithic | Shell, *Aspatharia* spp. | – | – | – | – | – | 5850 | 70 | 4895–4537 | 4709 | 4895–4537 | 4709 |

*Calendar ages at 95.4% confidence level were obtained from age calibration using the IntCal20 calibration curve [17] and OxCal program v4.4.2 [18]. **Collagen extraction without the ultrafiltration step. Kerma ^14^C date for KDK1 individual SK 68 reported in Bleasdale et al. [19], C:N ratio for hair within accepted keratin values of 3.0–3.8 [20]. Middle Neolithic ^14^C dates for KDK21 and KDK1 reported in Reinold [21, 22], no further information provided. GrM, University of Groningen, Netherlands; OZ, ANSTO, Australia.

**S6 Table. Difference in phytolith assemblage composition between dental calculus fractions.**

Comparison of phytolith morphotype diversity and results of Jaccard similarity index for nine samples with associated powder and solid fractions. Jaccard values; 1 = identical morphotype distribution, 0 = completely dissimilar morphotype distribution [23].

| Skeleton ID | Calculus Fraction | Extract Weight (mg) | # Distinct Diagnostic Morphotypes | Jaccard Similarity Index |
| --- | --- | --- | --- | --- |
| SK 9 | Solid | 0.72 | 4 | 0.25 |
|  | Powder | 2.47 | 6 |  |
| SK 24 | Solid | 0.89 | 2 | 0.33 |
|  | Powder | 1.75 | 2 |  |
| SK 55 | Solid | 0.83 | 8 | 0.66 |
|  | Powder | 1.63 | 10 |  |
| SK 62 | Solid | 0.46 | 11 | 0.60 |
|  | Powder | 1.48 | 14 |  |
| SK 88 | Solid | 0.69 | 12 | 0.25 |
|  | Powder | 0.14 | 3 |  |
| SK 96 | Solid | 0.39 | 2 | 0.12 |
|  | Powder | 0.62 | 7 |  |
| SK 132 | Solid | 0.32 | 5 | 0.25 |
|  | Powder | 0.44 | 5 |  |
| SK 134 | Solid | 0.22 | 7 | 0.33 |
|  | Powder | 1.37 | 12 |  |
| SK 240 | Solid | 1.40 | 2 | 0.33 |
|  | Powder | 1.75 | 2 |  |

**S7 Table. Results of Analysis of Similarity by sample group, based on the presence or absence of diagnostic phytolith morphotypes in dental calculus.**

Conducted to measure the significance of observed group separation by comparing the mean of ranked between group to within group dissimilarities [24]. R value 0 = greater within group dissimilarity than between groups, 1 = complete dissimilarity between groups.

| ANOSIM statistic R: **0.2862** | | | | |  | |
| --- | --- | --- | --- | --- | --- | --- |
| Significance: **0.0001** | | | | |  |  |
| Permutation: free | | | | |  |  |
| Number of permutations: 9999 | | | | |  |  |
| Upper quantiles of permutations (null model): | | | | |  |  |
|  | 90% | 95% | 97.5% | 99% |  |  |
|  | 0.0730 | 0.0972 | 0.1188 | 0.1423 |  |  |
| Dissimilarity ranks between and within classes: | | | | |  |  |
|  | 0% | 25% | 50% | 75% | 100% | N |
| Between | 2.0 | 180.75 | 329.5 | 445.50 | 519.0 | 364 |
| Kadruka 1 Kerma | 3.0 | 128.50 | 274.04 | 71.25 | 519 | 15 |
| Kadruka 1 Middle Neolithic | 3.0 | 57.25 | 128.5 | 213.25 | 436.5 | 91 |
| Kadruka 21 Middle Neolithic | 5.5 | 152.00 | 299.00 | 413.50 | 519.0 | 91 |

**S8 Table. Description of the FTIR spectrum of enamel bioapatite.**

Based on LeGeros [25]; Michel et al. [26]; Sponheimer and Lee-Thorp [27]; Trueman et al. [28]; and Roche et al. [10].

| Approximate positions  of apparent bands (cm^-1^) | Approximate positions of decomposed bands (cm^-1^) | Chemical groups | Location of chemical groups | Chemical bonds and Vibrational modes |
| --- | --- | --- | --- | --- |
| Dome between 2,400 and 3,800 | 3,570 | Hydroxyl (OH) | A-site of apatite | O-H Stretching |
|  | between 3,300 and 3,430 (two bands at least) | Water (H_2_O) | Adsorbed on apatite surface and A-site of apatite | O-H Stretching ${}_{1}$ and ${}_{3}$ |
| 1,650 | Between 1,660 and 1,670 | Amide (CO-NH) | Organic matrix | C=O Stretching ${}_{1}$ |
|  | Between 1,620 and 1,640 | Water (H_2_O) | Adsorbed on apatite surface and A-site of apatite? | O-H Bending ${}_{2}$ |
| 1,540 | 1,545 | Carbonate (CO_3_) | A-site of apatite | C-O Stretching ${}_{3}$ |
| 1,450 | 1,500 | Carbonate (CO_3_) | Not determined | C-O Stretching ${}_{3}$ |
|  | 1,470 | Carbonate (CO_3_) | B-site of apatite and adsorbed on apatite surface? | C-O Stretching ${}_{3}$ |
|  | 1, 450 | Carbonate (CO_3_) | A-site of apatite and adsorbed on apatite surface? | C-O Stretching ${}_{3}$ |
| 1,415 | 1,415 | Carbonate (CO_3_) | B-site of apatite | C-O Stretching ${}_{3}$ |
| 1,090 | 1,090 | Phosphate (PO_4_) | B-site of apatite | P-O Stretching ${}_{3}$ |
| 1,040 | 1,040 | Phosphate (PO_4_) | B-site of apatite | P-O Stretching ${}_{3}$ |
| 960 | 960 | Phosphate (PO_4_) | B-site of apatite | P-O Stretching ${}_{3}$ |
| 880 | 880 | Carbonate (CO_3_) | A-site of apatite | C-O Bending ${}_{2}$ |
| 875 | 875 | Carbonate (CO_3_)  Hydrogenoph-osphate? | B-site of apatite  B-site of apatite | C-O Bending ${}_{2}$  P-O Stretching ${}_{3}$ |
|  | 865 | Carbonate (CO_3_) | Not determined | C-O Bending ${}_{2}$ |
| 605 | 630 | Hydroxyl (OH) | A-site of apatite | O-H bending |
|  | 605 | Phosphate (PO_4_) | B-site of apatite | P-O Bending ${}_{4}$ |
| 565 | 575 | Phosphate (PO_4_) | B-site of apatite | P-O Bending ${}_{4}$ |
|  | 565 | Phosphate (PO_4_) | B-site of apatite | P-O Bending ${}_{4}$ |
| 470 | 470 | Phosphate (PO_4_) | B-site of apatite | P-O Bending ${}_{4}$ |

**S9 Table. Description of the FTIR indices of enamel bioapatite.**

B and V denote the height of the bands and the valleys, respectively. Numbers in parentheses represent the approximate positions of the apparent bands (adapted from Roche et al. [10]).

| Indices | Formulas | References |
| --- | --- | --- |
| **PCI** (Phosphate Crystallinity Index) other names: **CI_IR_** (Crystallinity Index _InfraRed_)  **IRSF** (InfraRed Splitting Factor) | $\frac{\text{B}\left( \text{605} \right)\text{+B(565)}}{\text{V(590)}}$ | Sponheimer and Lee-Thorp [27]  Shemesh [29]  Weiner and Bar-Yosef [30] |
| **BPI** (B-carbonate on Phosphate Index) | $\frac{\text{B(1415)}}{\text{B(605)}}$ | LeGeros [25] |
| **API** (A-carbonate on Phosphate Index) | $\frac{\text{B(1545)}}{\text{B(605)}}$ | Sponheimer and Lee-Thorp [27] |
| **BAI** (relative amount of B- to A-site carbonate) | $\frac{\text{B}\left( \text{1415} \right)}{\text{B}\text{(}\text{1540}\text{)}}$ | Sponheimer [31]; Sponheimer and Lee-Thorp [27] |
| **WAMPI** (Water-Amide on Phosphate Index) | $\frac{\text{B(1650)}}{\text{B(605)}}$ | Roche et al. [10] |

**S10 Table. Table of the infrared indexes (WAMPI, API, BPI, PCI, BAI) for sample groups subjected to Fourier-Transform Infrared Spectroscopy diagenesis study (as per Roche et al. [10]).**

* denotes adolescent/young individuals.

| Individual ID. | Group | API | BPI | WAMPI | PCI | BAI |
| --- | --- | --- | --- | --- | --- | --- |
| SK 3 | KDK1 K Human | 0.05 | 0.20 | 0.05 | 3.65 | 4.19 |
| SK 9 | KDK1 K Human | 0.05 | 0.21 | 0.03 | 3.66 | 5.08 |
| SK 24 | KDK1 K Human | 0.06 | 0.30 | 0.04 | 3.46 | 5.52 |
| SK 26 | KDK1 K Human | 0.15 | 0.57 | 0.17 | 3.54 | 4.07 |
| SK 38 | KDK1 K Human | 0.05 | 0.22 | 0.04 | 3.77 | 4.81 |
| SK 60 * | KDK1 K Human | 0.12 | 0.39 | 0.24 | 3.65 | 3.64 |
| SK 63 | KDK1 K Human | 0.05 | 0.18 | 0.04 | 3.79 | 4.28 |
| SK 78 * | KDK1 K Human | 0.06 | 0.31 | 0.10 | 3.95 | 6.28 |
| SK 87a | KDK1 K Human | 0.04 | 0.21 | 0.05 | 3.99 | 6.13 |
| T9 * | KDK1 K Fauna | 0.13 | 0.66 | 0.06 | 3.79 | 5.14 |
| T22 * | KDK1 K Fauna | 0.19 | 0.40 | 0.34 | 3.52 | 2.18 |
| SK 39 | KDK1 MN Human | 0.05 | 0.25 | 0.04 | 3.59 | 5.46 |
| SK 55 | KDK1 MN Human | 0.06 | 0.34 | 0.05 | 3.31 | 6.27 |
| SK 62 | KDK1 MN Human | 0.06 | 0.31 | 0.04 | 3.47 | 5.65 |
| SK 69 | KDK1 MN Human | 0.05 | 0.27 | 0.04 | 3.51 | 5.76 |
| SK 74 | KDK1 MN Human | 0.04 | 0.20 | 0.04 | 3.88 | 5.14 |
| SK 96 | KDK1 MN Human | 0.05 | 0.17 | 0.05 | 3.88 | 4.09 |
| SK 97 | KDK1 MN Human | 0.06 | 0.34 | 0.05 | 3.27 | 5.53 |
| SK 106 | KDK1 MN Human | 0.05 | 0.29 | 0.04 | 3.51 | 5.79 |
| SK 132 | KDK1 MN Human | 0.06 | 0.29 | 0.05 | 3.43 | 4.91 |
| SK 133 | KDK1 MN Human | 0.05 | 0.30 | 0.04 | 3.61 | 5.96 |
| SK 134 | KDK1 MN Human | 0.06 | 0.30 | 0.05 | 3.66 | 5.27 |
| SK 4 | KDK21 MN Human | 0.07 | 0.33 | 0.08 | 3.41 | 5.04 |
| SK 80 | KDK21 MN Human | 0.04 | 0.22 | 0.04 | 3.89 | 5.40 |
| SK 143 | KDK21 MN Human | 0.05 | 0.23 | 0.04 | 3.81 | 5.11 |
| SK 172 * | KDK21 MN Human | 0.05 | 0.19 | 0.04 | 3.71 | 4.33 |
| SK 236 | KDK21 MN Human | 0.05 | 0.21 | 0.04 | 3.71 | 4.81 |
| SK 237 | KDK21 MN Human | 0.07 | 0.27 | 0.11 | 3.77 | 4.00 |
| SK 240 | KDK21 MN Human | 0.07 | 0.29 | 0.11 | 3.50 | 4.15 |
| SK 264 | KDK21 MN Human | 0.07 | 0.33 | 0.06 | 3.39 | 5.20 |
| SK 268 | KDK21 MN Human | 0.05 | 0.21 | 0.04 | 3.51 | 4.92 |
| SK 271 | KDK21 MN Human | 0.04 | 0.18 | 0.04 | 4.06 | 4.56 |

**S11 Table. Results of ANOVA for API of diagenesis study groups.**

|  | Degrees of freedom | Sum of squares | Mean Square | F value | Pr (>F)* |
| --- | --- | --- | --- | --- | --- |
| Group | 2 | 0.001 | 0.00 | 1.493 | 0.243 |
| Residuals | 27 | 0.013 | 0.00 |  |  |

*p=<0.05

**S12 Table. Results of post-hoc Tukey pair-wise comparison for API of diagenesis study groups.**

95% confidence interval of difference is indicated alongside 95% probability of lower and upper bounds of this difference.

| Group | Difference | Lower | Upper | *p*-value* |
| --- | --- | --- | --- | --- |
| KDK1 MN Human – KDK1 K Human | -0.016 | -0.041 | 0.008 | 0.251 |
| KDK21 MN Human – KDK1 K Human | -0.014 | -0.039 | 0.011 | 0.373 |
| KDK21 MN Human – KDK1 MN Human | 0.002 | -0.021 | 0.026 | 0.968 |

*p=<0.05

**S13 Table. Results of ANOVA for BPI of diagenesis study groups.**

|  | Degrees of freedom | Sum of squares | Mean Square | F value | Pr (>F) |
| --- | --- | --- | --- | --- | --- |
| Group | 2 | 0.009 | 0.004 | 0.689 | 0.511 |
| Residuals | 27 | 0.182 | 0.006 |  |  |

*p=<0.05

**S14 Table. Results of post-hoc Tukey pair-wise comparison for BPI of diagenesis study groups.**

95% confidence interval of difference is indicated alongside 95% probability of lower and upper bounds of this difference.

| Group | Difference | Lower | Upper | *p*-value* |
| --- | --- | --- | --- | --- |
| KDK1 MN Human-KDK1 K Human | -0.009 | -0.101 | 0.082 | 0.963 |
| KDK21 MN Human-KDK1 K Human | -0.041 | -0.135 | 0.052 | 0.519 |
| KDK21 MN Human-KDK1 MN Human | -0.032 | -0.121 | 0.056 | 0.648 |

*p=<0.05

**S15 Table. Results of ANOVA for WAMPI of diagenesis study groups.**

|  | Degrees of freedom | Sum of squares | Mean Square | F value | Pr (>F) |
| --- | --- | --- | --- | --- | --- |
| Group | 2 | 0.007 | 0.004 | 2.093 | 0.143 |
| Residuals | 27 | 0.051 | 0.001 |  |  |

*p=<0.05

**S16 Table. Results of post-hoc Tukey pair-wise comparison for WAMPI of diagenesis study groups.**

95% confidence interval of difference is indicated alongside 95% probability of lower and upper bounds of this difference.

| Group | Difference | Lower | Upper | *p*-value* |
| --- | --- | --- | --- | --- |
| KDK1 MN Human-KDK1 K Human | -0.039 | -0.088 | 0.008 | 0.121 |
| KDK21 MN Human-KDK1 K Human | -0.024 | -0.074 | 0.025 | 0.450 |
| KDK21 MN Human-KDK1 MN Human | 0.015 | -0.031 | 0.062 | 0.698 |

*p=<0.05

**S17 Table. Results of ANOVA for PCI of diagenesis study groups.**

|  | Degrees of freedom | Sum of squares | Mean Square | F value | Pr (>F) |
| --- | --- | --- | --- | --- | --- |
| Group | 2 | 0.143 | 0.071 | 1.805 | 0.184 |
| Residuals | 27 | 0.074 | 0.039 |  |  |

*p=<0.05

**S18 Table. Results of post-hoc Tukey pair-wise comparison for PCI of diagenesis study groups.**

95% confidence interval of difference is indicated alongside 95% probability of lower and upper bounds of this difference.

| Group | Difference | Lower | Upper | *p*-value* |
| --- | --- | --- | --- | --- |
| KDK1 MN Human-KDK1 K Human | -0.161 | -0.383 | 0.060 | 0.188 |
| KDK21 MN Human-KDK1 K Human | -0.041 | -0.269 | 0.185 | 0.892 |
| KDK21 MN Human-KDK1 MN Human | 0.119 | -0.096 | 0.335 | 0.369 |

*p=<0.05

**S19 Table. Results of ANOVA for BAI of diagenesis study groups.**

|  | Degrees of freedom | Sum of squares | Mean Square | F value | Pr (>F) |
| --- | --- | --- | --- | --- | --- |
| Group | 2 | 2.786 | 1.393 | 3.016 | 0.065 |
| Residuals | 27 | 12.471 | 0.461 |  |  |

*p=<0.05

**S20 Table. Results of post-hoc Tukey pair-wise comparison for BAI of diagenesis study groups.**

95% confidence interval of difference is indicated alongside 95% probability of lower and upper bounds of this difference.

| Group | Difference | Lower | Upper | *p*-value* |
| --- | --- | --- | --- | --- |
| KDK1 MN Human-KDK1 K Human | 0.550 | -0.207 | 1.307 | 0.188 |
| KDK21 MN Human-KDK1 K Human | -0.136 | -0.911 | 0.637 | 0.899 |
| KDK21 MN Human-KDK1 MN Human | -0.687 | -1.423 | 0.049 | 0.070 |

*p=<0.05

**S21 Table. Bulk δ^13^C and δ^18^O measurements of enamel human and faunal specimens from KDK1 and KDK21.**

* denotes adolescent/young individuals.

| Site | Phase | Individual ID | Species | Tooth | δ^13^C (‰) (VPDB^a^) | S.D. | δ^18^O (‰) (VSMOW^b^) | S.D.^c^ |
| --- | --- | --- | --- | --- | --- | --- | --- | --- |
| Kadruka 1 | Kerma | T 9 * | Ovis aries | Lower M1L | -10.21 | 0.03 | 35.2 | 0.09 |
| Kadruka 1 | Kerma | T 22 * | Ovis aries | Lower M2R | -12.82 | 0.03 | 45.2 | 0.09 |
| Kadruka 1 | Kerma | SK 3 | Homo sapiens | Lower M3R | -10.59 | 0.03 | 31.6 | 0.09 |
| Kadruka 1 | Kerma | SK9 | Homo sapiens | Lower M3L | -8.67 | 0.03 | 33.3 | 0.09 |
| Kadruka 1 | Kerma | SK 24 | Homo sapiens | Upper M3L | -10.10 | 0.03 | 32.7 | 0.09 |
| Kadruka 1 | Kerma | SK 26 | Homo sapiens | Lower M3L | -7.77 | 0.03 | 32.6 | 0.09 |
| Kadruka 1 | Kerma | SK 38 | Homo sapiens | Lower M3R | -8.82 | 0.03 | 32.1 | 0.09 |
| Kadruka 1 | Kerma | SK 60 * | Homo sapiens | Upper M3R | -7.63 | 0.03 | 30.6 | 0.09 |
| Kadruka 1 | Kerma | SK 63 | Homo sapiens | Lower M3R | -9.60 | 0.03 | 32.6 | 0.09 |
| Kadruka 1 | Kerma | SK 78 * | Homo sapiens | Upper M2R | -10.55 | 0.03 | 33.2 | 0.09 |
| Kadruka 1 | Kerma | SK 87a | Homo sapiens | Upper M3R | -5.32 | 0.03 | 31.8 | 0.09 |
| Kadruka 1 | Middle Neolithic | SK 39 | Homo sapiens | Lower M3L | -4.33 | 0.03 | 31.0 | 0.09 |
| Kadruka 1 | Middle Neolithic | SK 55 | Homo sapiens | Lower M3L | -7.10 | 0.03 | 30.1 | 0.09 |
| Kadruka 1 | Middle Neolithic | SK 62 | Homo sapiens | Lower M3L | -6.24 | 0.03 | 31.1 | 0.09 |
| Kadruka 1 | Middle Neolithic | SK 69 | Homo sapiens | Lower M3R | -7.43 | 0.03 | 31.3 | 0.09 |
| Kadruka 1 | Middle Neolithic | SK 74 | Homo sapiens | Lower M3R | -6.91 | 0.03 | 30.3 | 0.09 |
| Kadruka 1 | Middle Neolithic | SK 96 | Homo sapiens | Upper M3L | -5.32 | 0.03 | 31.3 | 0.09 |
| Kadruka 1 | Middle Neolithic | SK 97 | Homo sapiens | Upper M3L | -4.76 | 0.03 | 29.4 | 0.09 |
| Kadruka 1 | Middle Neolithic | SK 106 | Homo sapiens | Upper M3L | -9.73 | 0.03 | 32.5 | 0.09 |
| Kadruka 1 | Middle Neolithic | SK 132 | Homo sapiens | Upper M3L | -7.06 | 0.03 | 32.9 | 0.09 |
| Kadruka 1 | Middle Neolithic | SK 133 | Homo sapiens | Lower M3R | -3.57 | 0.03 | 31.6 | 0.09 |
| Kadruka 1 | Middle Neolithic | SK 134 | Homo sapiens | Upper M3L | -6.37 | 0.03 | 31.3 | 0.09 |
| Kadruka 21 | Middle Neolithic | SK 4 | Homo sapiens | Lower M3L | -5.95 | 0.03 | 29.7 | 0.09 |
| Kadruka 21 | Middle Neolithic | SK 80 | Homo sapiens | Lower M3R | -8.93 | 0.03 | 30.9 | 0.09 |
| Kadruka 21 | Middle Neolithic | SK 143 | Homo sapiens | Lower M3R | -7.51 | 0.03 | 30.5 | 0.09 |
| Kadruka 21 | Middle Neolithic | SK 172 * | Homo sapiens | Lower M2R | -7.64 | 0.03 | 30.8 | 0.09 |
| Kadruka 21 | Middle Neolithic | SK 236 | Homo sapiens | Lower M3R | -8.08 | 0.03 | 30.1 | 0.09 |
| Kadruka 21 | Middle Neolithic | SK 237 | Homo sapiens | Upper M3L | -7.55 | 0.03 | 25.9 | 0.09 |
| Kadruka 21 | Middle Neolithic | SK 240 | Homo sapiens | Upper M3L | -9.06 | 0.03 | 30.7 | 0.09 |
| Kadruka 21 | Middle Neolithic | SK 264 | Homo sapiens | Upper M3R | -7.05 | 0.03 | 31.1 | 0.09 |
| Kadruka 21 | Middle Neolithic | SK 268 | Homo sapiens | Lower M3L | -6.63 | 0.03 | 29.4 | 0.09 |
| Kadruka 21 | Middle Neolithic | SK 271 | Homo sapiens | Lower M3L | -6.53 | 0.03 | 33.1 | 0.09 |

^a^ VPDB = Vienna Pee-Dee Belemnite

^b^ VSMOW = Vienna Standard Mean Ocean Water

^c^ S.D. = standard deviation

**S22 Table. Results of ANOVA for δ^18^O (VSMOW) by group.**

|  | Degrees of freedom | Sum of squares | Mean Square | F value | Pr (>F) |
| --- | --- | --- | --- | --- | --- |
| Group | 2 | 18.2 | 9.102 | 5.084 | 0.013 |
| Residuals | 27 | 48.34 | 1.79 |  |  |

*p=<0.05

**S23 Table. Results of post-hoc Tukey pair-wise comparison for δ^18^O (VSMOW) by group.**

95% confidence interval of difference is indicated alongside 95% probability of lower and upper bounds of this difference.

| Group | Difference | Lower | Upper | *p*-value* |
| --- | --- | --- | --- | --- |
| KDK1 MN H – KDK1 K H | -1.114 | -2.605 | 0.377 | 0.172 |
| KDK21 MN H – KDK1 K H | -1.958 | -3.482 | -0.434 | 0.010 |
| KDK21 MN H – KDK1 MN H | -0.844 | -2.293 | 0.606 | 0.334 |

*p=<0.05

**S24 Table. Results of ANOVA for δ^18^O (VSMOW) by group with outlier (SK 237) removed.**

|  | Degrees of freedom | Sum of squares | Mean Square | F value | Pr (>F) |
| --- | --- | --- | --- | --- | --- |
| Group | 2 | 10.67 | 5.335 | 5.209 | 0.012 |
| Residuals | 26 | 26.63 | 1.024 |  |  |

*p=<0.05

**S25 Table. Results of post-hoc Tukey pair-wise comparison for δ^18^O (VSMOW) by group with outlier (SK 237) removed.**

95% confidence interval of difference is indicated alongside 95% probability of lower and upper bounds of this difference.

| Group | Difference | Lower | Upper | *p*-value* |
| --- | --- | --- | --- | --- |
| KDK1 MN H – KDK1 K H | -1.114 | -2.244 | 0.016 | 0.053 |
| KDK21 MN H – KDK1 K H | -1.466 | -2.652 | -0.281 | 0.013 |
| KDK21 MN H – KDK1 MN H | -0.352 | -1.482 | 0.777 | 0.721 |

*p=<0.05

**S26 Table. Results of ANOVA for δ^13^C (VPDB) by group.**

|  | Degrees of freedom | Sum of squares | Mean Square | F value | Pr (>F) |
| --- | --- | --- | --- | --- | --- |
| Group | 2 | 31.67 | 15.837 | 6.944 | 0.004 |
| Residuals | 27 | 61.58 | 2.281 |  |  |

*p=<0.05

**S27 Table. Results of post-hoc Tukey pair-wise comparison for δ^13^C (VPDB) by Site/Phase.**

95% confidence interval of difference is indicated alongside 95% probability of lower and upper bounds of this difference.

| Group | Difference | Lower | Upper | *p*-value* |
| --- | --- | --- | --- | --- |
| KDK1 MN H – KDK1 K H | 2.527 | 0.844 | 4.210 | 0.003 |
| KDK21 MN H – KDK1 K H | 1.290 | -0.430 | 3.011 | 0.170 |
| KDK21 MN H – KDK1 MN H | -1.237 | -2.873 | 0.399 | 0.166 |

*p=<0.05

**S28 Table. Existing human stable isotopic (ẟ^13^C, ẟ^18^O and ẟ^15^N) data from relevant Egyptian and Upper Nubian Nile Valley sites prior to New Kingdom conquest 1500 BCE.**

Values reported in per mil (‰), *n* refers to number of individuals studied, standard deviation = σ1. * Isotopic values derived from keratin.

| Sample | Site | Chronological Period and Age | Mean ẟ^15^N collagen | Mean ẟ^13^C collagen | Mean ẟ^13^C carbonate | Mean ẟ^18^O carbonate |
| --- | --- | --- | --- | --- | --- | --- |
| **Egyptian** | | | | | | |
| Bone | El-Badari (n=3)^[32]^ | Predynastic (5500–3100 BCE) | 12.6 ± 0.4 | -19.2 ± 0.4 | – | – |
| Bone | Naqada (n=4)^[32]^ | Predynastic (5500–3100 BCE) | 12.5 ± 1.2 | -18.7 ± 0.3 | – | – |
| Bone | Hierakonpolis (n=1)^[32]^ | Predynastic (5500–3100 BCE) | 13.4 | -20.5 | – | – |
| Bone | Gebelein (n=3)^[33]^ | Predynastic (5500–3100 BCE) | 12.1 ± 1.0 | -19.4 ± 0.2 | -14.2 ± 0.7 | 31.6 ± 0.4 |
| Bone | Gebelein (n=6)^[33]^ | 1st Intermediate period (2160–2025 BCE) | 12.9 ± 0.9 | -19.4 ± 0.3 | -14.1 ± 0.3 | 31.4 ± 0.5 |
| Bone | Asyut (n=8)^[33]^ | 1st Intermediate period (2160–2025 BCE) | 13.0 ± 1.0 | -19.8 ± 0.4 | -14.7 ± 0.4 | 31.0 ± 0.6 |
| Bone | Abydos (n=2)^[32]^ | 12^th^ Dynasty (1991–1786 BCE) | 13.0 ± 0.8 | -18.4 ± 0.1 | – | – |
| **Upper Nubian** | | | | | | |
| Bone | R12 (n=21)^[34]^ | Middle Neolithic (4920–4320 BCE) | – | – | -9.8 ± 1.1 | 30.9 ± 0.8 |
| Hair* | KDK1 (n=1)^[19]^ | Kerma *Ancien* (2092 BCE) | 12.0 | -17.0 | – | – |
| Bone | Kerma (n=5)^[34, 35]^ | Kerma *Ancien* (2500–2050 BCE) | 12.2 ± 0.7 | -16.3 ± 1.3 | -11.7 ± 1.5 | 33.0 ± 0.6 |
| Bone | Kerma (n=6)^[34, 35]^ | Kerma *Moyen* (2050–1750 BCE) | 13.0 ± 1.0 | -19.7 ± 0.9 | -13.1 ± 0.4 | 32.9 ± 0.3 |
| Bone | Kerma (n=2)^[34, 35]^ | Kerma *Classique* (1750–1450 BCE) | 12.0 ± 2.1 | -20.3 ± 3.8 | -13.0 ± 0.3 | 32.8 ± 0.2 |
| Bone | Kerma (n=48)^[36]^ | Kerma *Classique* (1750–1450 BCE) | 13.9 ± 0.7 | -17.7 ± 1.6 | – | – |

**References**

1. Simon C. Notes anthropologiques sur les restes humains de Kadruka. Archéologie du Nil Moyen. 1987;2:63-7.

2. Maines E. Diversité biologique et archéologie de la mort: Une approche populationnelle et culturelle du Néolithique soudanais (Haute-Nubie). Paris: Université Panthéon-Sorbonne; 2019.

3. Langlois O, Chambon P, Sellier P. Trente-cinq ans d’archéologie préhistorique à Kadrouka. Cimetières et habitats néolithiques dans une concession menacée. In: Maillot M, editor. 50 ans d’archéologie française au Soudan. Paris, Khartoum: Africae, Soleb, Bleu autour; 2020. p. 98-125.

4. Bruzek J. A method for visual determination of sex, using the human hip bone. Am J Phys Anthropol. 2002;117(2):157-68, doi: 10.1002/ajpa.10012

5. Bruzek J. Fiabilité des processus de détermination du sexe à partir de l’os coxal: Implications à l’étude du dimorphisme sexuel de l’homme fossile: Musée National d’Histoire Naturelle de Paris: Institut de Paléontologie Humaine 1991.

6. Schmitt A. Une nouvelle méthode pour estimer l’âge au décès des adultes à partir de la surface sacro-pelvienne iliaque. Bulletins et mémoires de la Société d'anthropologie de Paris. 2005;17(1-2):89-101, doi: 10.4000/bmsap.943

7. Schmitt A. Variabilité de la sénescence du squelette humain, Réflexions sur les indicateurs de l’âge au décès: à la recherche d’un outil performant: Université Bordeaux 1; 2001.

8. Moorrees CFA, Fanning EA, Hunt EE. Formation and resorption of three deciduous teeth in children. Am J Phys Anthropol. 1963;21(2):205-13, doi: 10.1002/ajpa.1330210212

9. Moorrees CFA, Fanning EA, Hunt EE. Age variation of formation stages for ten permanent teeth. J Dent Res. 1963;42(6):1490-502, doi: 10.1177/00220345630420062701

10. Roche D, Ségalen L, Balan E, Delattre S. Preservation assessment of Miocene–Pliocene tooth enamel from Tugen Hills (Kenyan Rift Valley) through FTIR, chemical and stable-isotope analyses. J Archaeol Sci. 2010;37(7):1690-9, doi: 10.1016/j.jas.2010.01.029

11. Kellner CM, Schoeninger MJ. A simple carbon isotope model for reconstructing prehistoric human diet. Am J Phys Anthropol. 2007;133(4):1112-27, doi: 10.1002/ajpa.20618

12. Chaix L. Rapport preliminaire sur la faune du site de Kadruka I, Soudan Nord (Neolithique et Protohistorique). Archéologie du Nil Moyen. 1987;2:61-2.

13. Parr J. A comparison of heavy liquid floatation and microwave digestion techniques for the extraction of fossil phytoliths from sediments. Rev Palaeobot Palynol. 2002;120(3-4):315-36, doi: 10.1016/S0034-6667(01)00138-5

14. Duncan N, Pearsall DM, Benfer R. Gourd and squash artifacts yield starch grains of feasting foods from preceramic Peru. PNAS. 2009;106:13202-6, doi: 10.1073/pnas.0903322106

15. Pearsall DM, Chandler-Ezell K, Zeidler JA. Maize in ancient Ecuador: Results of residue analysis of stone tools from the Real Alto site. J Archaeol Sci. 2004;31(4):423-42, doi: 10.1016/j.jas.2003.09.010

16. Perry L, Sandweiss D, Piperno DR, Rademaker K, Malpass M, Umire A, et al. Early maize agriculture and interzonal interaction in southern Peru. Nature. 2006;440:76-9, doi: 10.1038/nature04294

17. Reimer P, Austin W, Bard E, Bayliss A, Blackwell P, Bronk Ramsey C, et al. The IntCal20 northern hemisphere radiocarbon age calibration curve (0–55 cal kBP). Radiocarbon. 2020;62(4):725-57, doi: 10.1017/RDC.2020.41

18. Bronk Ramsey C. Bayesian analysis of radiocarbon dates. Radiocarbon. 2009;51:337-60, doi: 10.1017/S0033822200033865

19. Bleasdale M, Richter KK, Janzen A, Brown S, Scott A, Zech J, et al. Ancient proteins provide evidence of dairy consumption in eastern Africa. Nat Commun. 2021;12(1):632, doi: 10.1038/s41467-020-20682-3

20. O’Connell TC. The isotopic relationship between diet and body proteins: implications for the study of diet in archaeology [Thesis]: University of Oxford; 1996.

21. Reinold J. Kadruka. In: Welsby DA, Anderson JR, editors. Sudan ancient treasures: An exhibition of recent discoveries from the Sudan National Museum. London: British Museum Press; 2004. p. 42-8.

22. Reinold J. Kadruka and the Neolithic in the Northern Dongola Reach. Sudan & Nubia. 2001;5:2-10.

23. Jaccard P. The distribution of the flora in the alpine zone. New Phytol. 1912;11(2):37-50.

24. Clarke K. Nonparametric multivariate analyses of changes in community structure. Austral Ecology. 1993;18:117-43, doi: 10.1111/j.1442-9993.1993.tb00438.x

25. LeGeros RZ. Calcium phosphates in oral biology and medicine. Basel; New York: Karger; 1991.

26. Michel V, Ildefonse P, Morin G. Chemical and structural changes in *Cervus elaphus* tooth enamels during fossilization (Lazaret Cave) a combined IR and XRD Rietveld analysis. Applied geochemistry. 1995;10(2):145-59, doi: 10.1016/0883-2927(95)00001-Z

27. Sponheimer M, Lee-Thorp JA. Alteration of enamel carbonate environments during fossilization. J Archaeol Sci. 1999;26(2):143-50, doi: 10.1006/jasc.1998.0293

28. Trueman CN, Privat K, Field J. Why do crystallinity values fail to predict the extent of diagenetic alteration of bone mineral? Palaeogeogr Palaeoclimatol Palaeoecol. 2008;266(3-4):160-7, doi: 10.1016/j.palaeo.2008.03.038

29. Shemesh A. Crystallinity and diagenesis of sedimentary apatites. Geochimica et Cosmochimica Acta. 1990;54(9):2433-8, doi: 10.1016/0016-7037(90)90230-I

30. Weiner S, Bar-Yosef O. States of preservation of bones from prehistoric sites in the Near East a survey. J Archaeol Sci. 1990;17(2):187-96, doi: 10.1016/0305-4403(90)90058-D

31. Sponheimer M. Isotopic paleoecology of the Makapansgat Limeworks fauna: The State University of New Jersey; 1999.

32. Thompson AH, Richards MP, Shortland A, Zakrzewski SR. Isotopic palaeodiet studies of ancient Egyptian fauna and humans. J Archaeol Sci. 2005;32(3):451-63, doi: 10.1016/j.jas.2004.11.004

33. Iacumin P, Bocherens H, Mariotti A, Longinelli A. An isotopic palaeoenvironmental study of human skeletal remains from the Nile Valley. Palaeogeogr Palaeoclimatol Palaeoecol. 1996;126(1-2):15-30, doi: 10.1016/S0031-0182(96)00067-3

34. Iacumin P. Stable isotopes as dietary indicators of Neolithic Nubian population. In: Salvatori S, Donatella U, editors. A Neolithic cemetery in the Northern Dongola Reach: Excavations at site R12. London: The Sudan Archaeological Research Society; 2008. p. 113-22.

35. Iacumin P, Bocherens H, Chaix L, Marioth A. Stable carbon and nitrogen isotopes as dietary indicators of ancient Nubian populations (northern Sudan). J Archaeol Sci. 1998;25(4):293-301, doi: 10.1006/jasc.1997.0206

36. Thompson AH, Chaix L, Richards M. Stable isotopes and diet at ancient Kerma, Upper Nubia (Sudan). J Archaeol Sci. 2008;35(2):376-87, doi: 10.1016/j.jas.2007.03.014
